# Power management for triboelectric and electrostatic generators enabling continuous and stable power delivery

**DOI:** 10.1038/s41467-026-72323-w

**Published:** 2026-04-25

**Authors:** Zeyuan Cao, Junchi Teng, Yongjia Zhao, Xingqi Guo, Jiani Xu, Chao Ren, Xiongying Ye

**Affiliations:** State Key Laboratory of Precision Measurement Technology and Instruments, Department of Precision Instrument, Tsinghua University, Beijing, China

**Keywords:** Devices for energy harvesting, Electrical and electronic engineering

## Abstract

Power management is essential for effectively utilizing energy harvested from triboelectric and electrostatic generators. However, current strategies suffer from control and conversion challenges such as inefficient switch designs and load-dependent outputs, resulting in substantial performance degradation when powering practical electronics. Here, we present an efficient and universal power management strategy to improve energy utilization. It features a self-powered peak-detection switch for enhancing energy harvesting, a voltage elevation strategy to improve AC–DC conversion, and an energy regulation module that dynamically allocates the harvested energy to ensure a continuous and stable power supply. Benefiting from the switch’s power-boosting mechanism, the output power after power management reaches 1.08 times the generator’s optimal AC output. This enables the reliable operation of a 20 dBm LoRa node powered by a 55-mm-radius electret generator at 36 rpm. This work facilitates the integration of triboelectric and electrostatic generators into practical electronic systems.

## Abstract (web summary)

The work presents a power management system for triboelectric and electrostatic generators, featuring a self-powered peak detection switch, a buck converter, and an energy regulation module. It enhances energy extraction, conversion, and utilization, enabling stable power supply for electronic devices.

## Introduction

With the rapid growth of Internet of Things (IoT) devices, wearables, and distributed sensor nodes, harvesting environmental energy for sustainable power supply has attracted increasing attention^1–3^. Triboelectric nanogenerators (TENGs) and electrostatic generators^4,5^, which convert mechanical energy into electricity primarily through electrostatic induction, have demonstrated promising capabilities in harvesting energy from micro-motions and low-frequency sources^6–9^. With advantages such as low cost, lightweight, and easy fabrication^10–12^, they have gained great attention for harvesting energy from various sources such as wind^13^, water drops^14,15^, vibration^16^, ocean waves^17^, and human motion^18^. However, due to their intrinsic capacitive nature, triboelectric and electrostatic generators exhibit a high internal impedance typically ranging from tens to thousands of MΩ, causing severe impedance mismatch and limited energy transfer efficiency when powering conventional electronics. Moreover, their high-voltage alternating-current (AC) output prevents them from directly powering electronic devices that require a stable, low-voltage direct-current (DC) supply. Efficient power management is critical for facilitating the practical application of triboelectric and electrostatic generators.

To date, various power management strategies, including rectifiers^19,20^, inductive transformers^21^, and switch-based step-down converters^22^, have been developed to convert the high-voltage AC output of triboelectric and electrostatic generators into low-voltage DC supply. Among these, switch-based step-down converters, such as switched-capacitor-converters^23,24^, the synchronized switch harvesting on inductor (SSHI) topology^25^, and LC buck converters^8,26–28^, demonstrate high efficiency. Their synchronous switching mechanism enables generators to operate in cycles for maximized energy output (CMEO)^29^, boosting the output while reducing the impedance mismatch between the generator and the subsequent circuit. Up to now, various kinds of switches, including mechanical switches, voltage-triggered switches, and transistor-based peak-detection switches, have been developed. Among these, the mechanical switches^30–32^ demonstrate the highest reported efficiency to date. However, their efficient operation is typically limited to voltages below 400 V due to air breakdown^33^, and they suffer from potential energy loss caused by mechanical friction, contact bounce, and breakdown-induced ablation^7^, which undermine their long-term reliability. Voltage-triggered switches^34–36^, while efficient, require customized design or auxiliary components like external capacitors for voltage matching to achieve optimal efficiency, making them susceptible to voltage deviations in practical environments. Transistor-based peak-detection strategies^27,37–39^enable accurate peak detection for synchronous switching but often rely on complex control circuits with high energy consumption and parasitic leakage. Recently, a self-powered transistor-based peak-detection switch has been proposed to simplify the control process and enable autonomous operation^40^. However, it still suffers from a limited operating-voltage range, a non-negligible loading effect, and a slow switching response. Therefore, an efficient and universal switching strategy remains desperately needed. Additionally, while current power management strategies successfully convert electrostatic generators’ high-voltage AC to DC output, the resulting DC is still unstable and load-dependent. The optimal performance can only be obtained at a matched load. In practical applications where loads deviate from matched resistance, the conversion efficiency decreases significantly to merely a few tens of percent of its maximum^8,24,39^. Meanwhile, the load-dependent voltage swing risks either damaging electronics during overvoltage or failing to activate devices during under-voltage when the load is mismatched. Recently, several approaches have been developed to stabilize voltage and enable reliable powering of conventional electronics, such as integrating a secondary DC–DC converter^41–43^, employing a hysteresis energy release control^26^, or storing energy in voltage-stable Li-ion batteries^44,45^. However, compared to the generators’ intrinsic output, the amount of energy being finally utilized by practical devices remains limited. Some multi-input single-inductor multi-output (MISIMO) systems^46–48^ with integrated topologies and intelligent control algorithms demonstrate effective energy regulation. However, they are typically designed for low-voltage sources such as thermoelectric, photovoltaic, and piezoelectric energy harvesters, which usually deliver only a few volts, and are not suitable for high-voltage triboelectric or electrostatic generators.

Herein, we propose a universal and self-powered power management system for high-voltage triboelectric and electrostatic generators. It delivers an output power comparable to or higher than the generator’s optimal AC output, while providing a stable DC supply compatible with conventional electronics as well as enabling on-demand energy storage and access. This is achieved through three main design advances. First, a self-powered peak detection switch is developed that is adaptable to generators with varying voltages, frequencies, and physical dimensions, enhancing energy harvesting and extraction. Second, we systematically analyze the AC–DC conversion losses in passive LC buck converters and introduce a voltage elevation scheme to reduce energy transfer losses. Third, an energy regulation module is proposed that dynamically allocates harvested energy between the load and backup battery, ensuring a stable, continuous DC supply. With the proposed power management system, a 20 dBm LoRa sensing node, which monitors five parameters and transmits data every 30 s, achieves continuous operation powered by a 55-mm-radius electret generator operating at 36 rpm. Moreover, surplus energy accumulated during 2.5 h of operation at 300 rpm supports the node’s continued operation for over 13.5 h after the generator stops. This power management system facilitates the use of triboelectric and electrostatic generators as stable power sources for electronic devices.

## Results

### Power management strategy for efficient energy extraction, conversion, and utilization

**Fig. 1:**
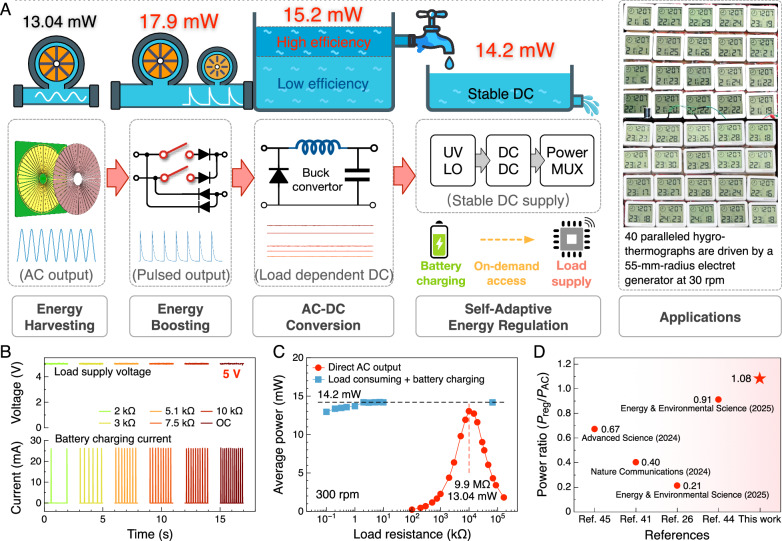
Overview of the power management system for continuous and stable power delivery. **A** Schematic of the power management system for simultaneous stable load supply, backup battery charging, and on-demand energy access. UVLO under-voltage lockout, DC–DC direct-current to direct-current (converter), Power MUX power multiplexer, AC alternating current, DC direct current. **B** Load supply voltage and corresponding battery charging current under varying load resistances. OC open circuit. **C** Comparison of the generator’s direct AC output and the regulated DC output after power management, including load consumption and battery charging. **D** Comparison of the power management performance presented in this work with previous works. Source data are provided as a Source Data file.

High performance power management of triboelectric and electrostatic generators relies on three key processes: efficient energy extraction, conversion, and utilization. As shown in Fig. 1A, a self-powered peak-detection switch is proposed to enable efficient energy extraction. The switch closes at exactly the generator’s output peak, allowing the generator to operate in CMEO and converting the generator’s continuous AC output to discrete high-energy pulses (Supplementary Fig. 1). This mechanism increases the output power from 13.04 to 17.9 mW while enabling wide load compatibility. Subsequently, a passive LC buck converter is employed for AC–DC conversion. To address the load-dependent nature and efficiency decrease at low output voltages, the energy losses in the buck circuits were analyzed, and a voltage elevation strategy was introduced to enhance AC–DC conversion efficiency. This strategy is integrated into the energy regulation module by implementing an under-voltage lockout (UVLO) circuit, yielding a 15.2 mW output from the LC buck converter. To enable efficient energy utilization, the energy regulation module further incorporates a step-down DC–DC converter for stable voltage output and a self-adaptive power multiplexer (MUX) that dynamically allocates the harvested energy between the load and the backup battery according to operating conditions. Together, these components form a power management system that enables efficient AC–DC conversion for triboelectric and electrostatic generators, providing a continuous and stable output of 14.2 mW for driving practical applications. Detailed experimental data collected during the addition of each power management sub-module are shown in Supplementary Fig. 2. The final output of the power management system is shown in Fig. 1B. The total regulated power output after management reaches 1.08 times the generator’s optimal AC power (Fig. 1C), demonstrating superior performance over previous works (Fig. 1D, Supplementary Table 1). A detailed comparison of the proposed power management system with previous studies is presented in Supplementary Table 2 and further elaborated in Supplementary Note 1 and Note 2.

To make our report clear, this work is structured as follows: First, we develop a self-powered peak-detection switch to maximize energy extraction. Second, we systematically analyze the energy loss mechanisms in LC buck converters to identify optimization pathways for efficient AC–DC conversion. Third, we experimentally verify the efficiency and robustness of the developed switch and the optimized LC buck converter across a diverse range of practical generator configurations. Finally, we develop an energy regulation module to achieve efficient energy utilization and demonstrate its practical utility through various applications.

### Self-powered peak-detection switch for efficient energy extraction

**Fig. 2:**
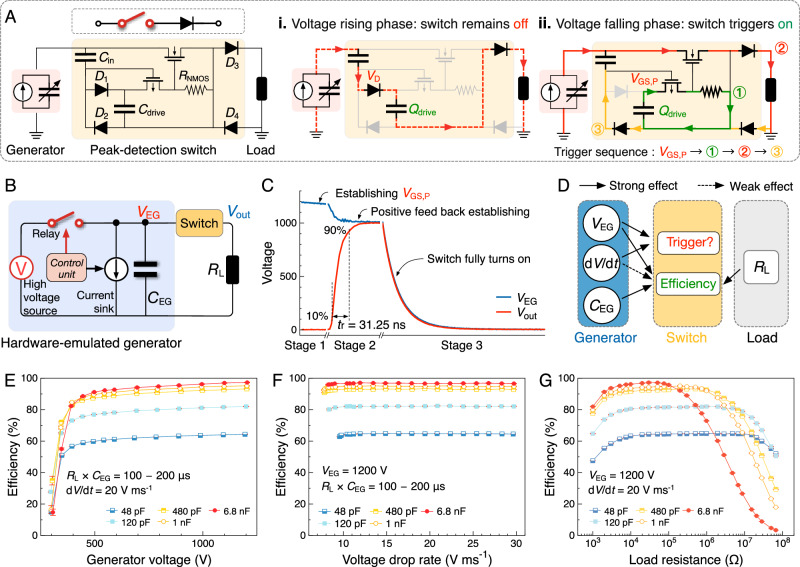
Working principle and performance of the self-powered peak-detection switch. **A** Schematic and working principle of the self-powered peak-detection switch. **B** Schematic of the test platform for the switch. **C** Voltage waveforms during switch closure, depicted at different time scales to highlight the triggering process. **D** Factors affecting the performance of the switch. **E–G** Switch efficiency at various generator voltages (${V}_{{{{\rm{EG}}}}}$), voltage drop rates (${{{\rm{d}}}}V$*/*${{{\rm{d}}}}t$), and load resistances (${R}_{{{{\rm{L}}}}}$) under different generator capacitances (${C}_{{{{\rm{EG}}}}}$). Efficiency values are presented as the mean of *n* = 5 independent experiments. Error bars represent s.d. Source data are provided as a Source Data file.

Figure 2A shows the schematic and working principle of the proposed self-powered peak-detection switch for efficient energy extraction. The switch employs a low-gate-charge N-MOSFET as the main switching element, which is triggered when a voltage peak is detected at the generator output. The switch’s operation can be divided into two phases, corresponding to the rising phase (Fig. 2A(i)) and the falling phase (Fig. 2A(ii)) of the generator voltage. During the rising phase, the N-MOSFET remains in off state, and the charge for driving the N-MOSFET is accumulated on ${C}_{{{{\rm{d}}}}{{{\rm{rive}}}}}$ through the ${C}_{{{{\rm{in}}}}}$ → ${D}_{1}$ → ${C}_{{{{\rm{d}}}}{{{\rm{rive}}}}}$ → ${D}_{3}$→load path. This charge transfer also generates a positive gate-source bias on the P-MOSFET due to the ${D}_{1}$’s intrinsic forward voltage drop (${V}_{{{{\rm{D}}}}}$), maintaining both the P- and N-MOSFETs in the off state. The charge accumulated in ${C}_{{{{\rm{d}}}}{{{\rm{rive}}}}}$ is given by ${Q}_{{{{\rm{d}}}}{{{\rm{rive}}}}}$ = ${C}_{{{{\rm{in}}}}}$ × ${V}_{{{{\rm{EG}}}}}$. Then, after the input voltage reaches its peak and starts to decrease, ${D}_{1}$ turns off. A negative gate-source voltage then develops across the P-MOSFET and quickly approaches its turn-on threshold (Fig. 2C, Stage 1). The P-MOSFET then turns on, allowing ${C}_{{{{\rm{d}}}}{{{\rm{rive}}}}}$ to turn on the N-MOSFET (Fig. 2A(ii), green loop), enabling energy transfer from the generator to the load (Fig. 2A(ii), red loop). This triggering process functions as a positive feedback mechanism: N-MOSFET conduction enhances P-MOSFET conduction by reducing ${V}_{{{{\rm{EG}}}}}$, which further reinforces N-MOSFET conduction. This leads to an ultrafast switching speed of less than 40 ns (Fig. 2C, Stage 2) and enhances the sensitivity of peak detection. During switch closure, the charge stored in ${C}_{{{{\rm{in}}}}}$ is also transferred to the load when ${V}_{{{{\rm{EG}}}}}$ falls below the voltage of ${C}_{{{{\rm{in}}}}}$, resetting the switch state and enabling the next cycle (Fig. 2A(ii), yellow loop). Notably, the switch trigering is determined solely by two conditions: sufficient charge accumulation before the voltage peak and a subsequent voltage drop, making it essentially independent of the generator capacitance. Therefore, as long as these trigger conditions are met, the switch can operate reliably even with varying generator capacitance.

For a peak-detection switch, adaptability to different generator configurations is crucial. To better understand the switch’s performance and enhance its adaptability, a standard test platform is introduced, as shown in Fig. 2B. The test platform features a hardware-emulated generator that consists of a programmable voltage source to modulate the generator voltage (${V}_{{{{\rm{EG}}}}}$), a film capacitor to emulate the generator capacitance (${C}_{{{{\rm{EG}}}}}$), and a controllable constant current sink to regulate the post-peak voltage drop rate (${{{\rm{d}}}}V$/${{{\rm{d}}}}t$), simulating the generator’s frequency-dependent behavior. During testing, the hardware-emulated generator triggers the peak-detection switch by first fully charging of ${C}_{{{{\rm{EG}}}}}$ and then discharging it at a constant-rate, reproducing the peak event of a real generator. The test time sequence is shown in Supplementary Fig. 3, with photographs of the test platform and the switch presented in Supplementary Figs. 4 and 5, respectively. The default parameters of the switch and the test platform used in the experiment are listed in Supplementary Table 3. The switch efficiency is defined as the ratio of the energy released to ${R}_{{{{\rm{L}}}}}$ after switching (${E}_{{{{\rm{out}}}}}$) to the total energy accumulated before the switch turns on (${E}_{{{{\rm{in}}}}}$):

1 $${\eta }_{{{{\rm{sw}}}}}=\frac{{E}_{{{{\rm{out}}}}}}{{E}_{{{{\rm{in}}}}}}=\frac{\int {V}_{{{{\rm{L}}}}}^{2}/{R}_{{{{\rm{L}}}}}{{{\rm{d}}}}t}{0.5\times \left({C}_{{{{\rm{EG}}}}}+{C}_{{{{\rm{in}}}}}+{C}_{{{{\rm{DS}}}},{{{\rm{NMOS}}}}}\right)\times {V}_{{{{\rm{EG}}}},{{{\rm{peak}}}}}^{2}}$$

where ${V}_{{{{\rm{L}}}}}$ is the load voltage, ${R}_{{{{\rm{L}}}}}$ is the load resistance, ${C}_{{{{\rm{EG}}}}}$ is the emulated generator’s capacitance, ${C}_{{{{\rm{in}}}}}$ is the input capacitance of the switch, ${C}_{{{{\rm{DS}}}},{{{\rm{NMOS}}}}}$ is the drain-source capacitance of the N-MOSFET, and ${V}_{{{{\rm{EG}}}},{{{\rm{peak}}}}}$ is the peak voltage across ${C}_{{{{\rm{EG}}}}}$.

The switch efficiency was measured for various generator voltages, voltage drop rates, and load resistances under different generator capacitances, with detailed results in Supplementary Figs. 6–20 and summarized results in Fig. 2E–G. The results show that the switch efficiency increases monotonically with generator capacitance ${C}_{{{{\rm{EG}}}}}$, which is typically determined by the generator’s physical size, reaching over 75% for capacitances above 120 pF and exceeding 90% above 480 pF. This trend arises because the energy required to drive the switch remains constant (determined by ${C}_{{{{\rm{in}}}}}$ and ${V}_{{{{\rm{EG}}}}}$); thus, a larger ${C}_{{{{\rm{EG}}}}}$, which stores more total energy, results in lower relative energy consumption. Additionally, larger ${C}_{{{{\rm{EG}}}}}$ mitigates the impact of parasitic capacitance after the switch (e.g., wiring), enhancing the overall energy transfer efficiency. Therefore, a larger ${C}_{{{{\rm{EG}}}}}$ design is generally preferred. Besides ${C}_{{{{\rm{EG}}}}}$, the switch also adapts well to a wide range of generator voltage ${V}_{{{{\rm{EG}}}}}$, maintaining consistently high efficiency above 500 V (Fig. 2E). This efficiency shows a modest increase with ${V}_{{{{\rm{EG}}}}}$, rising by about 5% from 500 to 1200 V. This trend occurs because, at higher ${V}_{{{{\rm{EG}}}}}$, the voltage drop after switch closure constitutes a smaller fraction of the total voltage (Supplementary Figs. 6D–10D). Below 500 V, the efficiency declines sharply due to insufficient energy to drive the switch (determined by ${C}_{{{{\rm{in}}}}}$ and ${V}_{{{{\rm{EG}}}}}$), leading to a lower switching speed (Supplementary Figs. 6F–10F) and a shorter switch closure time. Ultimately, the switch fails to operate below 300 V. Increasing ${C}_{{{{\rm{in}}}}}$ (Supplementary Fig. 22) or removing the capacitor divider (Supplementary Fig. 23) can mitigate this trigger failure by enhancing the available driving charge with lower generator voltages, extending the switch’s operating range. The switch also demonstrates robust performance across various voltage drop rates ${{{\rm{d}}}}V$/${{{\rm{d}}}}t$, which typically are determined by the generator’s operating frequency. Once triggered, the switch maintains constantly high efficiency (Fig. 2F) and achieves a switching time as short as 30–50 ns (Supplementary Figs. 11F–15F). However, if the voltage drop rate of ${V}_{{{{\rm{EG}}}}}$ falls below a critical threshold, the switch fails to trigger. This occurs because an insufficient voltage drop rate leads to incomplete P-MOSFET conduction, which increases the resistance between ${C}_{{{{\rm{drive}}}}}$ and ${R}_{{{{\rm{NMOS}}}}}$ and reduces the gate-source potential of the N-MOSFET, preventing the initiation of positive feedback for switch activation. Moderately increasing ${C}_{{{{\rm{in}}}}}$, ${C}_{{{{\rm{drive}}}}}$, or ${R}_{{{{\rm{NMOS}}}}}$ can reduce the charge leakage in the circuit and facilitate switch triggering at lower voltage drop rates, as demonstrated in Supplementary Fig. 24. Regarding the adaptability to load resistance ${R}_{{{{\rm{L}}}}}$, the switch maintains high efficiency at moderate values, with a slight decline at excessively high or low values (Fig. 2G) due to limited closure time at high ${R}_{{{{\rm{L}}}}}$ and increased relative impact of the MOSFET’s on-resistance at low ${R}_{{{{\rm{L}}}}}$, as detailed in the note below Supplementary Fig. 16. Extending switch closure time by increasing ${C}_{{{{\rm{drive}}}}}$ or ${R}_{{{{\rm{NMOS}}}}}$, or increasing driving charge by increasing ${C}_{{{{\rm{in}}}}}$, can improve its adaptability to both load resistance extremes, as illustrated in Supplementary Fig. 25. The effects of the generator parameters and load resistances on the performance of the switch are summarized in Fig. 2D.

Overall, the proposed self-powered peak-detection switch demonstrates broad adaptability to a wide range of generator capacitances, voltages, voltage drop rates, and load resistances. Together with the tunability from internal parameter modifications, these features enable efficient power extraction across diverse energy harvesting scenarios.

### Loss analysis and performance optimization of LC buck converter for efficient energy conversion

**Fig. 3:**
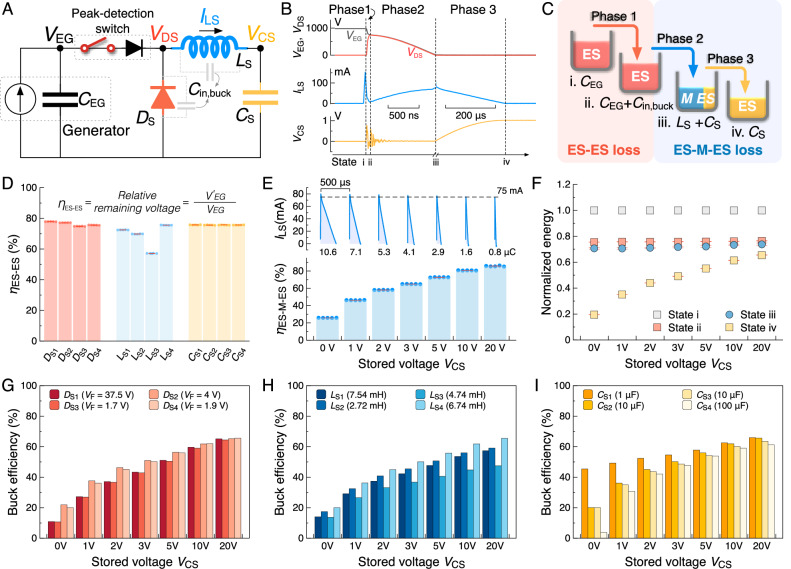
Working principle and loss analysis of the LC buck converter. **A** Schematic of the LC buck converter. **B** Voltage and current waveforms during an energy transfer cycle (${C}_{{{{\rm{EG}}}}}$ = 47 pF, ${V}_{{{{\rm{EG}}}}}$ = 1000 V, ${V}_{{{{\rm{C}}}}{{{\rm{S}}}}}$ = 0). **C** Illustration of the three energy transfer phases and their corresponding four states within a single transfer cycle. ES, electrostatic energy; M, magnetic energy. **D** Energy transfer efficiency during the electrostatic-to-electrostatic conversion phase (Phase 1), denoted as ${\eta }_{{ES}-{ES}}$, for different buck converter configurations. **E** Energy transfer efficiency during the electrostatic-to-magnetic-to-electrostatic energy conversion phases (Phases 2 and 3), denoted as ${\eta }_{{ES}-{{{\rm{M}}}}-{ES}}$, for different stored voltages. **F** Normalized energy at different energy states (States i–iv in C) for different stored voltages. Efficiency and normalized energy values in (**D–F**) are presented as the mean of *n* = 5 independent experiments. Error bars represent s.d. **G–I** End-to-end efficiency across different buck converter parameters at various stored voltages. ${V}_{{{{\rm{F}}}}}$, forward voltage. Source data are provided as a Source Data file.

The self-powered peak-detection switch enables efficient energy extraction, but its high-voltage pulsed output necessitates conversion to low-voltage DC for compatibility with conventional electronics. Among step-down converters, the passive LC buck converter (Fig. 3A) exhibits optimal efficiency. However, the inherently capacitive nature of triboelectric and electrostatic generators leads to complex coupling dynamics with the buck converter. This complexity creates a major hurdle in developing a universal optimization framework across diverse generator configurations.

To address this issue, we systematically analyze the energy transfer losses in LC buck converters using the proposed peak-detection switch and the test platform, with experimental parameters listed in Supplementary Table 4. During a complete AC–DC conversion process, energy transfer proceeds through three phases and involves four distinct states (Fig. 3B, C). Energy losses occur at each phase, differing in mechanism. Initially, the harvested energy is stored in the generator capacitor ${C}_{{{{\rm{EG}}}}}$ as electrostatic (ES) energy (State i). Upon switch closure, energy transfers from ${C}_{{{{\rm{EG}}}}}$ to the combined capacitance of ${C}_{{{{\rm{EG}}}}}$ and the buck converter’s input capacitance ${C}_{{{{\rm{in}}}},{{{\rm{buck}}}}}$ (Phase 1, State i → ii). This process adheres to charge conservation, with energy loss arising from the voltage drop caused by the increased total capacitance. As this loss occurs within the electrostatic domain, it is termed an ES-ES transfer loss. After the switch fully closes, energy begins to be transferred from the combined capacitance to the inductor ${L}_{{{{\rm{S}}}}}$ and capacitor ${C}_{{{{\rm{S}}}}}$ (Phase 2, State ii → iii). During this phase, the majority of the electrostatic energy converts to magnetic (M) energy and is stored in ${L}_{{{{\rm{S}}}}}$ while the remaining fraction is directly transferred to ${C}_{{{{\rm{S}}}}}$, as evidenced by the decrease in ${V}_{{{{\rm{EG}}}}}$, the rise in ${I}_{{{{\rm{LS}}}}}$, and a slight increase in ${V}_{{{{\rm{CS}}}}}$. When ${V}_{{{{\rm{EG}}}}}$ reaches its minimum, the freewheeling diode ${D}_{{{{\rm{S}}}}}$ becomes forward-biased, allowing ${L}_{{{{\rm{S}}}}}$ to discharge its stored energy into ${C}_{{{{\rm{S}}}}}$ (Phase 3, State iii → iv). The energy loss across these two phases originates primarily from the conduction loss during the ES → M → ES conversion process and is thus termed ES-M-ES transfer loss.

The ES-ES and ES-M-ES transfer losses are governed by distinct mechanisms. The ES-ES loss (Phase 1) is device-dependent and primarily governed by ${C}_{{{{\rm{EG}}}}}$ and ${C}_{{{{\rm{in}}}},{{{\rm{buck}}}}}$. The energy transfer efficiency during the ES-ES process is expressed as:

2 $${\eta }_{{{{\rm{ES}}}}-{{{\rm{ES}}}}}=\frac{{C}_{{{{\rm{EG}}}}}}{{C}_{{{{\rm{EG}}}}}+{C}_{{{{\rm{in}}}},{{{\rm{buck}}}}}}=\frac{{V}_{{{{\rm{EG}}}}}^{{\prime} }}{{V}_{{{{\rm{EG}}}}}\,}$$

where ${V}_{{{{\rm{EG}}}}}$ and ${V}_{{{{\rm{EG}}}}\,}^{{\prime} }$ denote the voltages across *C*_EG_ before and after switch closure, respectively. ${C}_{{{{\rm{in}}}},{{{\rm{buck}}}}}$ mainly comes from the reverse-bias capacitance of the freewheeling diode ${D}_{{{{\rm{S}}}}}$ and the parasitic capacitance of the inductor ${L}_{{{{\rm{S}}}}}$ (Fig. 3A and Supplementary Note 3). Consequently, buck circuits employing different ${D}_{{{{\rm{S}}}}}$ or ${L}_{{{{\rm{S}}}}}$ components result in distinct ES-ES efficiencies, as demonstrated in Fig. 3D. As ${C}_{{{{\rm{S}}}}}$ is isolated well by ${L}_{{{{\rm{S}}}}}$, its variation has no influence on ${C}_{{{{\rm{in}}}},{{{\rm{buck}}}}}$ and ${\eta }_{{{{\rm{ES}}}}-{{{\rm{ES}}}}}$. Detailed specifications of the diodes ${D}_{{{{\rm{S}}}}}$, inductors ${L}_{{{{\rm{S}}}}}$, and capacitors ${C}_{{{{\rm{S}}}}}$ used in the experiments are summarized in Supplementary Table 4. They are selected to vary key values, such as the forward voltage drop of ${D}_{{{{\rm{S}}}}}$, the inductance of ${L}_{{{{\rm{S}}}}}$, and the capacitance of ${C}_{{{{\rm{S}}}}}$. Besides ${C}_{{{{\rm{in}}}},{{{\rm{buck}}}}}$, ${C}_{{{{\rm{EG}}}}}$ also governs *η*_ES-ES_ in Eq. 2. Increasing ${C}_{{{{\rm{EG}}}}}$ helps improve ES-ES efficiency, as shown in Supplementary Fig. 26A–C.

The ES-M-ES loss arises from the conduction loss across the freewheel diode (due to its forward voltage, ${V}_{{{{\rm{F}}}}}$), as well as the Joule heating caused by current flow through the inductor’s equivalent series resistance (${R}_{{{{\rm{LS}}}}}$) and other parasitic resistances (${R}_{{{{\rm{p}}}}}$), such as wiring. Assuming no inductor saturation, this loss is quantified as follows:

3 $${E}_{{{{\rm{loss}}}},{{{\rm{ES}}}}-{{{\rm{M}}}}-{{{\rm{ES}}}}}=\int ^{{t}_{4}}_{{t}_{2}}({R}_{{{{\rm{LS}}}}}+{R}_{{{{\rm{p}}}}}){\times i}^{2}\left(t\right){{{\rm{d}}}}t+\int ^{{t}_{4}}_{{t}_{3}}{V}_{{{{\rm{F}}}}}\times i\left(t\right){{{\rm{d}}}}t$$

where ${t}_{2}$, ${t}_{3}$, and ${t}_{4}$ represent the time points at which the system enters states ii, iii, and iv, respectively. Reducing ${V}_{{{{\rm{F}}}}}$ and resistances in the circuit can effectively mitigate the ES-M-ES loss and enhance the energy transfer efficiency. However, implementing such optimizations may necessitate component replacement, which can pose difficulties particularly when the circuit has been primarily optimized for ES-ES energy transfer. To circumvent these hardware modifications, a more practical alternative is to minimize the transferred charge (${Q}_{{{{\rm{trans}}}}}$) per unit of energy by operating at an elevated stored voltage (${V}_{{{{\rm{CS}}}}}$). This is because a capacitor’s stored energy scales quadratically with voltage, and higher ${V}_{{{{\rm{CS}}}}}$ reduces the charge needed to transfer equivalent energy (Supplementary Fig. 27). As shown in Fig. 3E, increasing ${V}_{{{{\rm{CS}}}}}$ from 0 to 20 V decreases ${Q}_{{{{\rm{trans}}}}}$ by an order of magnitude from more than 10 μC to less than 1 μC. This charge reduction drives ES-M-ES efficiency improvement from 25.5% at ${V}_{{{{\rm{CS}}}}}$ = 0 V to 85.6% at ${V}_{{{{\rm{CS}}}}}$ = 20 V.

Figure 3F presents the normalized energy at each state of the energy transfer process as ${V}_{{{{\rm{CS}}}}}$ increases under the default configuration (see Supplementary Note 4 for calculation details). The ES-ES loss, determined by intrinsic component characteristics, remains constant, while the ES-M-ES loss decreases with increasing ${V}_{{{{\rm{CS}}}}}$. As a result, total energy loss decreases, leading to improved conversion efficiency. These trends persist across all buck converter configurations, as shown in Fig. 3G–I, Supplementary Figs. 28 and 29. Notably, as ${V}_{{{{\rm{CS}}}}}$ increases, the dominant source of energy loss shifts from the ES-M-ES stage to the ES-ES stage. Consequently, the efficiency differences among the configurations gradually converge to the ES-ES limit shown in Fig. 3D. Since the ES-M-ES loss cannot be entirely eliminated even at ${V}_{{{{\rm{CS}}}}}$ = 20 V (Supplementary Fig. 29D–F), a slight gap remains between the buck and ES-ES efficiencies. However, this gap has been significantly reduced.

In addition, inductor saturation during the ES-M-ES phase is another key factor limiting buck conversion efficiency. It occurs when the transferred energy exceeds the saturation threshold of ${L}_{{{{\rm{S}}}}}$, such as under high ${C}_{{{{\rm{EG}}}}}$ conditions (Supplementary Figs. 26G–I and 30), or when using an ${L}_{{{{\rm{S}}}}}$ with a low saturation current (Supplementary Fig. 31). Although this saturation does not affect the ES-ES loss and can be partially mitigated by elevating ${V}_{{{{\rm{CS}}}}}$, it cannot be fully eliminated and continues to compromise the overall efficiency. A series inductor configuration provides an efficient means of mitigating saturation by increasing the total inductance and reducing the required current for a given energy transfer. Notably, this configuration also reduces ES-ES losses by lowering parasitic capacitance and further suppresses ES-M-ES losses by increasing the ${L}_{{{{\rm{S}}}}}$/${R}_{{{{\rm{LS}}}}}$ ratio when a series-aiding connection is made, as demonstrated in Supplementary Fig. 32.

In summary, achieving high-efficiency buck conversion in triboelectric and electrostatic energy harvesting systems can be achieved through three key strategies: configuring ${L}_{{{{\rm{S}}}}}$ to avoid saturation, optimizing components to minimize ES-ES loss, and maintaining a high ${V}_{{{{\rm{CS}}}}}$ to suppress ES-M-ES loss.

### Performance of the peak-detection switch and buck converter with practical generators

**Fig. 4:**
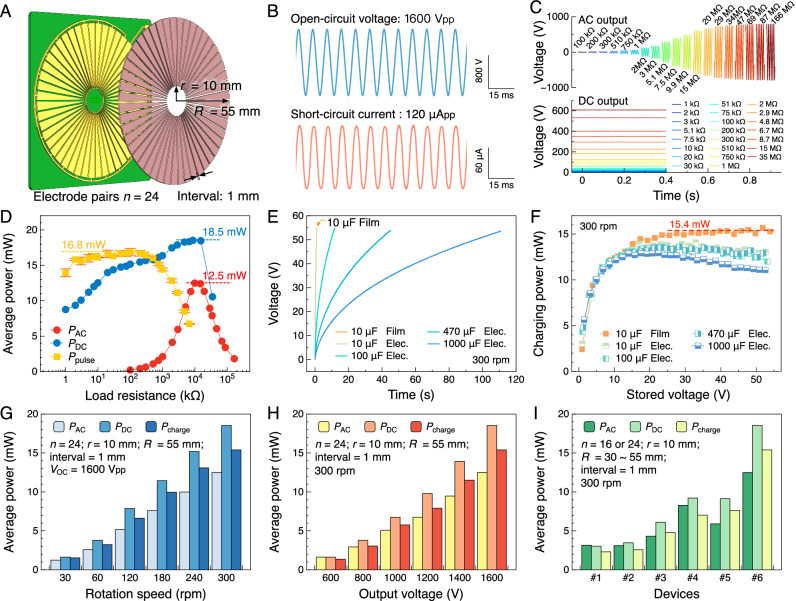
Performance of the self-powered peak-detection switch and LC buck converter with practical electrostatic generators. **A** Schematic of the rotational electret generator. **B** Open-circuit voltage and short-circuit current of the generator. **C** Direct AC output voltage of the generator and DC output voltage after the buck converter at 300 rpm. **D** Average output power as a function of load resistance for the direct AC output (${P}_{{{{\rm{AC}}}}}$), the pulsed output (${P}_{{{{\rm{pulse}}}}}$), and the DC output after the buck converter (${P}_{{{{\rm{DC}}}}}$). **E** Charging voltage curves of different storage capacitors. Elec., electrolytic capacitor. Film, film capacitor. **F** Charging power of different capacitors versus the stored voltage. **G–I** Comparison of the average output power of AC output (${P}_{{{{\rm{A}}}}{{{\rm{C}}}}}$), buck converter DC output (${P}_{{{{\rm{DC}}}}}$), and capacitor charging power (${P}_{{{{\rm{pulse}}}}}$) under different operating frequencies, output voltages, and generator configurations. ${V}_{{{{\rm{OC}}}}}$, open-circuit voltage. Source data are provided as a Source Data file.

To validate the practical applicability of the proposed switch and LC buck converter, we performed experiments using a rotational electret generator, as shown in Fig. 4. The generator consists of a printed circuit board (PCB) stator with fan-shaped electrode pairs and a rotor with a bipolar-charged electret film attached to an FR-4 substrate (Fig. 4A). Detailed dimensional parameters are listed in Supplementary Table 5, with the photograph shown in Supplementary Fig. 33A. Fabrication details are provided in the Methods section. Here, to accommodate the generator’s bipolar output, a switch topology with two peak-detection switches and rectifying diodes is developed. The schematic of this switch topology, combined with an LC buck converter, is shown in Supplementary Fig. 34. Note that this structure can also accommodate generators with asymmetric outputs (e.g., contact–separation mode TENGs), provided that each switch’s operating voltage range covers the generator voltage of its respective polarity. The switch’s operating voltage range can be adjusted by using a MOSFET with a higher drain–source voltage tolerance (e.g., a SiC MOSFET) to raise the upper limit, and by increasing the switch’s input capacitance (${C}_{{{{\rm{in}}}}}$) to lower the lower limit.

The open-circuit voltage and short-circuit current of the generator were measured at a rotation speed of 300 rpm, as shown in Fig. 4B. The direct AC output voltage and the DC output voltage after the buck converter are presented in Fig. 4C, and the pulsed output voltage after the peak-detection switch is shown in Supplementary Fig. 38G. Their corresponding average powers versus load resistance are summarized in Fig. 4D. Due to the energy enhancement effect of the switch, the pulsed output exhibits 1.34 times the average power of the optimal AC power at the matched load resistance. Note that the measured pulsed output values are lower than the true values due to the load-side parasitic capacitance during measurement, as detailed in Supplementary Fig. 35. Furthermore, the DC output after the buck converter reaches its maximum at a matched resistance of 15 MΩ, delivering 1.48 times the average power of the optimal AC output. These results highlight the superior performance of both the synchronous peak-detection switch and the LC buck converter. In addition to the average power delivered to load resistances, the charging power of the storage capacitor ${C}_{{{{\rm{S}}}}}$ is another key metric for evaluating the performance of the switch and LC buck converter. Figure 4E presents the measured voltage waveforms of storage capacitors with different capacitances and of different types. The corresponding charging power as a function of the stored voltage ${V}_{{{{\rm{CS}}}}}$ is shown in Fig. 4F. The charging power curve is derived by segmenting the waveform into discrete voltage increments, calculating the average power and voltage for each segment, and then aggregating the results, as illustrated in Supplementary Fig. 36. Consistent with the results in Fig. 3, the charging power in the experiment exhibits a ${V}_{{{{\rm{CS}}}}}$-dependent efficiency. As ${V}_{{{{\rm{CS}}}}}$ increases, the charging power of the storage capacitors increases monotonically, and the efficiency difference caused by capacitor variation is also mitigated. When ${V}_{{{{\rm{CS}}}}}$ exceeds 4 V, all capacitors exhibit similar charging power, consistent with the convergence trend shown in Fig. 3I. When ${V}_{{{{\rm{CS}}}}}$ exceeds 30 V, the charging power of the film capacitor approaches its maximum and exceeds the optimal AC output, demonstrating its high efficiency. In contrast, the charging power of electrolytic capacitors declines slightly above 20 V due to the increased leakage losses at higher voltages. Nevertheless, they still perform reasonably well compared with that at lower ${V}_{{{{\rm{CS}}}}}$.

To further validate the broad adaptability and excellent performance of the proposed switch and the buck converter, we evaluated their performance with the generator across varying operating frequencies, output voltages, and physical configurations (Supplementary Table 5, Supplementary Fig. 33). The results are summarized in Fig. 4G–I. Comprehensive experimental details, including open-circuit voltage, short-circuit current, direct AC output, DC output after the buck converter, and capacitor charging characteristics, are documented in Supplementary Figs. 37–49. These results show that the proposed switch with the buck converter achieves excellent performance across a wide range of generator conditions, delivering DC and charging power superior to the optimal AC output. Even under low-speed, low-voltage, and small-size conditions, the DC and charging power remain comparable to the optimal AC performance. These results demonstrate the high adaptability and efficiency of the proposed switch and LC buck converter.

### Self-adaptive energy regulation for efficient energy utilization

Although the buck converter produces DC output, its output power is still load-dependent and requires megohm-level loads to reach the optimum. This leads to an excessively high voltage under high-resistance loads or a reduced efficiency under low-resistance loads, potentially damaging electronic components or leading to driving failure.

**Fig. 5:**
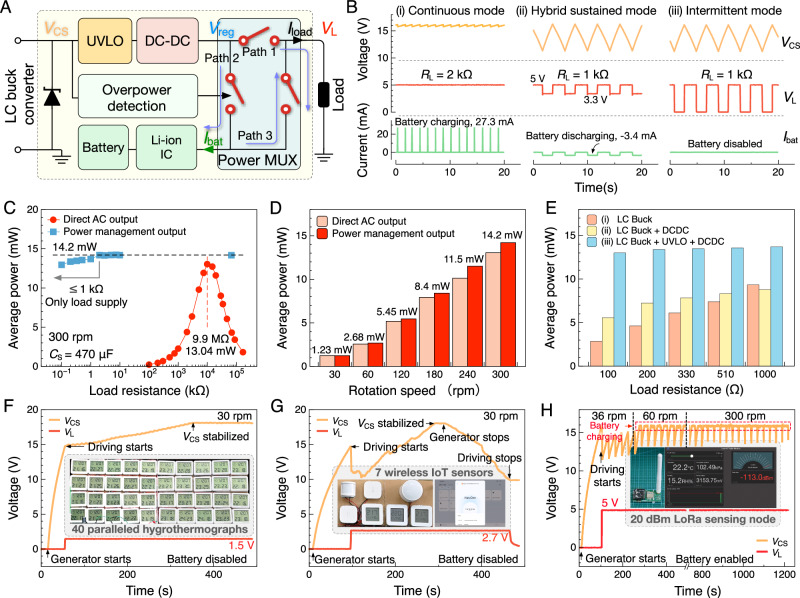
Performance of the energy regulation module and applications. **A** Schematic of the energy regulation module. UVLO, under-voltage lockout; DC–DC, direct current to direct current; Power MUX, power multiplexer. **B** Voltage and current waveforms in the energy regulation module under continuous, hybrid sustained, and intermittent operation modes. ${V}_{{{{\rm{CS}}}}}$, stored voltage; ${V}_{{{{\rm{L}}}}}$, load voltage, ${I}_{{{{\rm{load}}}}}$, load current, ${I}_{{{{\rm{bat}}}}}$, battery charging current. **C** Comparison of the output performance without power management and with power management. **D** Power management performance under different rotation speeds. **E** Performance comparison under different power management setups: (i) LC buck, (ii) LC buck + DCDC, and (iii) LC buck + UVLO + DCDC. **F** Demonstration of driving 40 hygrothermographs at 30 rpm (${C}_{{{{\rm{S}}}}}$ = 470 μF). **G** Demonstration of driving 7 wireless IoT sensors at 30 rpm (${C}_{{{{\rm{S}}}}}$ = 1 mF). **H** Demonstration of simultaneously driving a 20 dBm LoRa sensing node and charging a battery (${C}_{{{{\rm{S}}}}}$ = 1 mF). Source data are provided as a Source Data file.

To address this challenge, an energy regulation strategy is proposed, which comprises an under-voltage lockout (UVLO) module, a DC–DC converter, an overpower detection module, a power multiplexer unit, and a rechargeable backup battery, as shown in Fig. 5A. The detailed schematic and photograph of the energy regulation module are shown in Supplementary Figs. 50 and 51, respectively. The UVLO module optimizes buck conversion efficiency by sustaining the buck converter’s stored voltage (${V}_{{{{\rm{CS}}}}}$) at elevated levels. The subsequent DC–DC converter steps down this high voltage to the required lower level for direct load supply (Path 1) and backup battery charging (Path 2). The load supply path remains continuously active during the DC–DC converter operation, whereas the battery charging path is triggered by the overpower detection module only when the harvested energy exceeds load demands. Additionally, a backup supply path (Path 3) is activated during energy shortages, supplying power from the battery to the load. All energy path transitions are seamlessly regulated by the self-powered power multiplexer unit based on energy supply conditions. Figure 5B illustrates the typical working modes of the energy regulation module under different energy availability. When the harvested energy exceeds the load demand, the system works in continuous mode, delivering a stable DC output while simultaneously charging the battery with pulsed current (Fig. 5B(i), with Paths 1 and 2 on and Path 3 off). Conversely, when the harvested energy is insufficient, the hybrid sustained mode is activated. In this mode, the DC–DC converter and the backup battery alternately supply power to the load with seamless transitions, ensuring stable operation (Fig. 5B(ii), with Paths 1 and 3 alternately switched on and off, and Path 2 always off). Furthermore, when the battery is unavailable (e.g., depleted) and the harvested energy is too low to support the continuous operation of the load, the intermittent mode is triggered. The module stores energy over time and periodically releases it as a regulated DC output (Fig. 5B (iii), with Path 1 alternately switched on and off, while Paths 2 and 3 are always off). In intermittent mode, the energy delivered to the load per triggering depends on the storage capacitance (${C}_{{{{\rm{S}}}}}$) and UVLO voltage thresholds (${C}_{{{{\rm{TH}}}},{{{\rm{high}}}}}$ and ${C}_{{{{\rm{TH}}}},{{{\rm{low}}}}}$), as given by:

4 $${E}_{{{{\rm{deliver}}}}}=\frac{1}{2}{C}_{S}\left({V}_{{{{\rm{TH}}}},{{{\rm{high}}}}}^{2}-{V}_{{{{\rm{TH}}}},{{{\rm{lower}}}}}^{2}\right)$$

The total regulated output power, ${P}_{{{{\rm{reg}}}}}$, delivered through the load supply path (Path 1) and the battery charging path (Path 2), serves as the metric for evaluating the overall performance of the power management system, as shown below:

5 $${P}_{{{{\rm{reg}}}}}={V}_{{{{\rm{reg}}}}}\times ({I}_{{{{\rm{load}}}}}+{I}_{{{{\rm{bat}}}}})$$

where ${V}_{{{{\rm{reg}}}}}$ is the stabilized output voltage, ${I}_{{{{\rm{load}}}}}$ is the current through the load supply path, and ${I}_{{{{\rm{bat}}}}}$ is the current through the battery charging path, measured using a Device Current Waveform Analyzer (see Methods section and Supplementary Fig. 50). The total output power under different load resistances is measured and summarized in Fig. 5C, alongside the direct AC output under the same condition for comparison. Additionally, the system’s output performance across various generator rotation speeds is illustrated in Fig. 5D. Supplementary Figs. 52 and 53 present the detailed waveforms and the separated power in the load and charging paths. The battery backup supply (Path 3) is disabled during testing to ensure a consistent comparison of output power with and without power management. The results demonstrate that the proposed power management strategy achieves excellent performance, yielding comparable or even higher output power than that achieved under the optimal matched-load condition. Most importantly, the energy regulation module enables the automatic distribution of harvested energy between the load and the backup battery based on the energy availability and load demand, ensuring high energy utilization efficiency. Estimates indicate that 66.7% of the power in the battery charging path (Path 2) is successfully delivered to the battery (Supplementary Fig. 54), while the remainder is lost due to the inevitable voltage mismatch between the circuit output and the battery voltage. Compared with configurations without UVLO such as a standalone buck converter or a buck converter with DC–DC conversion, the proposed power management system demonstrates superior performance, especially at lower load resistances (Fig. 5E).

The high performance of the proposed power management system allows a 55-mm-radius generator to reliably drive diverse electronic arrays and complex systems, even at a rotational speed as low as 30 rpm, as shown in Fig. 5F–H. In the demonstrations, the output voltage of the power management system is regulated according to the operating voltage range of the target electronics. As demonstrated in Fig. 5F and Supplementary Movie 1, the generator can continuously and reliably power 40 hygrothermographs connected in parallel at a rotation speed of 30 rpm. Notably, while driving these devices, ${V}_{{{{\rm{CS}}}}}$ slightly increases before being stabilized by the Zener diode, indicating that power generation exceeds operational demand. Note that the battery charging and backup supply paths (Paths 2 and 3) are disabled in this demonstration. Similarly, Fig. 5G and Supplementary Movie 2 demonstrate the continuous operation of seven wireless IoT sensors powered by the generator at 30 rpm, with excess energy available beyond the sensors’ requirements. After 300 s of operation, residual energy stored in ${C}_{{{{\rm{S}}}}}$ sustains sensor functionality for over 150 s without battery support. Figure 5H and Supplementary Movie 3 illustrate simultaneous battery charging and device operation. At 36 rpm, the generator produces just enough energy to continuously power a high-power LoRa sensing node that measures five parameters and transmits at 20 dBm every 30 s. At 60 rpm, surplus energy becomes available beyond the node’s power requirements, enabling backup battery charging. At 300 rpm, the surplus energy increases, accelerating the charging process and raising the voltage of a 250 mAh Li-ion battery from 3.15 V to 3.60 V within 2.5 h (Supplementary Fig. 55). After the generator stops, the stored energy sustains LoRa node operation for over 13.5 h, demonstrating efficient energy storage in the battery. Notably, the LoRa node is a dynamically varying load, whose supply current can surge from 2 mA to 66.5 mA within 1 ms during transmission (Supplementary Fig. 56). Even under this condition, the output of our management system remains stable, demonstrating robust performance. We further tested our power management system with both capacitive loads (Supplementary Fig. 57) and inductive loads (Supplementary Fig. 58), confirming its ability to maintain stable operation under different load conditions. These applications demonstrate the versatility of the proposed power management system.

## Discussion

This study proposes a self-powered and universal power management strategy for triboelectric and electrostatic generators, consisting of a self-powered peak-detection switch for efficient energy extraction, a low-loss passive LC buck converter for efficient energy conversion, and an integrated energy regulation module for efficient energy utilization. Experimental validation using hardware-emulated and rotational electret generators confirms the system’s adaptability to diverse operating frequencies, output voltages, and physical configurations. The managed DC output achieves power comparable to that of the optimal AC output while maintaining a stable, low-voltage output suitable for conventional electronics. Moreover, the power management system dynamically routes the surplus energy to battery storage when the harvested energy is abundant and activates backup power when the harvested energy is insufficient, improving energy utilization efficiency while ensuring uninterrupted power supply even under intermittent harvesting conditions. This work bridges the gap between laboratory prototypes and practical implementations, enabling triboelectric and electrostatic generators to function as reliable power sources for real-world applications.

Looking ahead, the proposed power management system can be further miniaturized and integrated, broadening its applicability to portable, wearable, and other space-constrained devices. Achieving this goal requires overcoming three main challenges. First, miniaturizing the peak-detection switch is challenging because reduced component and trace spacing increases parasitic capacitance, which can compromise switching reliability. This necessitates careful circuit layout and shielding. Second, the LC buck converter remains a major size bottleneck, with the inductor dominating the footprint. Techniques such as multiple-release energy extraction^49^, in which energy delivery is divided into smaller packets per cycle, could help reduce inductor size and enable greater integration. Third, the energy regulation module is still relatively large; integrating it into an application-specific integrated circuit (ASIC) could substantially shrink its footprint. This, however, would demand suitable high-voltage processes and optimized DC–DC conversion to handle the elevated input voltage while preserving efficiency. Addressing these challenges will be critical to advancing the current prototype into a compact, fully integrated solution for widespread deployment.

## Methods

### Fabrication of the rotational electret generator

The electret generator’s rotor consists of a 30-μm-thick bipolar-charged polytetrafluoroethylene (PTFE) film attached to a 1.6-mm-thick FR-4 substrate using a 100 μm-thick polyimide (PI) double-sided adhesive tape. The bipolar charge pattern on the PTFE film was prepared by a patterned contact micro-discharge method adapted from our previous work^50^. Briefly, the PTFE film was sandwiched between a pair of sector-patterned charging electrodes fabricated on a printed circuit board (PCB) and covered with a solder-resist layer, which improved charge uniformity and suppressed dielectric breakdown during charging. When a sufficiently high DC bias was applied, microscopic air gaps at the electrode/PTFE interfaces generated localized micro-discharges, enabling charge injection and trapping on the PTFE surface. The bipolar charge pattern was formed by two successive charging steps under opposite polarities, with the PTFE film rotated by one sector pitch relative to the patterned electrodes between the two steps. The stator was fabricated via a standard PCB process. The dimensional parameters of all generator configurations are listed in Supplementary Table 5.

### Measurement

The electret generator was driven by a motor, with its rotation speed controlled by a custom-built proportional-integral-derivative (PID) controller. The voltages were recorded using Keysight oscilloscopes EXR054A and DSOX2024A, with the latter used for the demonstration experiments. Open-circuit and direct AC output voltages of the generators were measured using a homemade analog front-end (1 GΩ input impedance, < 0.3 pF input capacitance). Other voltage measurements were carried out with a Keysight 10076C probe (66.7 MΩ || 3 pF, 4 kVpk) and a Keysight N2842A probe (10 MΩ || 11 pF, 400 Vpk). The current was measured using a Device Current Waveform Analyzer (Keysight CX3322A) with a Keysight CX1101A probe (410 mΩ, 40nA – 1A). Capacitance values were determined using a Victor 4092C LCR meter, and resistance values were measured with an Agilent 34411A 6½-digit multimeter.

## Supplementary information

Supplementary Information

Description of Additional Supplementary Files

Supplementary Movie 1

Supplementary Movie 2

Supplementary Movie 3

Transparent Peer Review file

## Source data

Source data

## Data Availability

All data needed to evaluate the conclusions in the paper are present in the paper and/or the Supplementary Materials. Additional inquiries regarding the data can be directed to the corresponding author. Source data are provided with this paper.

## References

[1] Zhu, J. et al. More than energy harvesting in electret electronics-moving toward next-generation functional system. *Adv. Funct. Mater.* **33**, 2214859 (2023).

[2] Zeadally, S., Shaikh, F. K., Talpur, A. & Sheng, Q. Z. Design architectures for energy harvesting in the Internet of Things. *Renew. Sustain. Energy Rev.* **128**, 109901 (2020).

[3] Zhao, X., Askari, H. & Chen, J. Nanogenerators for smart cities in the era of 5G and Internet of Things. *Joule* **5**, 1391–1431 (2021).

[4] Niu, S. & Wang, Z. L. Theoretical systems of triboelectric nanogenerators. *Nano Energy* **14**, 161–192 (2015).

[5] Sabzpoushan, S. & Woias, P. Electret-based energy harvesters: a review. *Nano Energy* **131**, 110167 (2024).

[6] Zi, Y. et al. Harvesting low-frequency ( < 5 Hz) irregular mechanical energy: a possible killer application of triboelectric nanogenerator. *ACS Nano* **10**, 4797–4805 (2016). 27077467 10.1021/acsnano.6b01569

[7] Cao, Z. et al. Ultrahigh power generation from low-frequency and micro motion by suppressing air breakdown. *Energy Environ. Sci.* **17**, 5002–5012 (2024).

[8] Wang, Z. et al. Ultrahigh electricity generation from low-frequency mechanical energy by efficient energy management. *Joule* **5**, 441–455 (2021).

[9] Wu, H., Wang, S., Wang, Z. & Zi, Y. Achieving ultrahigh instantaneous power density of 10 MW/m^2^ by leveraging the opposite-charge-enhanced transistor-like triboelectric nanogenerator (OCT-TENG). *Nat. Commun.* **12**, 5470 (2021). 34526498 10.1038/s41467-021-25753-7 PMC8443631

[10] Li, Y. et al. Advanced dielectric materials for triboelectric nanogenerators: principles, methods, and applications. *Adv. Mater.* **36**, 2314380 (2024). 10.1002/adma.202314380 38517171

[11] Li, X. et al. Polymer electrets and their applications. *J. Appl. Polym. Sci.* **138**, 50406 (2021).

[12] Wang, A. C., Wu, C., Pisignano, D., Wang, Z. L. & Persano, L. Polymer nanogenerators: opportunities and challenges for large-scale applications. *J. Appl. Polym. Sci.* **135**, 45674 (2018).

[13] Hasan, M. A. M., Zhu, W., Bowen, C. R., Wang, Z. L. & Yang, Y. Triboelectric nanogenerators for wind energy harvesting. *Nat. Rev. Electr. Eng.* **1**, 453–465 (2024).

[14] Hasan, M. A. M., Zhang, T., Wu, H. & Yang, Y. Water droplet-based nanogenerators. *Adv. Energy Mater.* **12**, 2201383 (2022).

[15] Xu, W. et al. A droplet-based electricity generator with high instantaneous power density. *Nature* **578**, 392–396 (2020). 32025037 10.1038/s41586-020-1985-6

[16] Wang, X. et al. Mechanical vibration energy harvesting and vibration monitoring based on triboelectric nanogenerators. *Energy Technol.* **12**, 2300931 (2024).

[17] Zhang, C. et al. Active resonance triboelectric nanogenerator for harvesting omnidirectional water-wave energy. *Joule* **5**, 1613–1623 (2021).

[18] Mei, X., Miyoshi, T. & Suzuki, Y. A dynamic model of wrist-worn rotational electret energy harvester: Theoretical investigation and experimental validations. *Appl. Energy* **373**, 123888 (2024).

[19] Zhang, H., Lu, Y., Ghaffarinejad, A. & Basset, P. Progressive contact-separate triboelectric nanogenerator based on conductive polyurethane foam regulated with a Bennet doubler conditioning circuit. *Nano Energy* **51**, 10–18 (2018).

[20] Ghaffarinejad, A., Yavand Hasani, J., Galayko, D. & Basset, P. Superior performance of half-wave to full-wave rectifier as a power conditioning circuit for triboelectric nanogenerators: application to contact-separation and sliding mode TENG. *Nano Energy* **66**, 104137 (2019).

[21] Zhu, G., Chen, J., Zhang, T., Jing, Q. & Wang, Z. L. Radial-arrayed rotary electrification for high performance triboelectric generator. *Nat. Commun.* **5**, 3426 (2014). 24594501 10.1038/ncomms4426

[22] Zhou, H. et al. Recent progress of switching power management for triboelectric nanogenerators. *Sensors* **22**, 1668 (2022). 35214570 10.3390/s22041668 PMC8880102

[23] Zi, Y. et al. An inductor-free auto-power-management design built-in triboelectric nanogenerators. *Nano Energy* **31**, 302–310 (2017).

[24] Liu, W. et al. Switched-capacitor-convertors based on fractal design for output power management of triboelectric nanogenerator. *Nat. Commun.* **11**, 1883 (2020). 32312950 10.1038/s41467-020-15373-y PMC7171113

[25] Liu, Y., Badel, A. & Suzuki, Y. Enhancing output power of rotational electret energy harvester by synchronized switch harvesting on inductor. *J. Intell. Mater. Syst. Struct.* **33**, 183–195 (2022).

[26] Wang, Z. et al. A universal self-triggered passive management strategy for enhancing the output power of triboelectric nanogenerators. *Energy Environ. Sci.* **18**, 3761–3772 (2025).

[27] Liu, Y., Shi, Z., Badel, A., Miyoshi, T. & Suzuki, Y. Self-powered synchronous electric charge extraction rectifier for rotational electret energy harvester with dual-stage electrodes. *IEEE Trans. Power Electron* **38**, 13166–13180 (2023).

[28] Wang, Z. et al. Giant performance improvement of triboelectric nanogenerator systems achieved by matched inductor design. *Energy Environ. Sci.* **14**, 6627–6637 (2021).

[29] Zi, Y. et al. Standards and figure-of-merits for quantifying the performance of triboelectric nanogenerators. *Nat. Commun.* **6**, 8376 (2015). 26406279 10.1038/ncomms9376 PMC4598564

[30] Gao, Y. et al. Achieving high-efficiency triboelectric nanogenerators by suppressing the electrostatic breakdown effect. *Energy Environ. Sci.* **16**, 2304–2315 (2023).

[31] Zhou, H. et al. Autonomous cantilever buck switch for ultra-efficient power management of triboelectric nanogenerator. *Appl. Energy* **357**, 122475 (2024).

[32] Shang, W. et al. Rotational pulsed triboelectric nanogenerators integrated with synchronously triggered mechanical switches for high efficiency self-powered systems. *Nano Energy* **82**, 105725 (2021).

[33] Kim, J. Y., Kaganovich, I. & Lee, H.-C. Review of the gas breakdown physics and nanomaterial-based ionization gas sensors and their applications. *Plasma Sources Sci. Technol.* **31**, 033001 (2022).

[34] Zhang, H. et al. Employing a MEMS plasma switch for conditioning high-voltage kinetic energy harvesters. *Nat. Commun.* **11**, 3221 (2020). 32591516 10.1038/s41467-020-17019-5 PMC7319968

[35] Wu, Z. et al. Electrostatic generator enhancements for powering IoT nodes via efficient energy management. *Microsyst. Nanoeng.* **10**, 30 (2024). 38455381 10.1038/s41378-024-00660-1 PMC10918071

[36] Harmon, W., Bamgboje, D., Guo, H., Hu, T. & Wang, Z. L. Self-driven power management system for triboelectric nanogenerators. *Nano Energy* **71**, 104642 (2020).

[37] Zheng, R. et al. Self-switching circuit of TENG for energy storage and power management in harvesting wind energy. *J. Intell. Mater. Syst. Struct.* **35**, 197–205 (2024).

[38] Cheng, X. et al. High efficiency power management and charge boosting strategy for a triboelectric nanogenerator. *Nano Energy* **38**, 438–446 (2017).

[39] Liufu, Y. H., Dai, D. & Liu, Z. Triple-MOSFETs switch for adaptive maximum capacitance point tracking of triboelectric nanogenerators. *Nano Energy* **106**, 108042 (2023).

[40] Chang, S. et al. Harvesting high entropy triboelectric energy using a universal synchronous switching unit for self-powered wireless sensing systems. *Nano Energy* **131**, 110271 (2024).

[41] Wu, H. et al. Efficient energy conversion mechanism and energy storage strategy for triboelectric nanogenerators. *Nat. Commun.* **15**, 6558 (2024). 39095412 10.1038/s41467-024-50978-7 PMC11297214

[42] Liu, X. et al. An ultrasound-driven implantable wireless energy harvesting system using a triboelectric transducer. *Matter* **5**, 4315–4331 (2022).

[43] Yang, H. et al. Triboelectric nanogenerator integrated with a simple controlled switch for regularized water wave energy harvesting. *Nano Res* **17**, 7585–7592 (2024).

[44] Luo, Y. et al. Advanced energy harvesting from low-frequency ocean waves for lithium-ion battery applications. *Energy Environ. Sci.* **18**, 4821–4832 (2025).

[45] Dai, Y. et al. Effective charging of commercial lithium cell by triboelectric nanogenerator with ultrahigh voltage energy management. *Adv. Sci.* **11**, 2404253 (2024). 10.1002/advs.202404253 PMC11321660 38864316

[46] Wang, J. et al. A cross regulation reduced multi-output and multi-VCR piezoelectric energy harvesting system using shared capacitors. in *2022**IEEE International Symposium on Circuits and Systems (ISCAS)* 2768–2772 (IEEE, 2022).

[47] Hosseini, A., Badeli, A. S., Davari, M., Sheikhaei, S. & Gharehpetian, G. B. A novel, software-defined control method using sparsely activated microcontroller for low-power, multiple-input, single-inductor, multiple-output DC–DC converters to increase efficiency. *IEEE Trans. Ind. Electron.* **70**, 2959–2970 (2023).

[48] Li, S., Liu, X. & Calhoun, B. H. A 32 nA fully autonomous multi-input single-inductor multi-output energy-harvesting and power-management platform with 1.2 × 10^5^ dynamic range, integrated MPPT, and multi-modal cold start-up. in *2022**IEEE International Solid- State Circuits Conference (ISSCC)* 1–3 (IEEE, 2022).

[49] Tu, Y. et al. IC-compatible high-efficiency power management for triboelectric nanogenerators based on the concept of limit. *IEEE Trans. Power Electron* **39**, 6–13 (2024).

[50] Wu, Z., Bi, M., Cao, Z., Wang, S. & Ye, X. Largely enhanced electrostatic generator based on a bipolar electret charged by patterned contact micro-discharge and optimized substrates. *Nano Energy* **71**, 104602 (2020).

