# Supplementary material for: Power management for triboelectric and electrostatic generators enabling continuous and stable power delivery
Source: Nat Commun. 2026 Apr 25;17:5704. doi: 10.1038/s41467-026-72323-w (PMC13320162; doi:10.1038/s41467-026-72323-w)
Supplement: Supplementary file 1 — Supplementary Information [file 41467_2026_72323_MOESM1_ESM.pdf]

Supplementary Information

Power management for triboelectric and electrostatic generators enabling continuous and stable power delivery

Zeyuan Cao*, Junchi Teng, Yongjia Zhao, Xingqi Guo, Jiani Xu, Chao Ren, and Xiongying Ye*

State Key Laboratory of Precision Measurement Technology and Instruments, Department of Precision Instrument, Tsinghua University, Beijing 100084, China.

* Corresponding authors:
E-mail: caozy@mail.tsinghua.edu.cn (Z. Cao), xyye@mail.tsinghua.edu.cn (X. Ye).

This file includes:
Supplementary Tables 1–5
Supplementary Figures 1–63
Supplementary Notes 1–4
Supplementary References 1–24

Supplementary Table 1. Comparison of power management performance with previous works.

No.	References	Generator type	Power management strategy	Direct output (P_{AC})	Final regulated output (P_{reg})	End devices	Power ratio (P_{reg}/P_{AC})
1	Energy & Eenvironmental Science 18, 3761–3772 (2025)[1]	Contact-separation TENG	SCR-based voltage-triggered switch with LC Buck and hysteresis voltage control	3.1 mW @ 100 MΩ	0.65 mW (V_{out} varies from 1.7-2.8 V)	Resistive load	0.21
2	Nature Communications 15, 6558 (2024)[2]	Rotating TENG	Discharging switch with LC Buck and DC-DC converter	17.31 mW @ 60 MΩ	6.9 mW @ 1.8 V	Resistive load	0.40
3	Advanced Science 11, 2404253 (2024)[3]	Contact-separation TENG	SCR-based voltage-triggered switch with LC buck converter for charging the battery	2.32 mW @ 60 MΩ	1.3-1.56 mW ($V_{charge,bat}$ = 2.7-4.41 V)	Li-ion Battery	0.56-0.67
4	Energy & Eenvironmental Science 18, 4821–4832 (2025)[4]	Rotating TENG with gearbox	Charging battery via matched inductive transformer	66.14 mW @ 110 kΩ	42-60 mW ($V_{charge,bat}$ = 2.8-4 V)	Li-ion Battery	0.63-0.91
5	This work	Rotating electret generator	Self-powered peak-detection switch with LC buck and self-adaptive energy regulation	13.04 mW @ 10 MΩ	14.2 mW @ 5 V	Resistive load and Li-ion battery	**1.08**

Note:

The performance metric of the power management system is defined as the ratio P_{reg} / P_{AC}, where P_{reg} is the regulated power ultimately delivered to the electronic loads and P_{AC} is the generator's maximum AC output at optimal load. These metric captures both the energy enhancement and the conversion efficiency of the power management system.

Supplementary Table 2. Comparison of power management performance with previous works.

References	Energy Source	Energy Optimization Strategy	Switch Type	Switch Architecture	Self-Powered/Cold Start	Static Power Consumption	Tolerated input voltage	Operation Frequency	Convertor Topology	Energy Regulation	Performance metric #1 $P_{reg}^{**}/P_{AC,opt}^{**}$	Performance metric #2 P_{reg}/P_{CMEO}^{**}	Other performance metric
Nano Energy (2017)[5]	Triboelectric (In-plane)	CMEO*	Peak	Discrete controller + low-side N-MOSFET	No	2.69 μW	<450 V	N/A	Flyback	No	N/A	N/A	$P_{unreg}^{**}/P_{AC,opt}^{**}$ 74.45% @ 4.47 V
Nano Energy (2019)[6]	Triboelectric (In-plane)	CMEO	Peak	Discrete controller + low-side N-MOSFET	No	2.69 μW	<305 V	N/A	LC buck	No	N/A	N/A	$P_{unreg}/P_{AC,opt}$ = 69.3% @ 1.7 V
IEEE Transactions on Power Electronics (2023)[7]	Electret (In-plane)	CMEO	Peak	Discrete controller + low-side N-MOSFET	Yes (Delayed start)	1~2 μW	<250 V	~300 Hz	LC buck	No	N/A	N/A	$P_{unreg}/P_{FBR+Buck}^{**}$ = 2.1 @ 4.6 V
Nano Energy (2023)[8]	Triboelectric (Out-of-plane)	CMEO with matching capacitor	Peak	Triple MOSFET Structure	Yes (Immediate start)	0	<300 V	1~3 Hz	LC buck	No	N/A	N/A	P_{unreg}/P_{in}^{*}= 79.3 % @ 6.2 V
Nano Energy (2024)[9]	Triboelectric (Out-of-plane)	CMEO with matching capacitor	Peak	BJT-based peak-tracking	Yes (Immediate start)	0	<200V	N/A	LC buck	No	N/A	N/A	$P_{sw}/P_{AC,opt}$ = 88.2% @ C_{in}=220 pF
IEEE Transactions on Circuits and Systems I[10]	Triboelectric (Out-of-plane)	CMEO	Peak	0.18 μm CMOS IC controller + low-side N-MOSFET	Yes (Delayed start)	1.56 μW	<300 V	1~5 Hz	Flyback	No	N/A	N/A	P_{unreg}/P_{CEMO} = 51.2% @ 7.56 V
ISSCC (2018)[11]	Triboelectric (Out-of-plane)	FBR*+MPPT*	Threshold	Embedded switch in 0.18 μm BCD* IC	Yes (Delayed start)	N/A	70 V	N/A	LC buck	No	N/A	N/A	P_{unreg}/P_{in} = 51.1 % @ 4.7 V
2019 Symposium on VLSI Circuits (2019)[12]	Triboelectric (N/A)	FBR+MPPT	Threshold	Embedded switch in 0.18 μm BCD IC	Yes (Delayed start)	361.6 nW	70 V	N/A	Dual input buck	N/A	N/A	N/A	P_{unreg}/P_{in} = 70.72% @ P_{in} = 20.7 μW
Joule (2019)[13]	Triboelectric (Out-of-plane)	CMEO	Threshold	Air-discharge switch	Yes (Immediate start)	0	7.5 kV	1~3 Hz	Flyback	No		N/A	$P_{unreg}/P_{AC,opt}$ 78.5% @ 15 V
Advanced Science (2024)[3]	Triboelectric (Out-of-plane)	CMEO with matching capacitor	Threshold	SCR-based switch	Yes (Immediate start)	0	1790 V (fixed)	N/A	LC buck	Battery (2.7-4.41 V)	0.56-0.67*(1) @ 2.7-4.4 V	N/A	N/A
Nature Communications (2024)[2]	Triboelectric (In-plane)	CMEO	Threshold	Needle-discharge switch	Yes (Immediate start)	0	~2500 V (fixed)	N/A	LC buck	DC-DC (1.8 V)	0.40*(2) @ 1.8 V	N/A	N/A
Energy Environ. Sci. (2025)[14]	Triboelectric (Out-of-plane)	CMEO with matching capacitor	Threshold	SCR-based switch	Yes (Immediate start)	0	N/A	N/A	LC buck	Hysteresis regulation (1.7-2.8 V)	0.21*(3) @ 1.7-2.8 V	N/A	N/A
Energy Environ. Sci. (2025)[4]	Triboelectric (In-plane)	N/A	N/A	N/A	N/A	N/A	N/A	~ 1364 Hz	Inductive transformer	Battery (2.8-4 V)	0.63-0.91*(4) @ 2.8-4 V	N/A	$P_{unreg}/P_{AC,opt}$ = 83.74% @ 3.21 V

IEEE Transactions on Power Electronics (2024)[15]	Triboelectric (Out-of-plane)	MR-SCE*	Peak	Discrete controller + MOSFETs	No	N/A	142 V	2 Hz	Switch capacitor convertor	No	N/A	N/A	P_{unreg}/P_{CMEO} 22.3 % @ 18 V
IEEE Journal of Solid-State Circuits (2022)[16]	Triboelectric (Out-of-plane)	SSHI*	Zero-Cross Detection	Embedded switch in 0.18 µm BCD IC	Yes (Delayed start)	N/A	<195 V	N/A	Switch Capacitor convertor	Battery (2.7-4.2 V)	N/A	0.38*(5) @ 3.6 V	P_{reg}/P_{in} = 70.7 % @ 3.6 V
IEEE Transactions on Industrial Electronics (2023)[17]	Triboelectric (Out-of-plane)	SSHI	Peak	0.18 µm BCD IC controller + low-side N-MOSFET	Yes (Delayed start)	N/A	<65 V	200~400 Hz	Switch Capacitor convertor	Battery (2.7-4.2 V)	N/A	0.32*(6) @ 3.6 V	P_{reg}/P_{in} = 79.3% @ 3.6 V
Journal of Intelligent Material Systems and Structures (2022)[18]	Electret (In-plane)	SSHI	Peak	Discrete controller with low-side MOSFET	Yes (Delayed start)	6 µW	<240 V	~109 Hz	N/A	No	N/A	N/A	$P_{unreg}/P_{FBR,\ opt}$ = 1.22 @ 3 V
Electronics Letters (2018)[19]	Triboelectric (Out-of-plane)	Bennet doubler	N/A	N/A	N/A	N/A	N/A	N/A	N/A	No	N/A	N/A	$P_{unreg}/P_{FBR,\ opt}$ 152.3 @ 256 V
Nano Energy (2018)[20]	Triboelectric (Out-of-plane)	Bennet doubler	N/A	N/A	N/A	N/A	N/A	N/A	N/A	No	N/A	N/A	$P_{unreg}/P_{FBR,\ opt}$ 2.5 @ 380 V
IEEE Transactions on Industrial Electronics (2023)[21]	Thermoelectric Photovoltaic Piezoelectric	MPPT	N/A	N/A	N/A	N/A	2.2-3.2 V 2.6-3.8 V 3-4.2 V	N/A	Discrete MISIMO* converter 7-19;0-1.8; 2.2-4.2 V		N/A	N/A	P_{reg}/P_{in} = 92.5%
ISSCC (2022)[22]	Thermoelectric Photovoltaic Piezoelectric	MPPT SECE*	N/A	N/A	Yes (Delayed start)	N/A	<5 V	N/A	65nm CMOS MISIMO converter 0.6/1.2/3.3 V		N/A	N/A	P_{reg}/P_{in} = 80%
ISCAS (2022)[23]	Piezoelectric	SSHI	Zero-cross Detection	N/A	N/A	N/A	V_{PEH} = 1.7 V	250 Hz	0.18µm CMOS Switch capacitor DC-DC converter 0.5/1/2 V		N/A	N/A	P_{reg}/P_{in} = 0.894 (Simulation)
This work	Electret (In-plane)	CMEO	Peak	Positive-feedback-based discrete switch	Yes (Immediate start)	0	<1200 V	12 ~ 120 Hz	LC buck	DC–DC + Automatic Power Allocation (1.5/2.7/5 V)	1.08 @ 5 V	0.45	$P_{unreg}/P_{AC,opt}$ = 1.48 @401 V; $P_{unreg}/P_{FBR,opt}$** =2.4; P_{unreg}/P_{CMEO} =0.6; P_{reg}/P_{unreg} = 93.4% @ V_{unreg} = 12-18 V; P_{reg}/P_{sw}** = 79.3%; $P_{reg}/P_{FBR,opt}$ =1.78

*: CMEO (cycle for maximized energy output); FBR (Full-wave bridge rectifier); MPPT (Maximum power point tracking); MR-SCE (multi-release synchronized charge extraction); SSHI (synchronized switch harvesting on inductor); SECE (synchronous electric charge extraction); BCD (bipolar-CMOS-DMOS); MISIMO (multi-input single-inductor multi-output).

**: Power definitions: P_{reg} (average power with regulated DC output); $P_{AC,opt}$ (optimal average power with AC output); P_{unreg} (maximum average power with unregulated DC output); $P_{FBR+buck}$ (average power with full-wave bridge rectifier and buck converter); P_{in} (input power to the power management system); $P_{FBR,\ opt}$ (optimal output power under full bridge rectification); P_{sw} (average power output with peak switch); P_{CMEO} (output power of generator operating in CMEO. For a generator with time-independent capacitance and symmetrical output, P_{CMEO} satisfies the relation $P_{CMEO} = 4\ P_{FBR,opt}$); $P_{chg,bat}$ (average battery-charging power).

*(#) Estimated values: (1) $P_{chg,bat}$ = 1.3 mW @ 2.7 V, $P_{chg,bat}$ = 1.56 mW @ 4.4V, $P_{AC,opt}$ = 2.32 mW; (2) P_{reg} = 6.9 mW @ 1.8 V, $P_{AC,opt}$ = 17.31 mW; (3) P_{reg} = 0.67 mW, $P_{AC,opt}$ = 3.1 mW; (4) $P_{chg,bat}$ = 42 mW @ 2.8 V, $P_{chg,bat}$ = 60 mW @ 2.8 V, $P_{AC,opt}$ = 66.14 mW; (5) P_{in} = 823.3 µW, P_{CMEO} = 4*$P_{FBR,opt}$ = 1540.8 µW, P_{reg} / P_{CMEO} = P_{reg} / P_{in} * P_{in}/P_{CMEO} = 70.7% * 823.3 / 1540.8;(6) V_{OC} = 150V, V_R = 60V, β = 1, P_{reg} / P_{CMEO} = P_{reg} / P_{in} * P_{in} / P_{CMEO} = 79.3% * 60 / 150.

Supplementary Table 3. Default parameters for testing the self-powered peak-detection switch.

**Targets**	**Parameters**	**Value**
Test platform (Hardware-emulated generator)	Generator capacitance C_{EG}	120 pF
	Generator voltage V_{EG}	1200 V
	Voltage drop rate dV/dt	20 V ms^{-1}
Self-powered Peak-detection switch	C_{in}	10 pF
	C_{drive}	120 pF
	R_{NMOS}	3.9 MΩ
	D_1, D_2	HD20G
	D_3, D_4	STTH112A
	P-MOSFET	DMP10H4D2S-7
	N-MOSFET	IXTY02N120P*

* The drain-to-source parasitic capacitance ($C_{DS,NMOS}$) of the IXTY02N120P is 6.7 pF, as specified in its datasheet.

Supplementary Table 4. Parameters for loss analysis of LC buck converter.

**Targets**	**Parameters**	**Key values**	**Note**
Test platform (Hardware-emulated generator)	Generator capacitance C_{EG}	47 pF	-
	Generator voltage V_{EG}	1000 V	-
	Voltage drop rate	20 V ms^{-1}	-
Self-powered peak-detection switch	C_{in}	10 pF	-
	C_{drive}	120 pF	-
	R_{NMOS}	3.9 MΩ	-
	D_1, D_2	HD20G	-
	D_3, D_4	STTH112A	-
	P-MOSFET	DMP10H4D2S-7	-
	N-MOSFET	IXTY02N120P*	-
Diodes (D_S)	D_{S1}	V_F = 37.5V@10mA<br>V_R = 12000V	2CL73
	D_{S2}	V_F = 4V@0.2A<br>V_R = 3000V	R3000
	D_{S3}	V_F = 1.7V@2A<br>V_R = 1000V	HER208G
	D_{S4} (default)	V_F = 1.9V@1A<br>V_R = 1200V	STTH112A
Inductor (L_S)	L_{S1}	7.54mH & 4.98 Ω*<br>I_{sat} = 35 mA	07M-682K-50
	L_{S2}	2.72mH & 3.48 Ω*<br>I_{sat} = 470mA	11PHC-272K-YY
	L_{S3}	4.74mH & 3.4 Ω*<br>I_{sat} = 380 mA	RF1317-475KL
	L_{S4} (default)	6.74 mH & 6.9 Ω*<br>I_{sat} = 470mA	Two series 11PHC-272K-YY (series-aiding connection)
Capacitor (C_S)	C_{S1}	0.998 μF*	Rated:1 μF, Film capacitor
	C_{S2} (default)	10.32 μF*	Rated:10 μF, Film capacitor
	C_{S3}	8.83 μF*	Rated:10 μF, Electrolytic capacitor
	C_{S4}	93.4 μF*	Rated:100 μF Electrolytic capacitor

* The value is measured by the Victor 4092C LCR meter.

Supplementary Table 5. Parameters of the electret generators with different physical configurations.

Device number	Inner radius, *r* (mm)	Outer radius, *R* (mm)	Electrode pairs, *n*	Generator capacitance (pF)
#1	10	30	16	31.2
#2	10	30	24	35.7
#3	10	40	16	52.9
#4	10	40	24	60.8
#5	10	55	16	79.3
#6	10	55	24	101.5

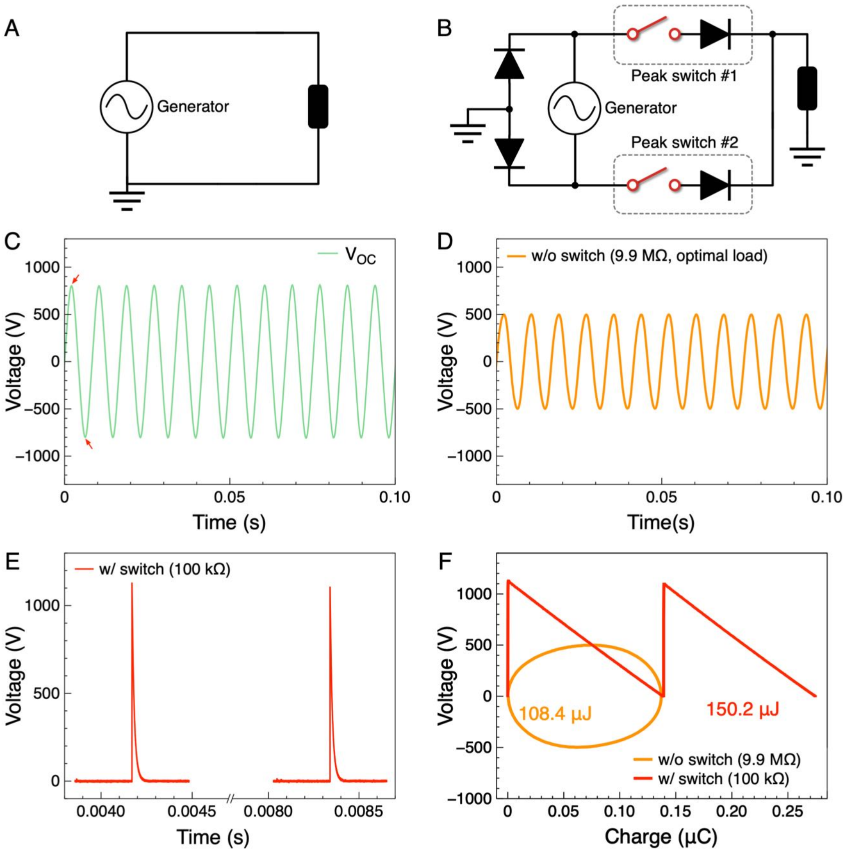

Supplementary Figure 1. Demonstration of the energy-enhancement effect enabled by the synchronous switch. (A) Schematic of the generator with a resistive load (direct AC output). (B) Schematic of the generator with the bipolar peak-detection switch topology. (C) Open-circuit voltage of the generator, with red arrows indicating the closure times of the peak switch. (D) Direct AC output of the generator under the optimal load resistance. (E) Output of the generator with the synchronous switches. (F) V–Q plots and the corresponding output energy per cycle, with and without the switch.

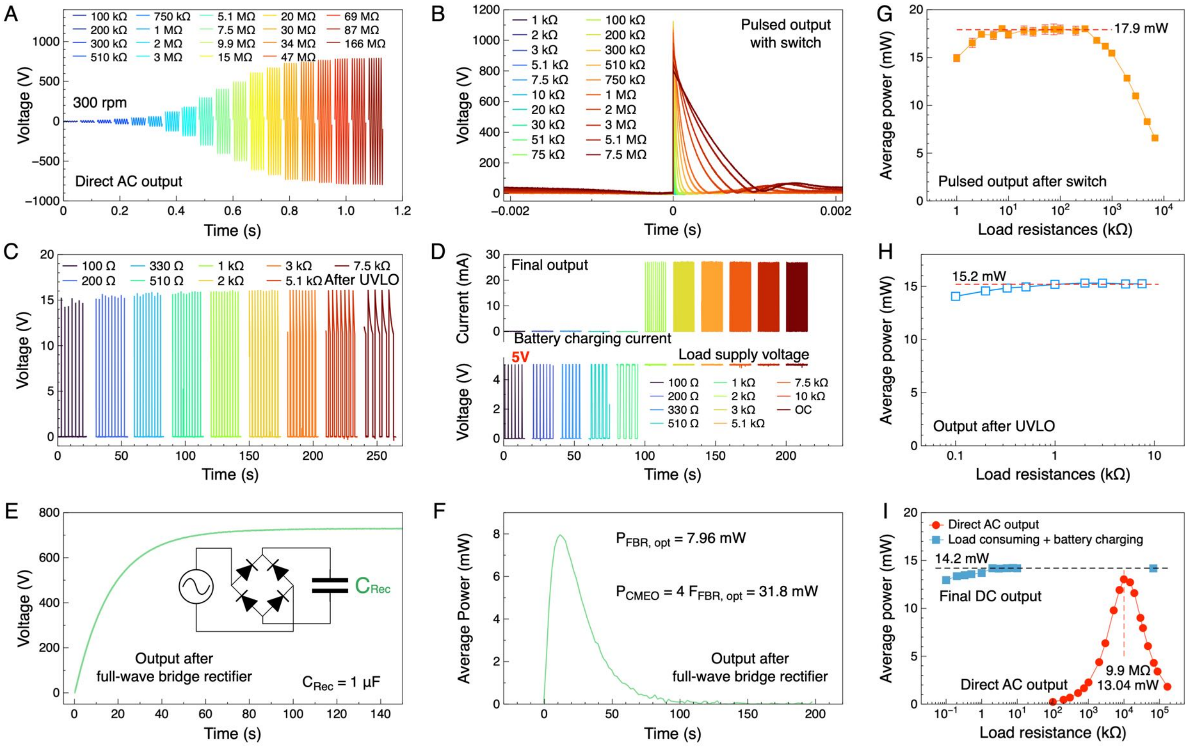

Supplementary Figure 2. Waveforms of (A) the direct AC output voltage from the generator, (B) the pulsed output voltage after the switch, (C) the voltage output after the UVLO, (D) the regulated DC output and battery charging current after the energy regulation module, and (E) the rectified voltage after full-wave bridge rectifier. (F–I) their corresponding average output power. The average power shown in (G) was the mean value obtained from $n = 5$ independent experiments. Error bars represent s.d. The output power at CMEO condition (P_{CMEO}) is estimated through the relation $P_{\mathrm{CMEO}} = 4P_{\mathrm{FBR,opt}}$,[10] which holds when the generator output is symmetric.

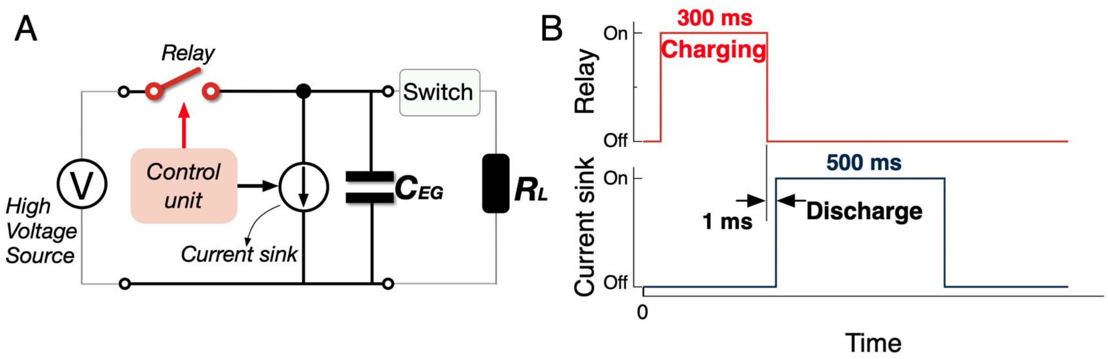

Supplementary Figure 3. (A) Schematic of the test platform. (B) Timing sequence of control signals for the relay and the current sink.

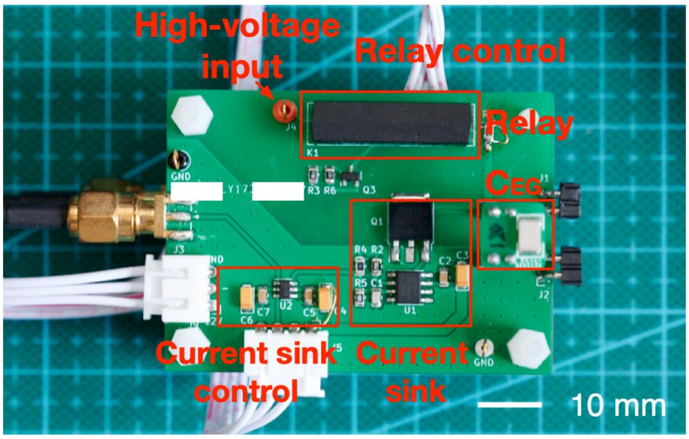

Supplementary Figure 4. Photograph of the test platform.

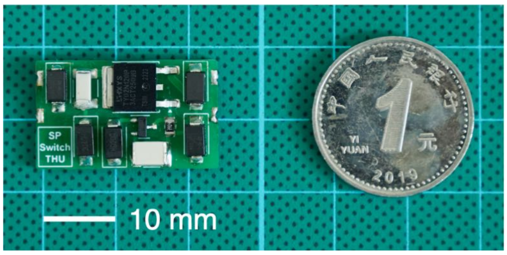

Supplementary Figure 5. Photograph of the self-powered peak-detection switch.

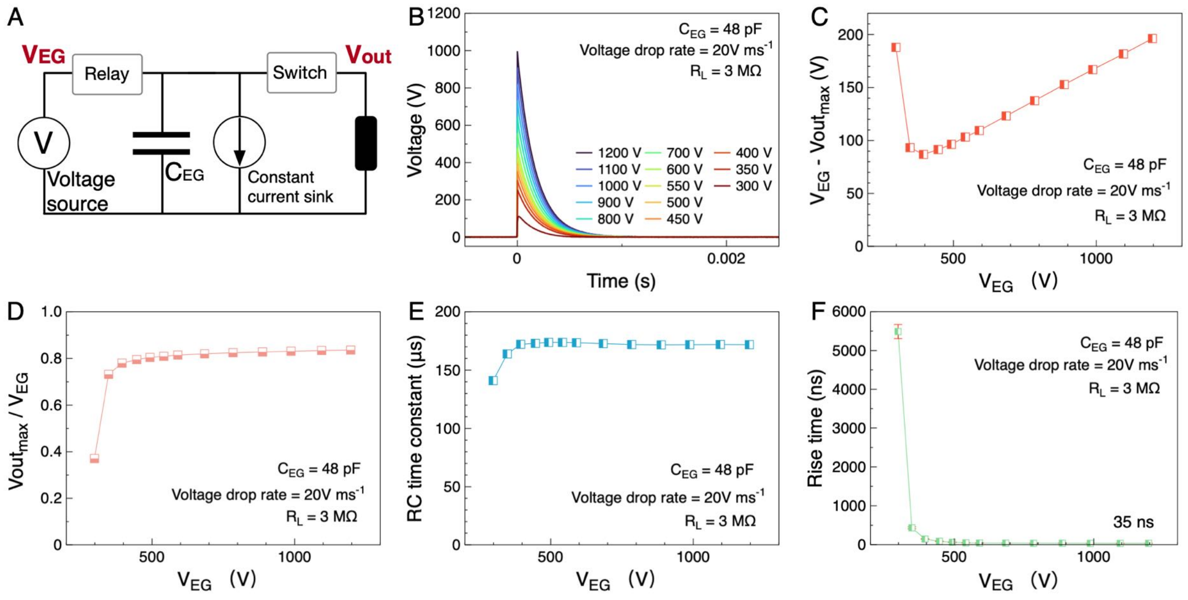

Supplementary Figure 6. Performance of the self-powered peak-detection switch at different generator voltages (C_{EG} = 48 pF). (A) Schematic of the test circuit. (B) Output voltages across the load. (C) Absolute voltages drop after switch closure.(D) Relative voltages drop after switch closure.(E) RC time constants of the output pulses.(F) Rise time of the output voltage, presented as the mean ± s.d. from n = 5 independent experiments.

Note:

1) To facilitate comparison of switch performance across different C_{EG} values, the time constant was adjusted to 100–200 µs by adjusting R_{L} in the tests of Supplementary Figures 6–15.

2) According to the analysis in Figure 2A, the absolute voltage drop needs to trigger the switch is determined by the turn-on threshold of the P-MOSFET ($V_{\mathrm{GS,P}}$) and should maintain the same. However, in practice, the voltage drop after switch closure can be larger due to the parasitic capacitance at the load side ($C_{\mathrm{par}} = C_{\mathrm{wire}} + C_{\mathrm{probe}}$). C_{par} can lead to an additional voltage drop ($V_{\mathrm{drop,add}} = V_{\mathrm{EG}} \times C_{\mathrm{par}} / (C_{\mathrm{EG}} + C_{\mathrm{par}})$), which is especially pronounced with smaller generator capacitance, as evidenced by the comparison across Supplementary Figures 6C–20C.

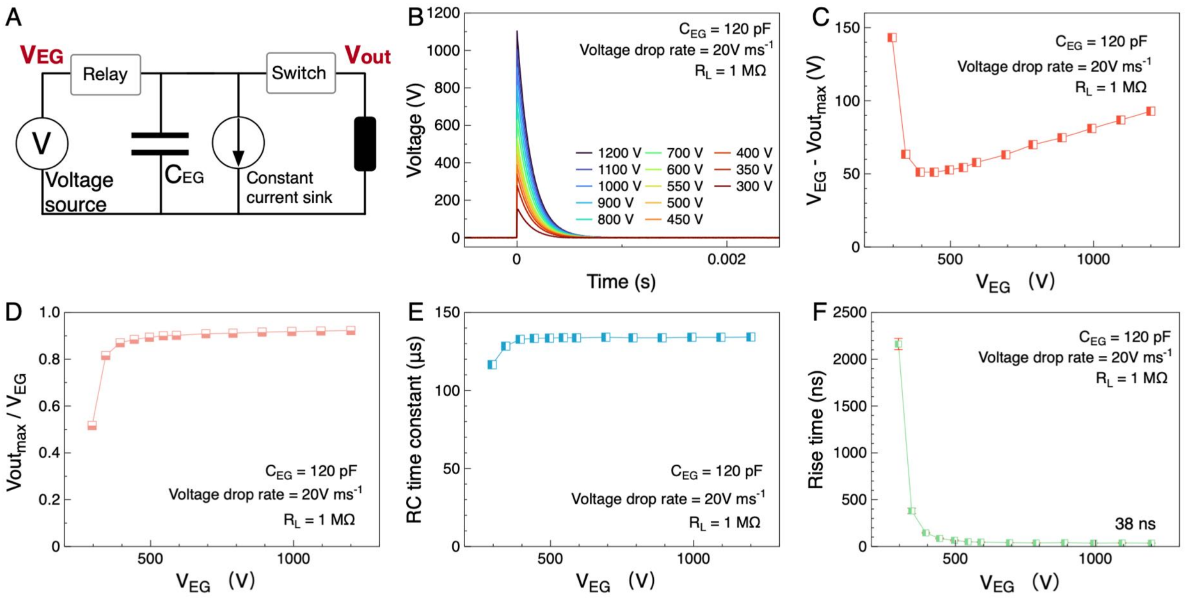

Supplementary Figure 7. Performance of the self-powered peak-detection switch at different generator voltages (C_{EG} = 120 pF). (A) Schematic of the test circuit. (B) Output voltages across the load. (C) Absolute voltages drop after switch closure. (D) Relative voltages drop after switch closure. (E) RC time constants of the output pulses. (F) Rise time of the output voltage, presented as the mean ± s.d. from n = 5 independent experiments.

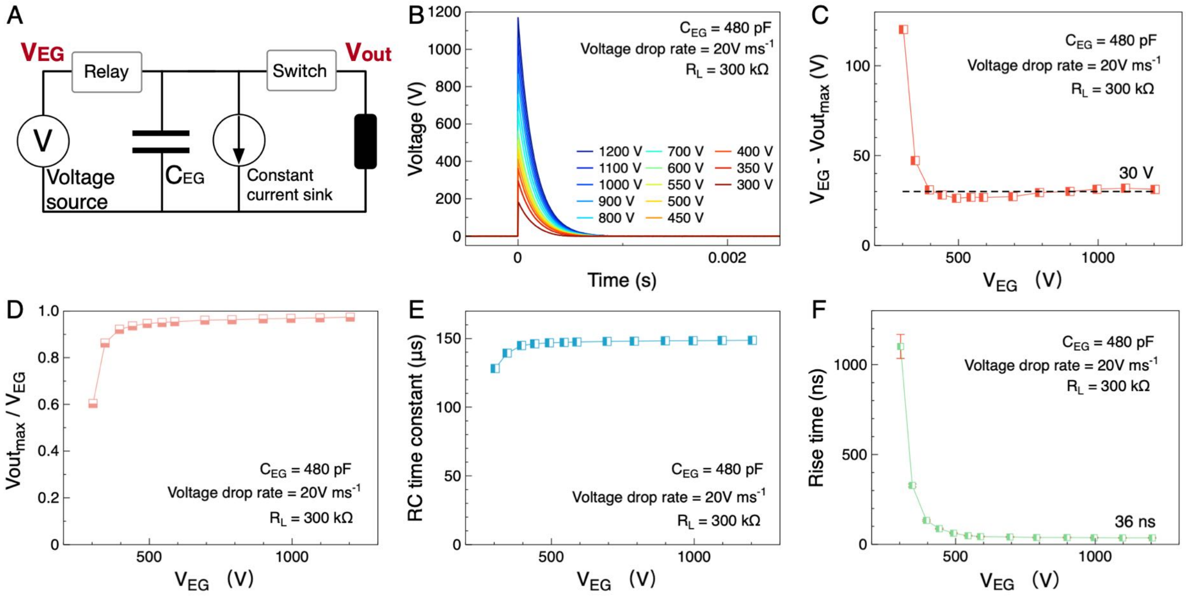

Supplementary Figure 8. Performance of the self-powered peak-detection switch at different generator voltages (C_{EG} = 480 pF). (A) Schematic of the test circuit. (B) Output voltages across the load. (C) Absolute voltages drop after switch closure. (D) Relative voltages drop after switch closure. (E) RC time constants of the output pulses. (F) Rise time of the output voltage, presented as the mean ± s.d. from n = 5 independent experiments.

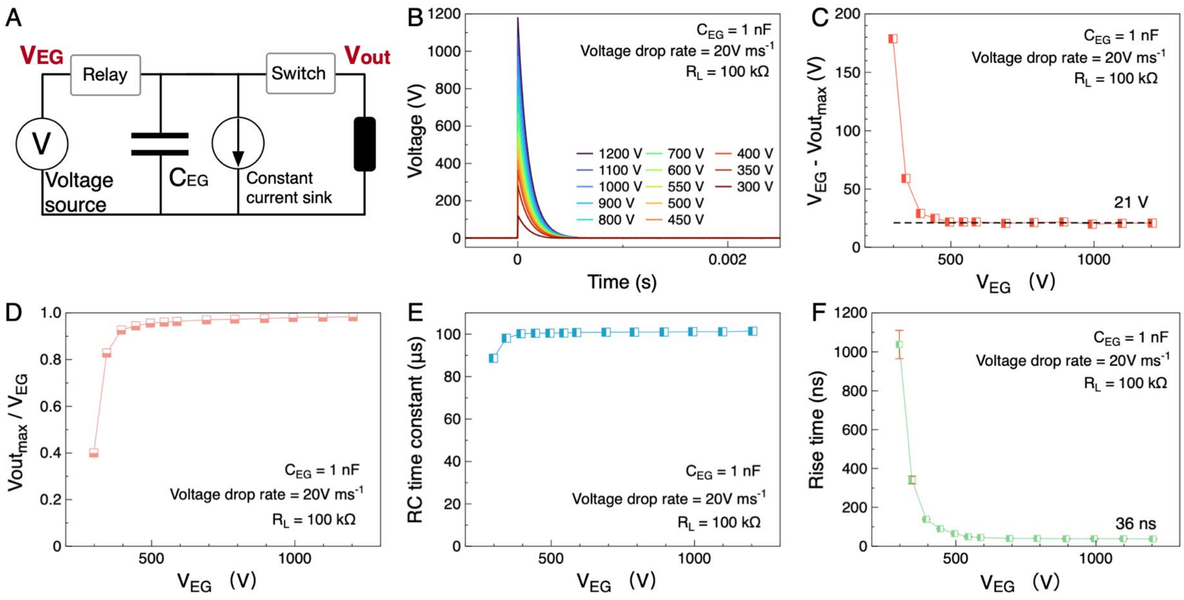

Supplementary Figure 9. Performance of the self-powered peak-detection switch at different generator voltages (C_{EG} = 1 nF). (A) Schematic of the test circuit. (B) Output voltages across the load. (C) Absolute voltages drop after switch closure. (D) Relative voltages drop after switch closure. (E) RC time constants of the output pulses. (F) Rise time of the output voltage, presented as the mean ± s.d. from n = 5 independent experiments.

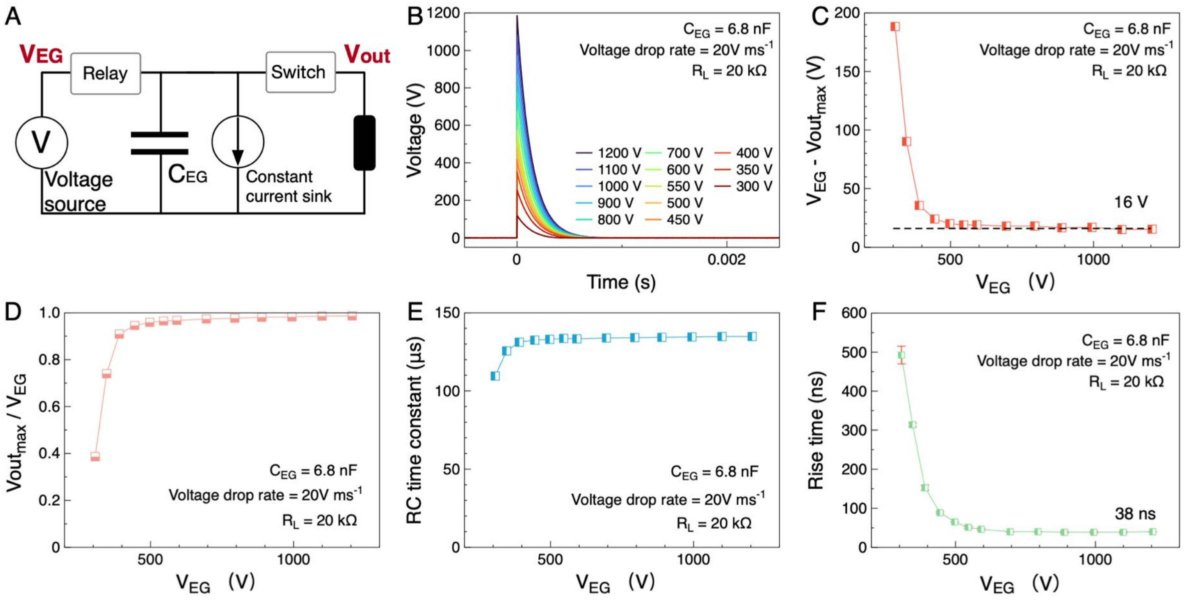

Supplementary Figure 10. Performance of the self-powered peak-detection switch at different generator voltages (C_{EG} = 6.8 nF). (A) Schematic of the test circuit. (B) Output voltages across the load. (C) Absolute voltages drop after switch closure. (D) Relative voltages drop after switch closure. (E) RC time constants of the output pulses. (F) Rise time of the output voltage, presented as the mean ± s.d. from n = 5 independent experiments.

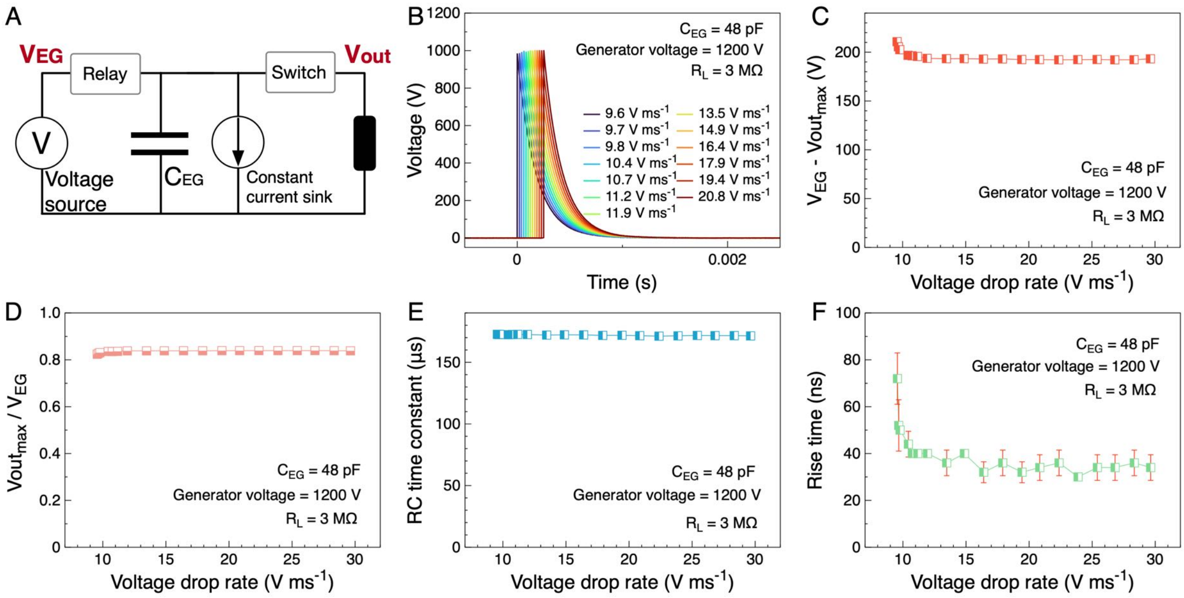

Supplementary Figure 11. Performance of the self-powered peak-detection switch at different voltage drop rates (C_{EG} = 48 pF). (A) Schematic of the test circuit. (B) Output voltages across the load, where waveforms are time-shifted for clarity. (C) Absolute voltages drop after switch closure. (D) Relative voltages drop after switch closure. (E) RC time constants of the output pulses. (F) Rise time of the output voltage, presented as the mean ± s.d. from n = 5 independent experiments.

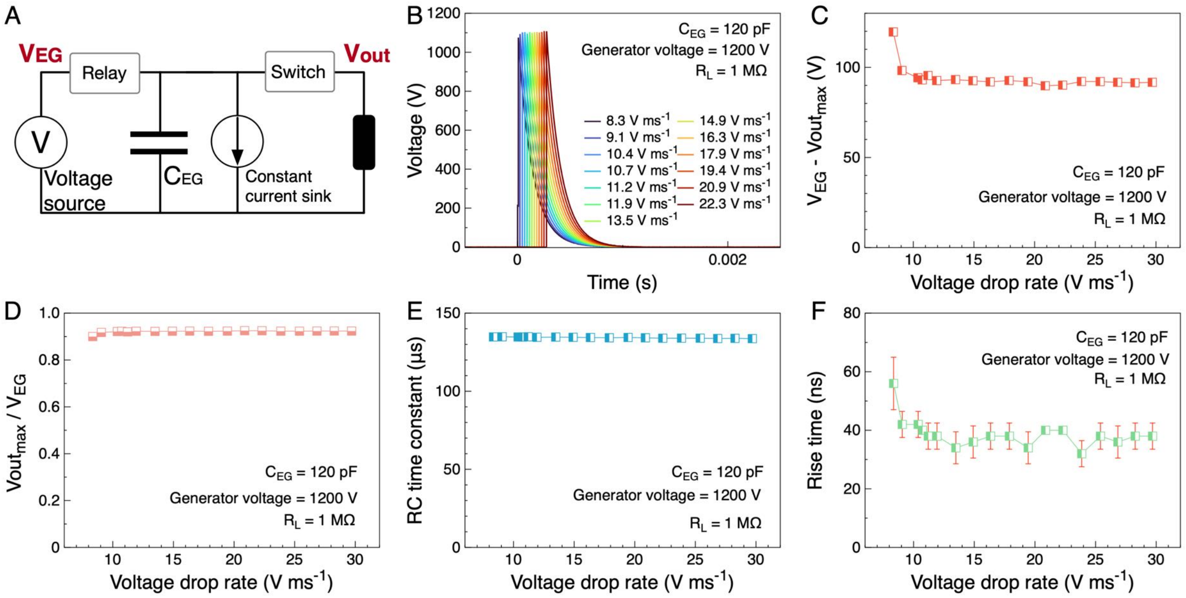

Supplementary Figure 12. Performance of the self-powered peak-detection switch at different voltage drop rates (C_{EG} = 120 pF).(A) Schematic of the test circuit. (B) Output voltages across the load, where waveforms are time-shifted for clarity. (C) Absolute voltages drop after switch closure. (D) Relative voltages drop after switch closure. (E) RC time constants of the output pulses. (F) Rise time of the output voltage, presented as the mean ± s.d. from $n = 5$ independent experiments.

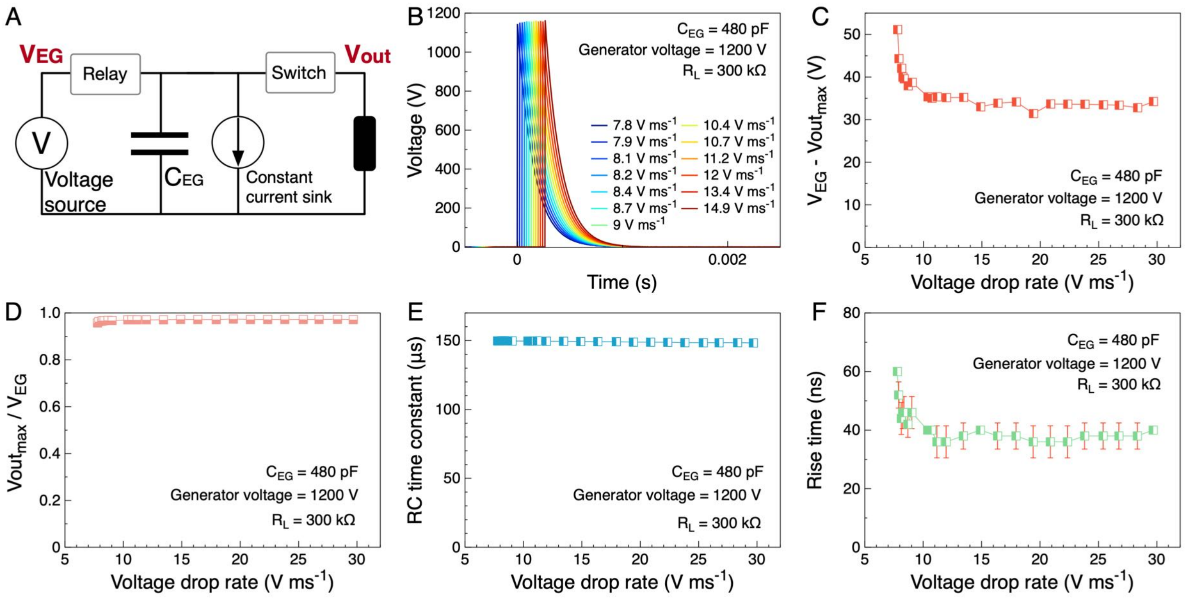

Supplementary Figure 13. Performance of the self-powered peak-detection switch at different voltage drop rates (C_{EG} = 480 pF). (A) Schematic of the test circuit. (B) Output voltages across the load, where waveforms are time-shifted for clarity. (C) Absolute voltages drop after switch closure. (D) Relative voltages drop after switch closure. (E) RC time constants of the output pulses. (F) Rise time of the output voltage, presented as the mean ± s.d. from n = 5 independent experiments.

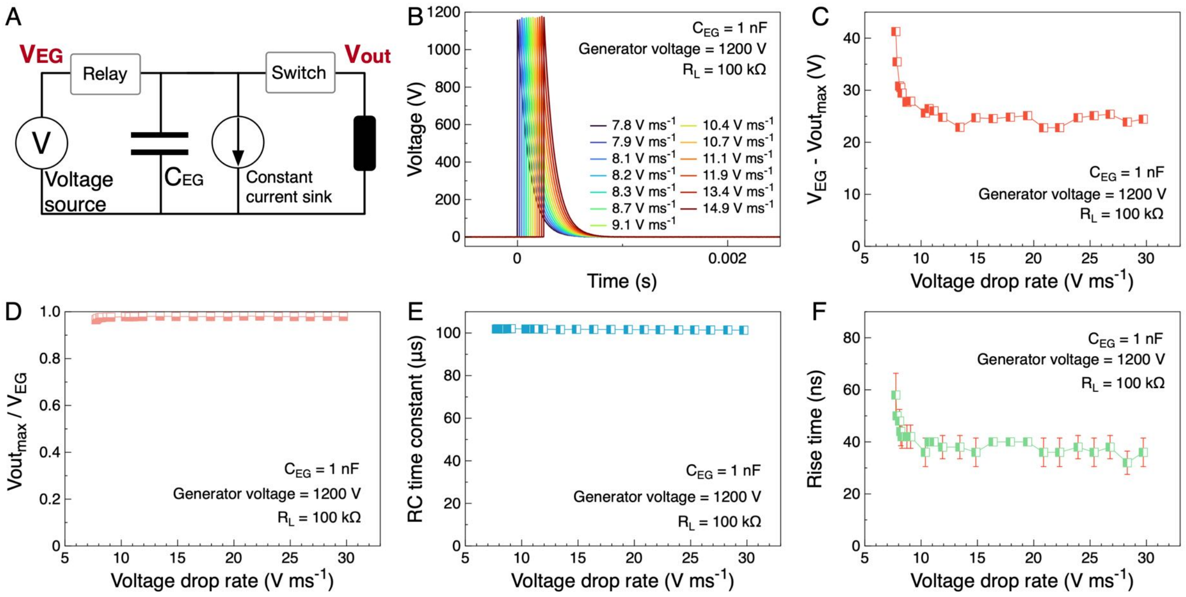

Supplementary Figure 14. Performance of the self-powered peak-detection switch at different voltage drop rates (C_{EG} = 1 nF). (A) Schematic of the test circuit. (B) Output voltages across the load, where waveforms are time-shifted for clarity. (C) Absolute voltages drop after switch closure. (D) Relative voltages drop after switch closure. (E) RC time constants of the output pulses. (F) Rise time of the output voltage, presented as the mean ± s.d. from n = 5 independent experiments.

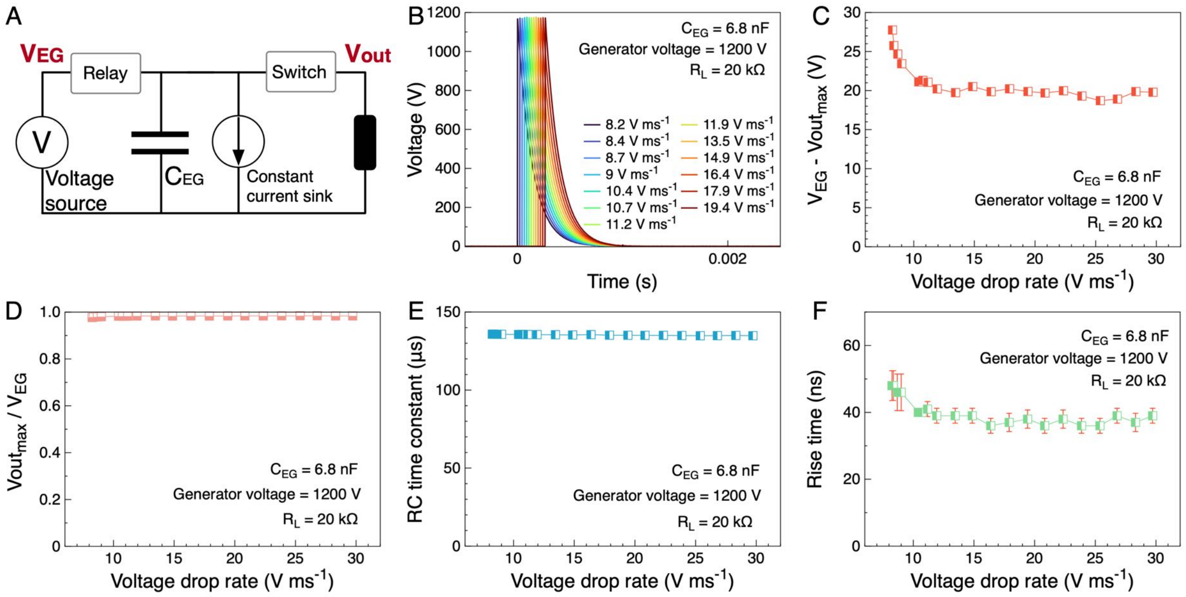

Supplementary Figure 15. Performance of the self-powered peak-detection switch at different voltage drop rates (C_{EG} = 6.8 nF). (A) Schematic of the test circuit. (B) Output voltages across the load, where waveforms are time-shifted for clarity. (C) Absolute voltages drop after switch closure. (D) Relative voltages drop after switch closure. (E) RC time constants of the output pulses. (F) Rise time of the output voltage, presented as the mean ± s.d. from n = 5 independent experiments.

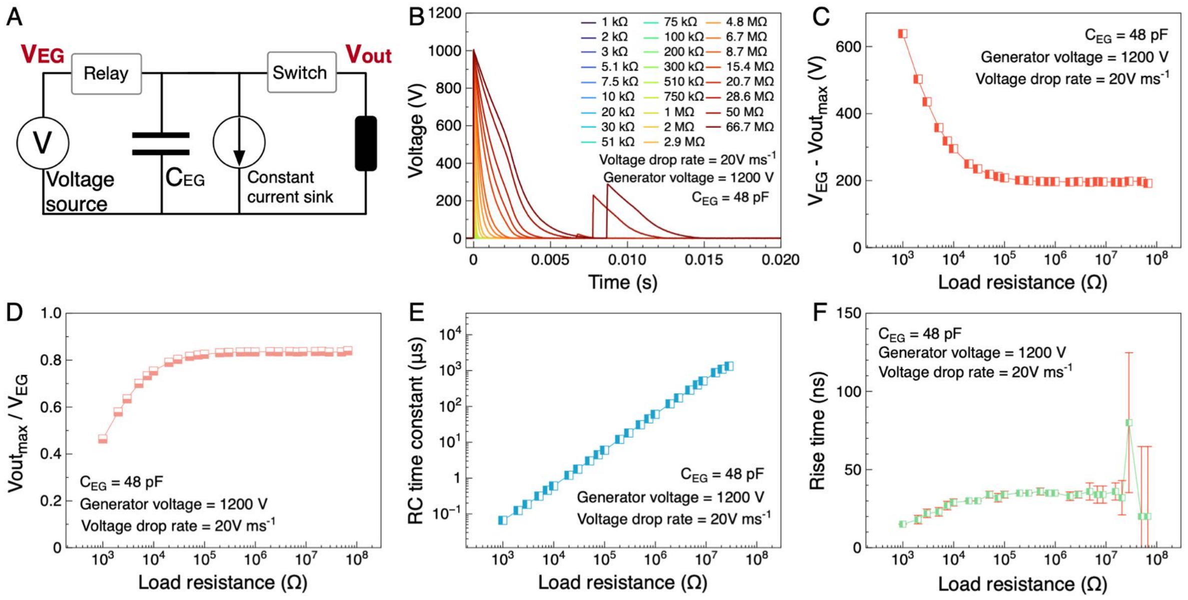

Supplementary Figure 16. Performance of the self-powered peak-detection switch at different load resistances (C_{EG} = 48 pF). (A) Schematic of the test circuit. (B) Output voltages across the load. (C) Absolute voltages drop after switch closure. (D) Relative voltages drop after switch closure. (E) RC time constants of the output pulses, calculated for waveforms with a single discharge pulse. (F) Rise time of the output voltage, presented as the mean ± s.d. from n = 5 independent experiments.

Note:

1) The multiple discharge pulses at higher load resistances in Supplementary Figures 16B–20B arise from incomplete discharge during a single switch closure caused by the mismatch between the large R_L-C_{EG} time constant and the insufficient switch closure time. This mismatch triggers multiple discharge events, as illustrated in Supplementary Figure 21.

2) The larger measured voltage drop at smaller load resistances in Supplementary Figures 16C–20C is attributed to the high on-resistance of the N-MOSFET (IXTY02N120P), specified as $R_{DS(on)} \leq 625\ \Omega$ in its datasheet.

3) The rise time variations with higher load resistances in Supplementary Figures 16F–20F are due to the reduced sampling rate at larger R_L values during measurement, which increases the calculation error.

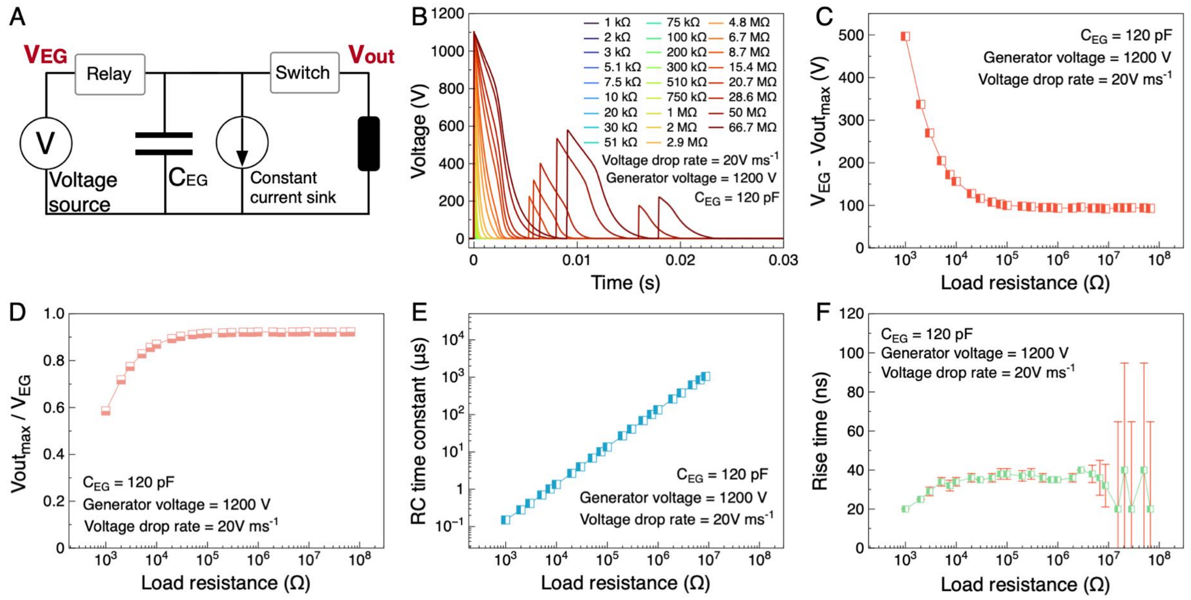

Supplementary Figure 17. Performance of the self-powered peak-detection switch at different load resistances (C_{EG} = 120 pF). (A) Schematic of the test circuit. (B) Output voltages across the load. (C) Absolute voltages drop after switch closure. (D) Relative voltages drop after switch closure. (E) RC time constants of the output pulses, calculated for waveforms with a single discharge pulse. (F) Rise time of the output voltage, presented as the mean ± s.d. from n = 5 independent experiments.

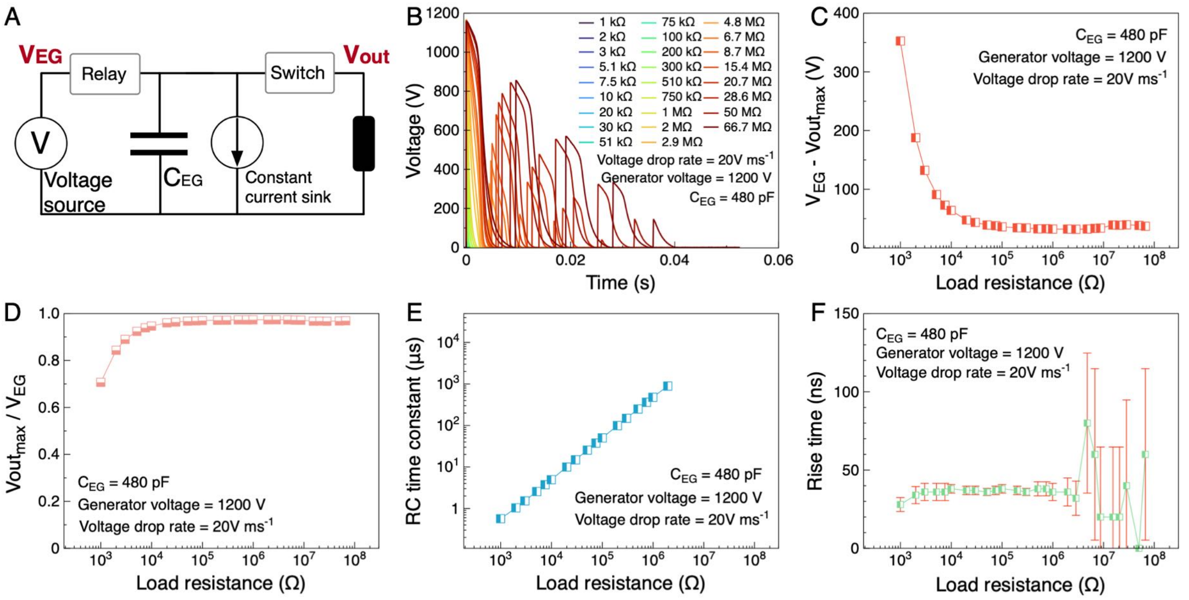

Supplementary Figure 18. Performance of the self-powered peak-detection switch at different load resistances (C_{EG} = 480 pF). (A) Schematic of the test circuit. (B) Output voltages across the load. (C) Absolute voltages drop after switch closure. (D) Relative voltages drop after switch closure. (E) RC time constants of the output pulses, calculated for waveforms with a single discharge pulse. (F) Rise time of the output voltage, presented as the mean ± s.d. from *n* = 5 independent experiments.

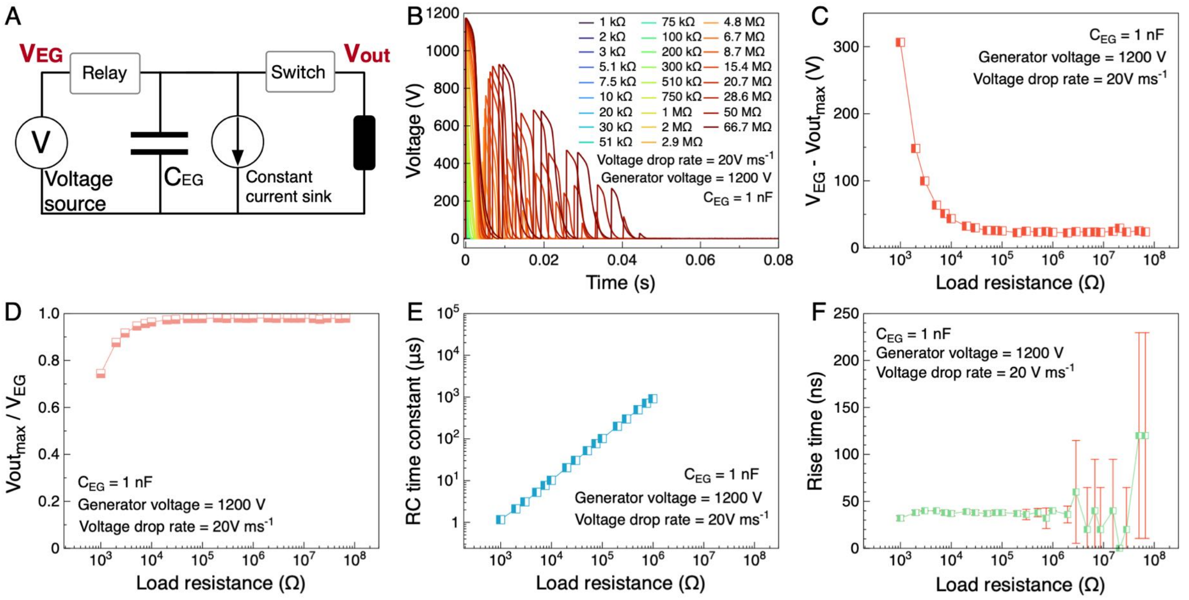

Supplementary Figure 19. Performance of the self-powered peak-detection switch at different load resistances (C_{EG} = 1 nF). (A) Schematic of the test circuit. (B) Output voltages across the load. (C) Absolute voltages drop after switch closure. (D) Relative voltages drop after switch closure. (E) RC time constants of the output pulses, calculated for waveforms with a single discharge pulse. (F) Rise time of the output voltage, presented as the mean ± s.d. from n = 5 independent experiments.

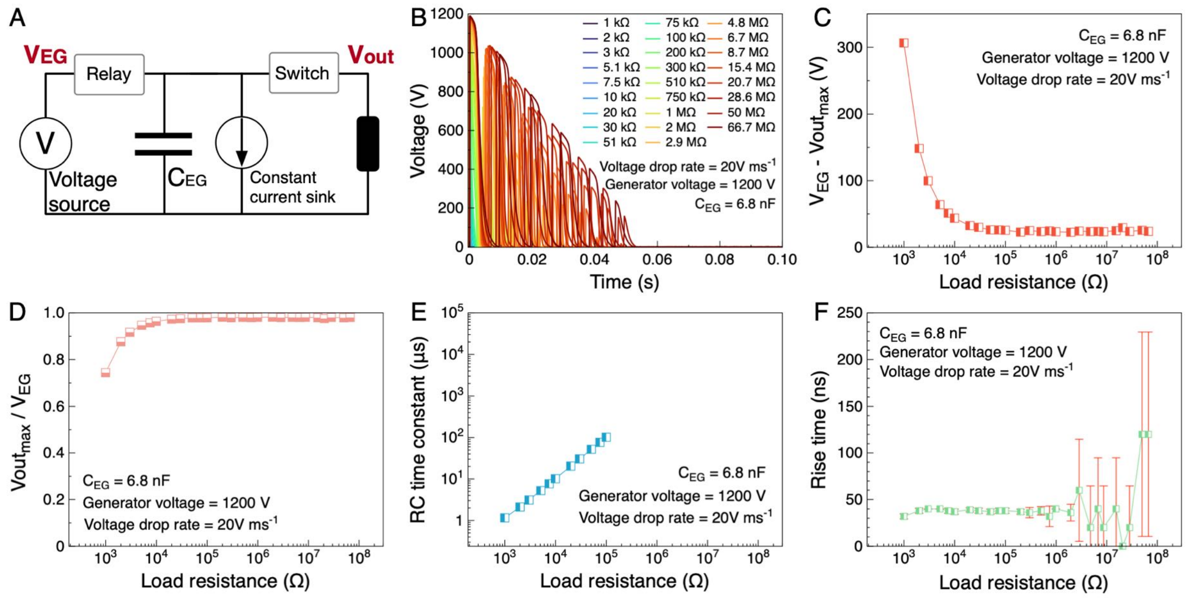

Supplementary Figure 20. Performance of the self-powered peak-detection switch at different load resistances (C_{EG} = 6.8 nF). (A) Schematic of the test circuit. (B) Output voltages across the load. (C) Absolute voltages drop after switch closure. (D) Relative voltages drop after switch closure. (E) RC time constants of the output pulses, calculated for waveforms with a single discharge pulse. (F) Rise time of the output voltage, presented as the mean ± s.d. from n = 5 independent experiments.

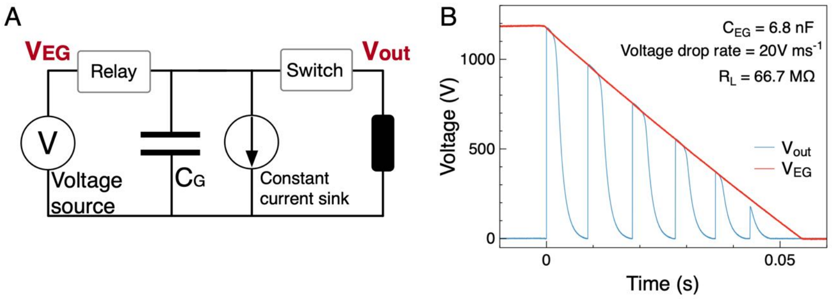

Supplementary Figure 21. (A) Schematic of the test circuit. (B) The switch undergoes multiple triggers due to a mismatch between the large R_L-C_{EG} RC time constant and the insufficient switch closure time. It continues to cycle on and off until V_{EG} decreases to zero.

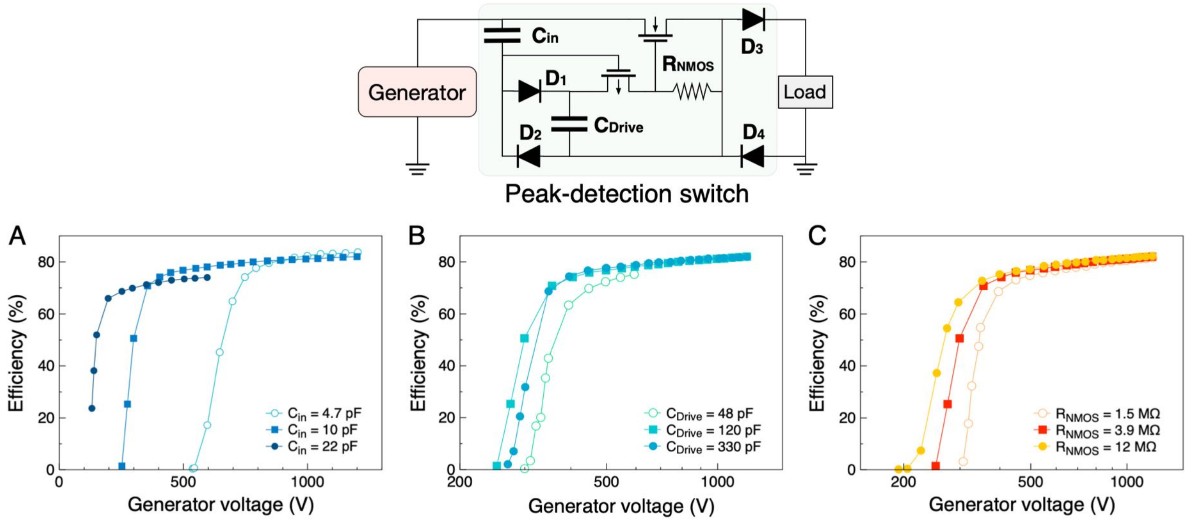

Supplementary Figure 22. Influence of (A) C_{in}, (B) C_{drive}, and (C) R_{NMOS} on switch efficiency with various generator voltages (Voltage drop rate = 20 V ms^{-1}, R_L = 3 MΩ, C_{EG} = 120 pF, unless otherwise noted).

Note:

Failure to trigger the switch occurs when the switch cannot get sufficient charge for activation. This happens when the generator voltage is relatively low, resulting in an insufficient charge ($Q_{drive} = V_{EG} \times C_{in}$) available in C_{drive} for driving the N-MOSFET.

1) To mitigate this issue, increasing C_{in} is an effective approach, as it enhances Q_{drive} and allows more charge to be maintained even at lower generator voltages, facilitating the switch conduction and lowering the switch's operating voltages, as shown in Supplementary Figure 22A. However, a larger C_{in} also increases the input capacitance of the switch, which may lead to higher driving consumption and reduced efficiency, particularly when the generator capacitance (C_{EG}) is comparable to C_{in}.

2) The value of C_{drive} does not affect Q_{drive}. However, it influences the driving voltage supplied to the N-MOSFET. An excessively large C_{drive} (330 pF here) results in insufficient voltage for driving the N-MOSFET, slightly raising the switch's minimum trigger voltage. Conversely, a C_{drive} that is too small (48 pF here) provides a higher drive voltage but increases its sensitivity to circuit leakage, which in turn reduces Q_{drive} and raises the switch's minimum trigger voltage. A moderate C_{drive} (120 pF here) with an appropriate driving voltage and sufficient capability to counteract leakage exhibits the best performance (Supplementary Figure 22B).

3) The value of R_{NMOS} influences the charge leakage during the establishment of positive feedback when triggering the switch. A larger R_{NMOS} allows more charge to drive the N-MOSFET, facilitating switch triggering and decreasing the switch's lowest triggerable voltage (Supplementary Figure 22C). However, since R_{NMOS} does not affect Q_{drive}, it has a smaller impact on the switch's lowest triggerable voltage than C_{in}.

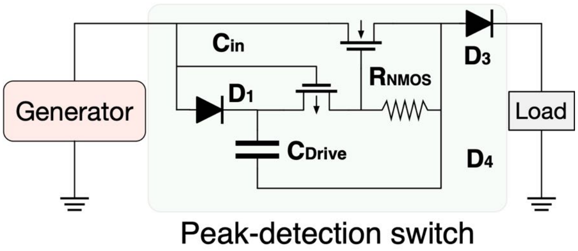

Supplementary Figure 23. Switch design for low-voltage generator applications.

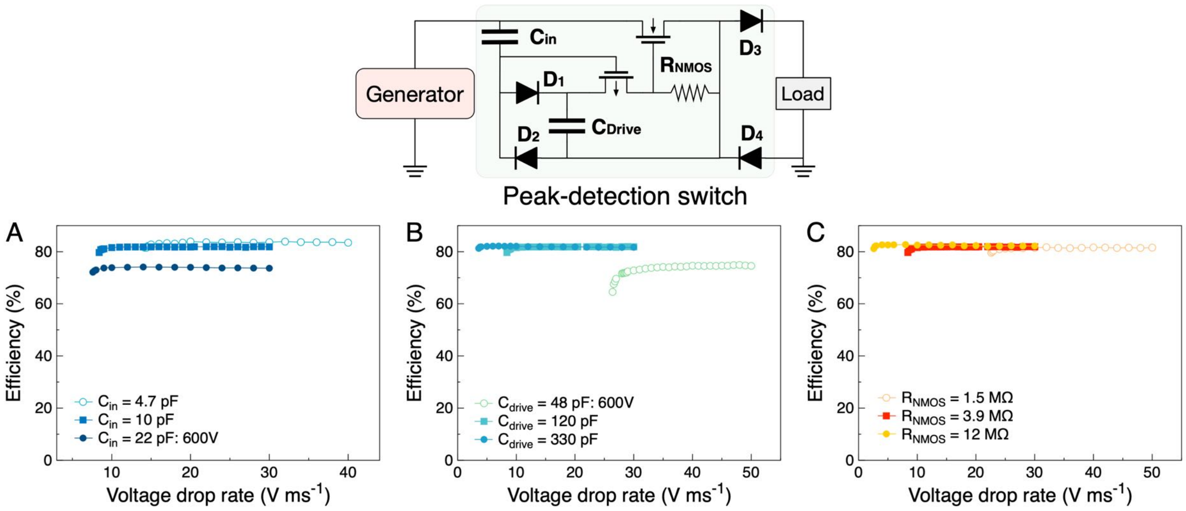

Supplementary Figure 24. Influence of (A) C_{in}, (B) C_{drive}, and (C) R_{NMOS} on switch efficiency with various voltage drop rates. (V_{EG} = 1200V, C_{EG} = 120 pF, R_{L} = 3 MΩ, unless otherwise noted)

Note:

Failure to trigger the switch can occur when the positive feedback loop cannot be initiated due to an insufficient voltage drop rate. This happens because the charge leakage in C_{in} and C_{drive} is inevitable, and insufficient voltage drop fails to establish an adequate drain-to-source voltage across the P-MOSFET, hindering its conduction. As a result, a high-resistance path forms between C_{drive} and R_{NMOS}, leading to an inadequate gate-source voltage on the N-MOSFET and preventing the switch activation. This issue can be mitigated by enhancing the conduction of either the P-MOSFET or the N-MOSFET through adjustments to C_{in}, C_{drive}, or R_{NMOS}, as detailed below:

1) Increasing C_{in} of the switch mitigates voltage decay caused by its intrinsic charge leakage, allowing the gate-source voltage of the P-MOSFET to establish more quickly during V_{EG} decline. This facilitates P-MOSFET conduction and lowers the minimum voltage drop rate required to trigger the switch (Supplementary Figure 24A). Similarly, increasing C_{drive} will also mitigate voltage decay on it and enhance P-MOSFET conduction, lowering the minimum voltage drop rate needed for triggering the switch (Supplementary Figure 24B).

2) In contrast, increasing R_{NMOS} increases the gate-source voltage of the N-MOSFET for a given conduction resistance between C_{drive} and R_{NMOS}. This promotes the initiation of positive feedback and reduces the minimum voltage drop rate required to trigger the switch (Supplementary Figure 24C).

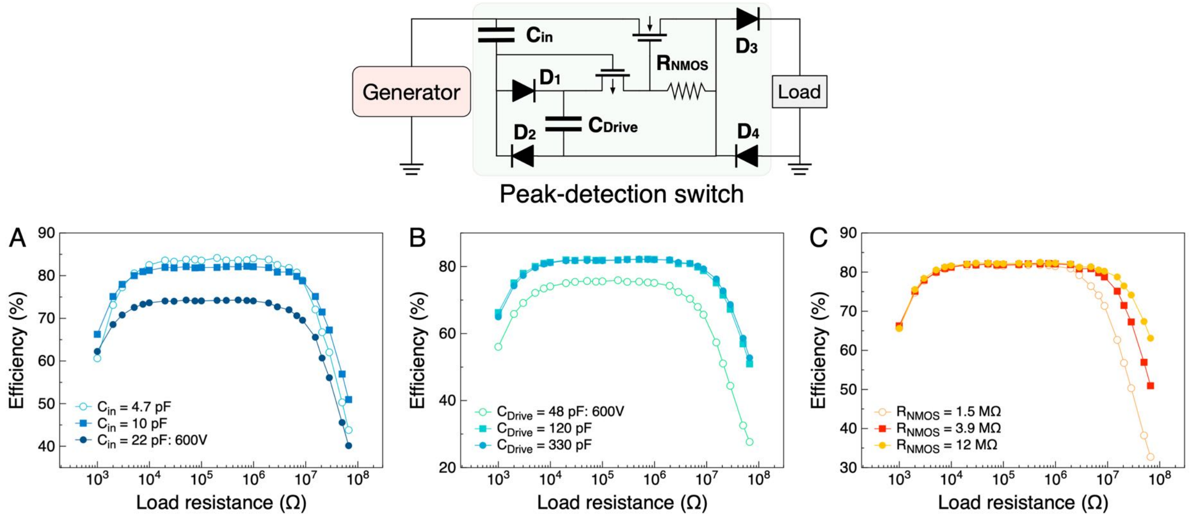

Supplementary Figure 25. Influence of (A) C_{in}, (B) C_{drive}, and (C) R_{NMOS} on switch efficiency with various load resistances. (Voltage drop rate = 20 V ms^{-1}, V_{EG} = 1200V, C_{EG} = 120 pF, unless otherwise noted)

Note:

1) The on-resistance of the N-MOSFET depends on the charge driving it. A larger driving charge can reduce the conduction resistance of the N-MOSFET, lowering the conduction losses, especially at lower load resistances. Thus, increasing C_{in}, which enhances Q_{drive}, improves relative performance at smaller loads compared to its peak performance at moderate load resistances (Supplementary Figure 25A). However, increasing C_{in} also raises driving power consumption, reducing the overall switch efficiency in experiments.

2) The switch closure time is determined by the duration for which C_{drive} drives the N-MOSFET. This can be approximated as three times the R_{NMOS}-C_{drive} discharge time constant (3 τ). Thus, increasing C_{drive} or R_{NMOS} extends this time constant, prolonging N-MOSFET conduction and improving the output performance at higher resistances. However, an excessively large C_{drive} can reduce the driving voltage, preventing a continuous increase in switch closure time.

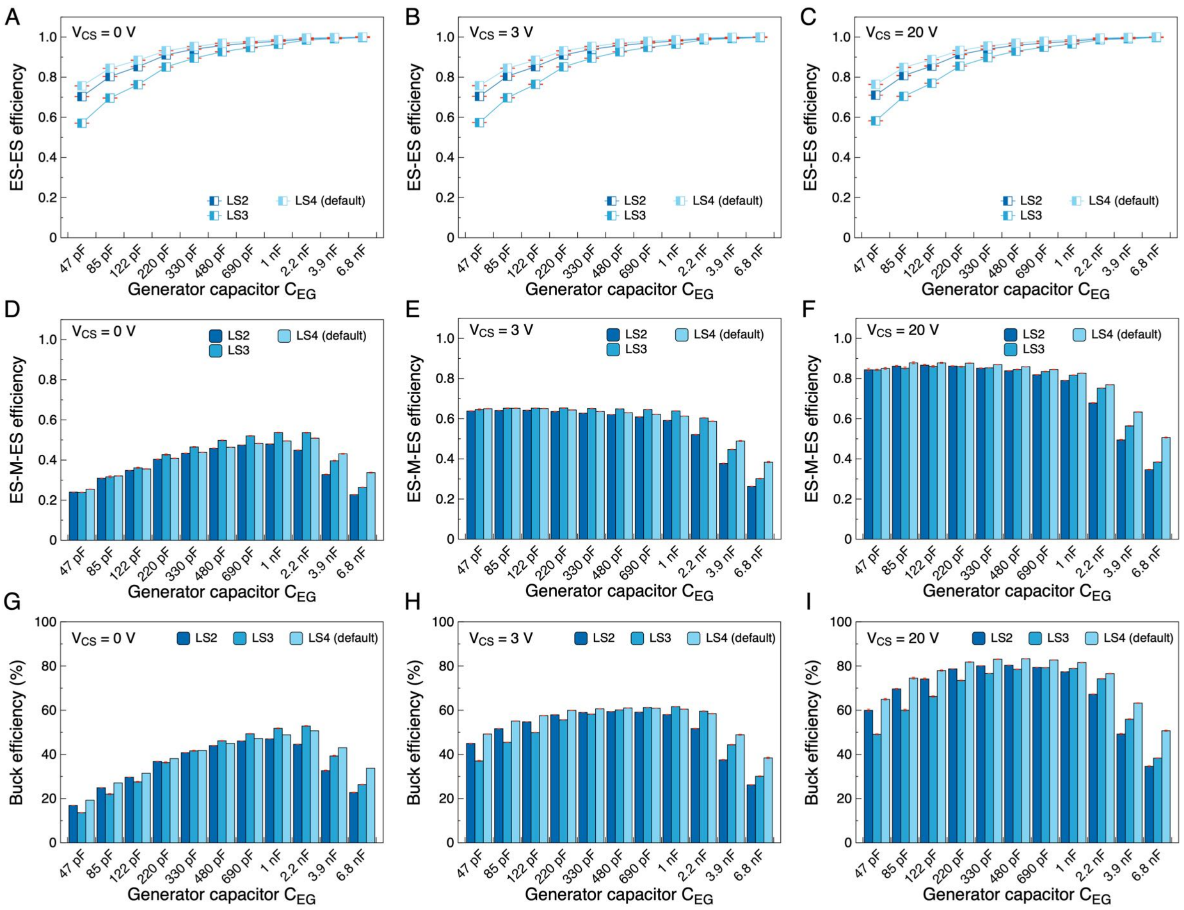

Supplementary Figure 26. Influence of the generator capacitance C_{EG} on the buck efficiency.(A–C) ES-ES efficiency (Phase 1) with different C_{EG} values at V_{CS} = 0, 3, 20V.(D–F) ES-M-ES efficiency (Phase 2&3) with different C_{EG} values at V_{CS} = 0, 3, 20V.(G–I) Buck efficiency with different C_{EG} values at V_{CS} = 0, 3, 20V. Efficiency values are presented as the mean ± s.d. from n = 5 independent experiments.

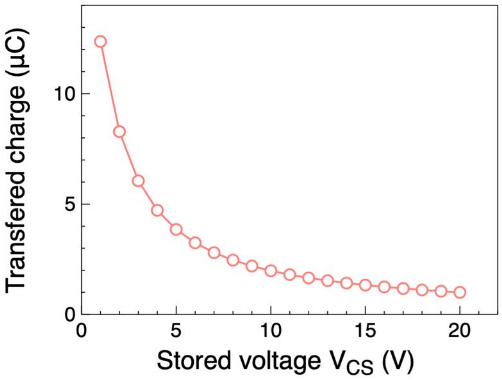

Supplementary Figure 27. Calculated transferred charge versus stored voltage (V_{CS}) for delivering 20 μJ of energy to a 10 μF storage capacitor (C_S).

Note:

The stored energy per transfer cycle (ΔE) is given by:

$$\Delta E = \frac{1}{2}C_S((V_0 + \Delta V)^2 - V_0^2) = \frac{1}{2}\left(\frac{(Q_0 + \Delta Q)^2}{C_S} - \frac{Q_0^2}{C_S}\right)$$

where V_0 and Q_0 represent the initial voltage and charge stored on the capacitor C_S of each transfer cycle, respectively. By solving the equation above, the transferred charge per cycle at V_0 is obtained as:

$$\Delta Q = \sqrt{Q_0^2 + 2C_S\Delta E} - Q_0 = C_S \times \left(\sqrt{V_0^2 + \frac{2\Delta E}{C_S}} - V_0\right)$$

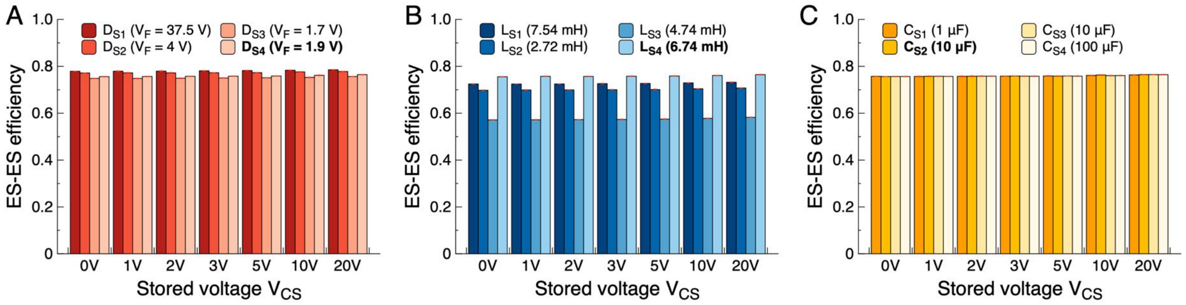

Supplementary Figure 28. ES-ES efficiency under different (A) freewheel diode D_S, (B) inductor L_S, and (C) stored capacitor C_S (C_{EG} = 47 pF, V_{EG} = 1000 V). Efficiency values are presented as the mean ± s.d. from n = 5 independent experiments.

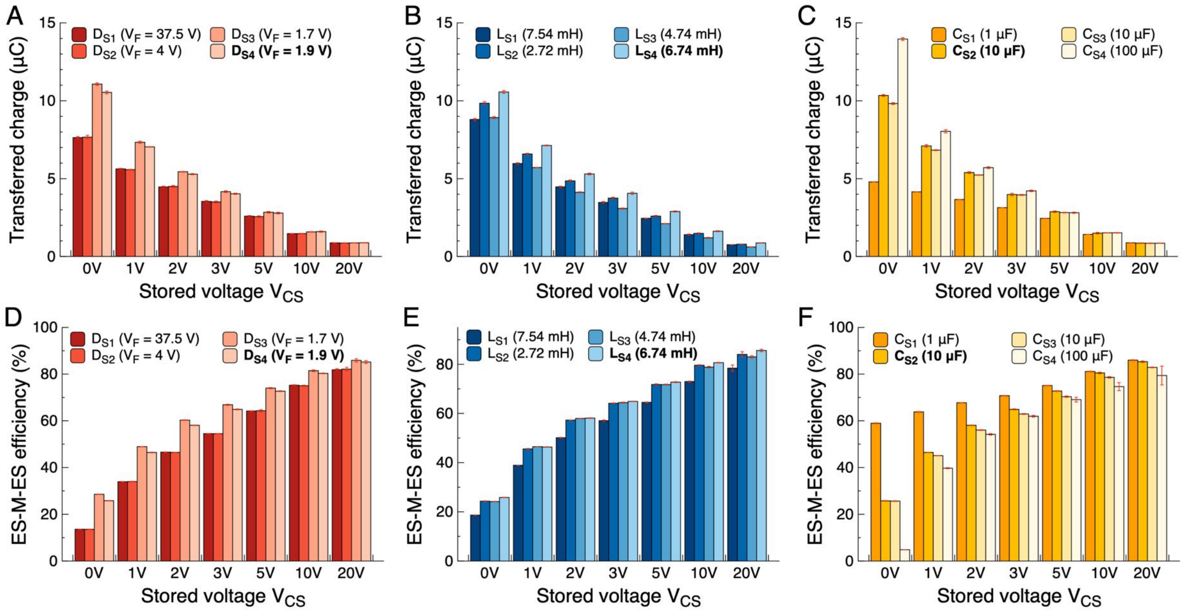

Supplementary Figure 29. (A–C) Transferred charge during a complete energy transfer with varying stored voltages V_{CS} under different buck configurations. (D–F) ES-M-ES efficiency with varying stored voltages V_{CS} (C_{EG} = 47 pF, V_{EG} = 1000 V). Efficiency values are presented as the mean ± s.d. from n = 5 independent experiments.

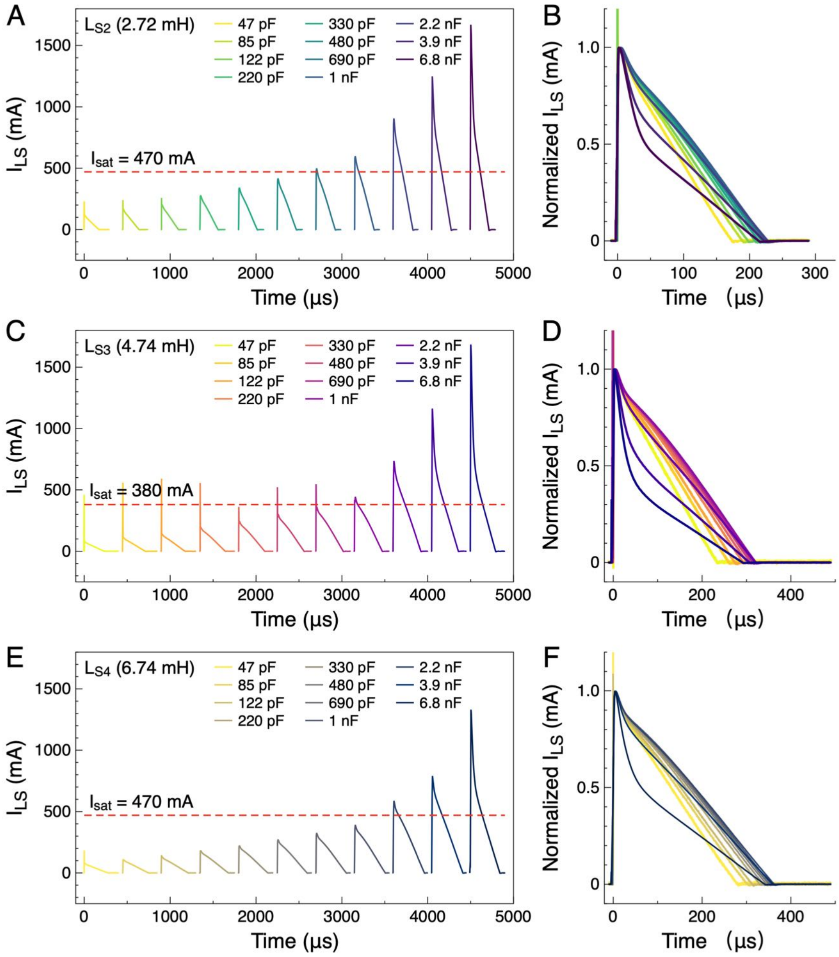

Supplementary Figure 30. Waveforms of I_{LS} through (A) L_{S2}, (C) L_{S3}, and (E) L_{S4} at different generator capacitances, along with their corresponding normalized I_{LS} in (B), (D), and (F) to facilitate the observation of waveform distortion after saturation.

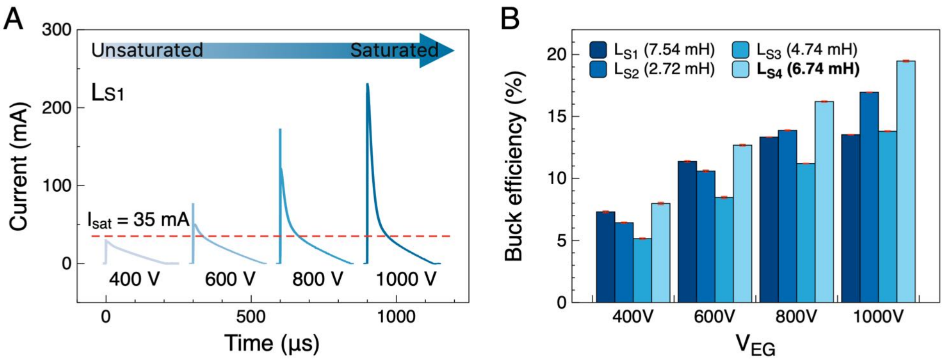

Supplementary Figure 31. (A) Waveforms of I_{LS} through L_{S1} and (B) buck efficiency for different L_S configurations under varying generator voltages (C_{EG} = 47 pF), presented as the mean ± s.d. from n = 5 independent experiments.The rated saturation current of L_{S1} is approximately 35 mA, as specified in its datasheet. At higher V_{EG} values (> 600 V), the energy transfer demand exceeds the L_{S1} limit and makes it enter saturation, as evidenced by pulse-like distortions in the I_{LS} waveform. As a result, the efficiency of the L_{S1} configuration decreases.

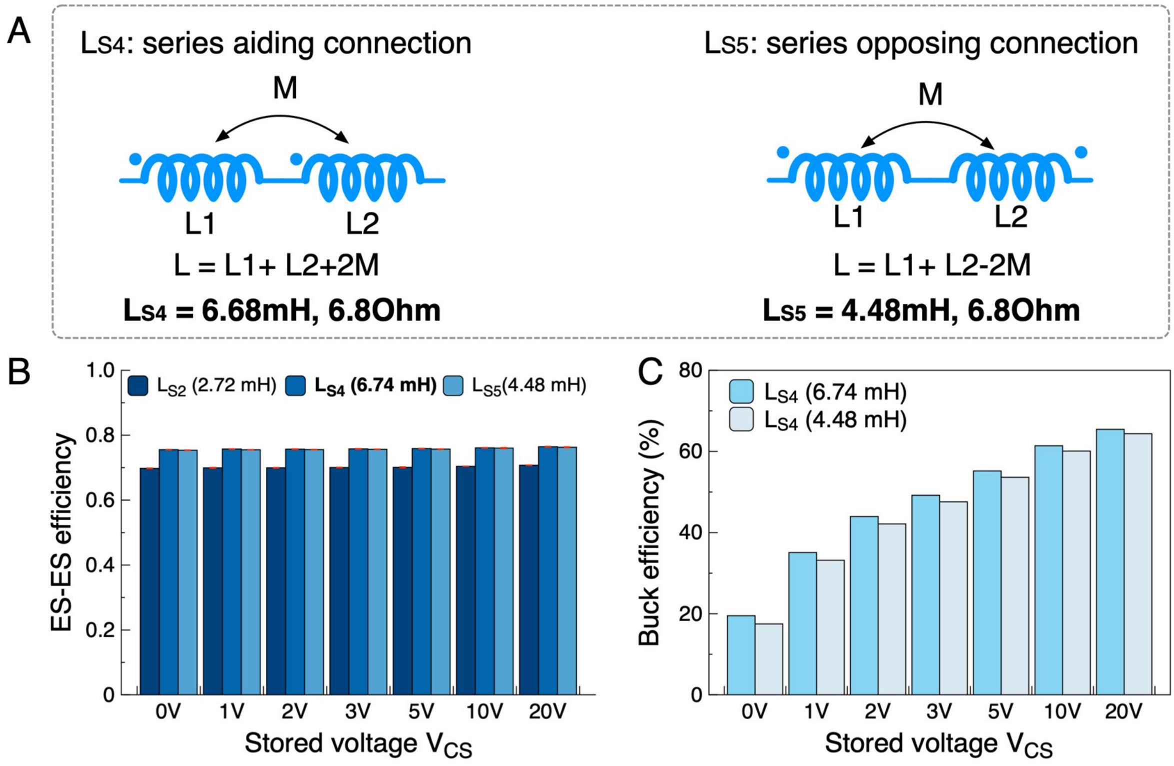

Supplementary Figure 32. (A) Schematic of series-aiding and series-opposing inductor configurations. (B) Comparison of ES-ES loss among single-inductor and series-connected inductor configurations. Efficiency values are presented as the mean ± s.d. from n = 5 independent experiments. (C) Buck conversion efficiency using series-aiding (L_{S4}) and series-opposing (L_{S5}) inductor connections.

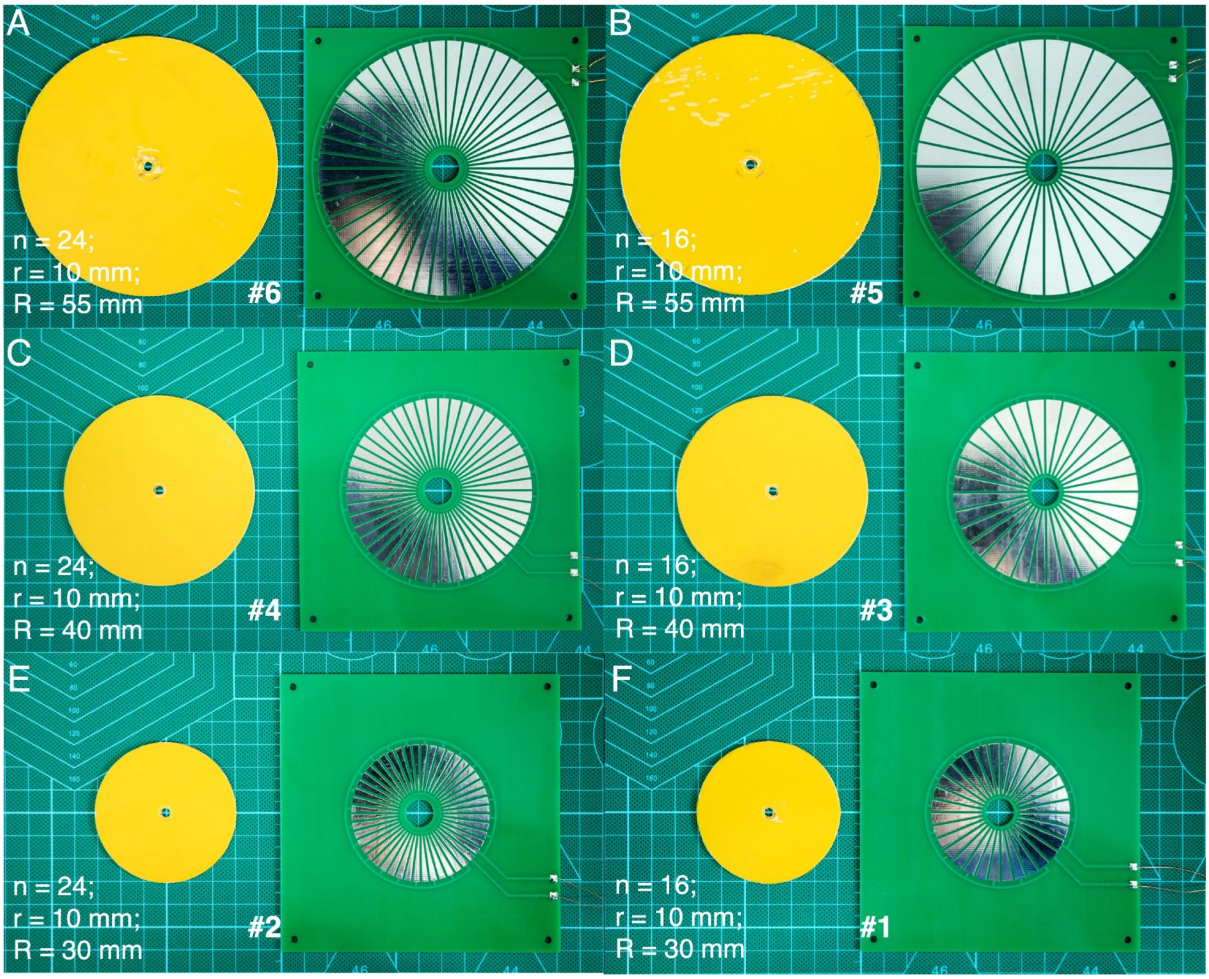

Supplementary Figure 33. Photographs of generators with different physical configurations.

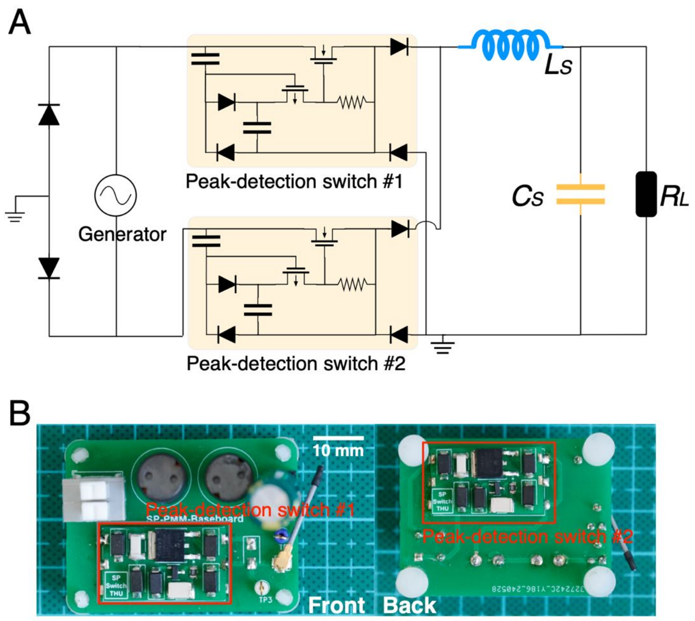

Supplementary Figure 34. (A) Schematic and (B) photographs of the switches and LC buck converter used in the tests with rotational electret generators with bipolar output.

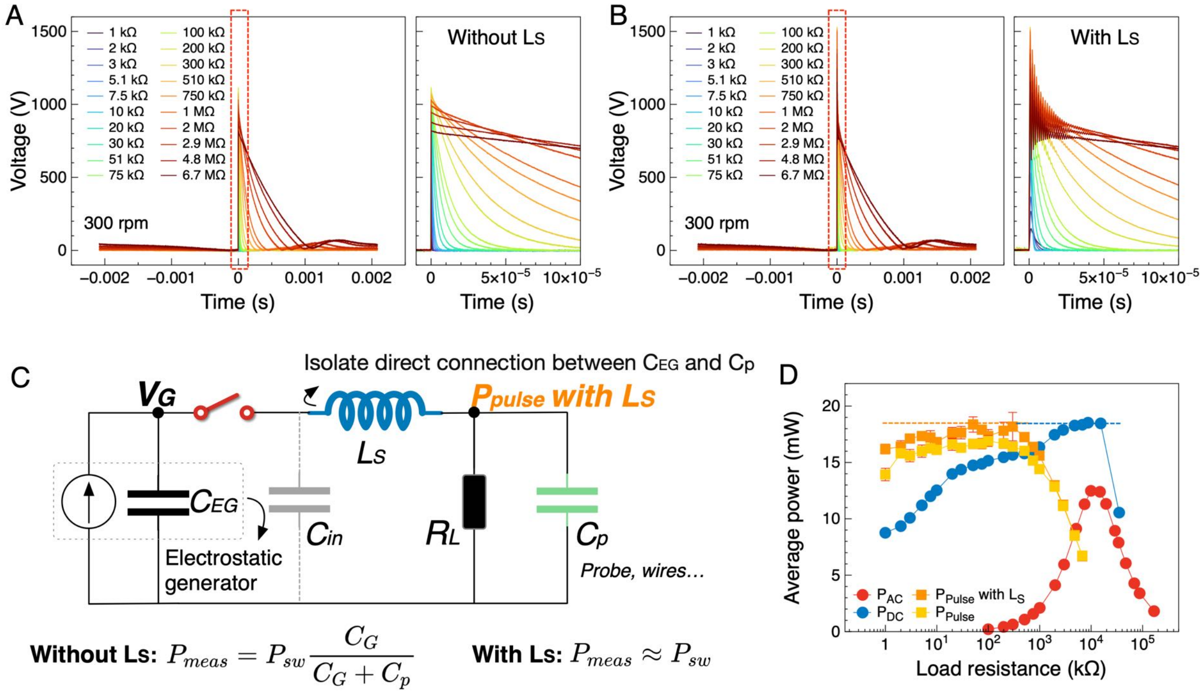

Supplementary Figure 35. Waveforms of pulsed output (A) without L_S and (B) with L_S. (C) Schematic of the testing circuit for pulsed output with L_S. (D) Comparison of average output power among direct AC output, pulsed output, and DC output, including the P_{pulse} output with L_S. L_{S4} is used in this experiment. P_{pulse} values are presented as the mean ± s.d. from n = 5 independent experiments.

Note:

The underestimation of P_{pulse} primarily originates from parasitic capacitance (C_p) on the load side, caused by wiring and test probe parasitic. This C_p reduces the output voltage across the load resistor (R_L), lowering the measured power. Inserting an inductor between the switch and the load (Supplementary Figure 35C) effectively isolates the impact of C_p, ensuring that nearly all post-switch energy transfers to the load, similar to the ES-M-ES energy transfer process. This improves alignment between the measured power (P_{meas}) and the actual post-switch output (P_{truth}).

However, parasitic capacitance before the inductor still introduces energy loss. This pre-inductor loss corresponds to the ES-ES loss of the buck converter. As a result, the pulsed output with L_S and the DC output from the buck converter exhibits nearly identical maximum average power. This equivalence arises because ES-M-ES losses become negligible in buck converters at high V_{CS} (several hundred volts, Supplementary Figure 4C), while the ES-ES loss remains the same in both configurations.

The AC power was measured using a custom-built circuit with an input resistance of 1 GΩ and input capacitance below 0.3 pF, ensuring no underestimation occurred during the measurement.

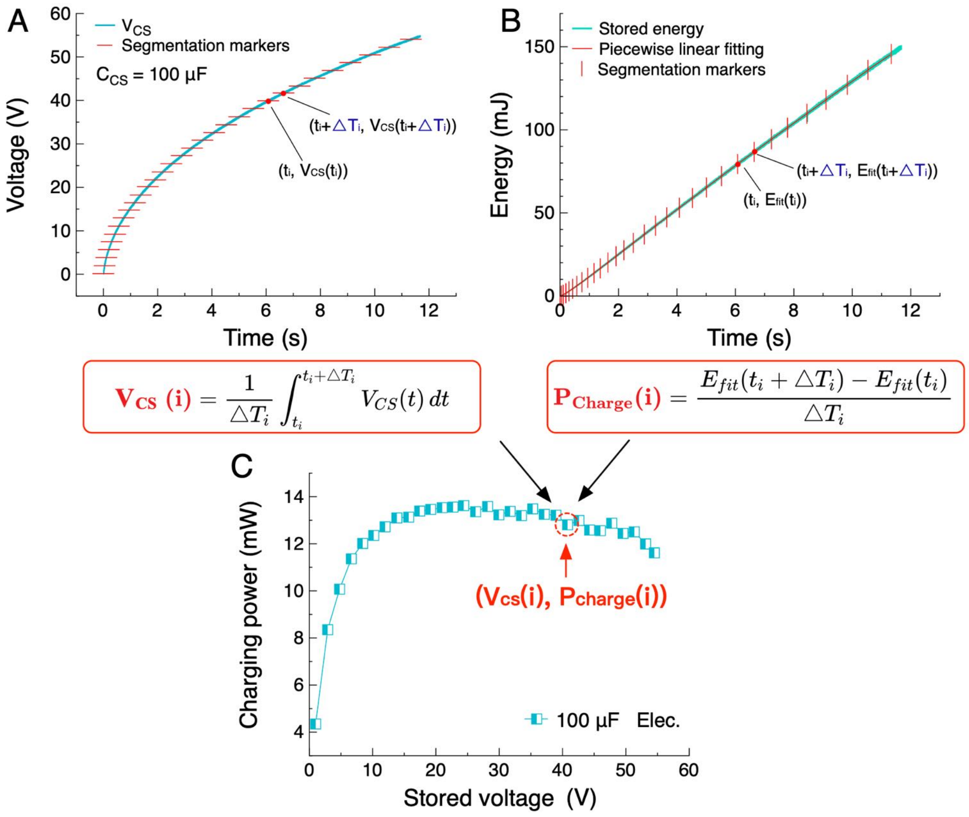

$$\mathbf{V_{CS}\ (i)} = \frac{1}{\triangle T_i}\int_{t_i}^{t_i+\triangle T_i} V_{CS}(t)\,dt$$

$$\mathbf{P_{Charge}\,(i)} = \frac{E_{fit}(t_i+\triangle T_i) - E_{fit}(t_i)}{\triangle T_i}$$

Supplementary Figure 36. Method for deriving the charging power (P_{charge}) and stored voltage (V_{charge}). The charging power curve is constructed by calculating individual data points and aggregating the results. Voltage increments of 2 V were used in the calculation.

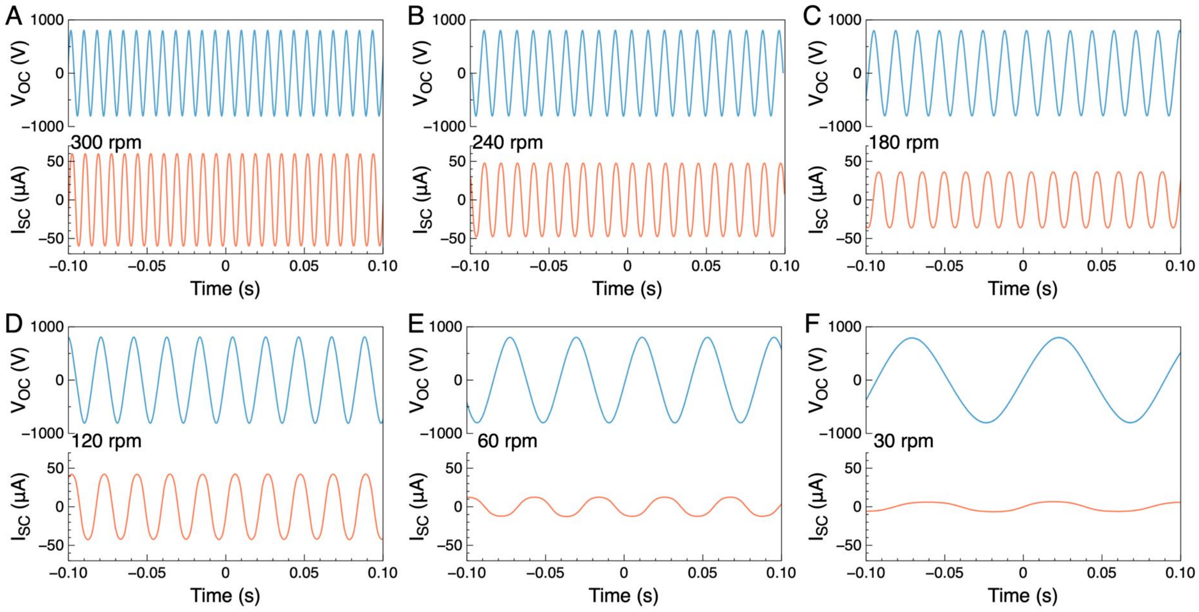

Supplementary Figure 37. Open-circuit voltage and short-circuit current of the generator at rotation speeds ranging from 30 to 300 rpm (n = 24; r = 10 mm; R = 55 mm, interval = 1mm).

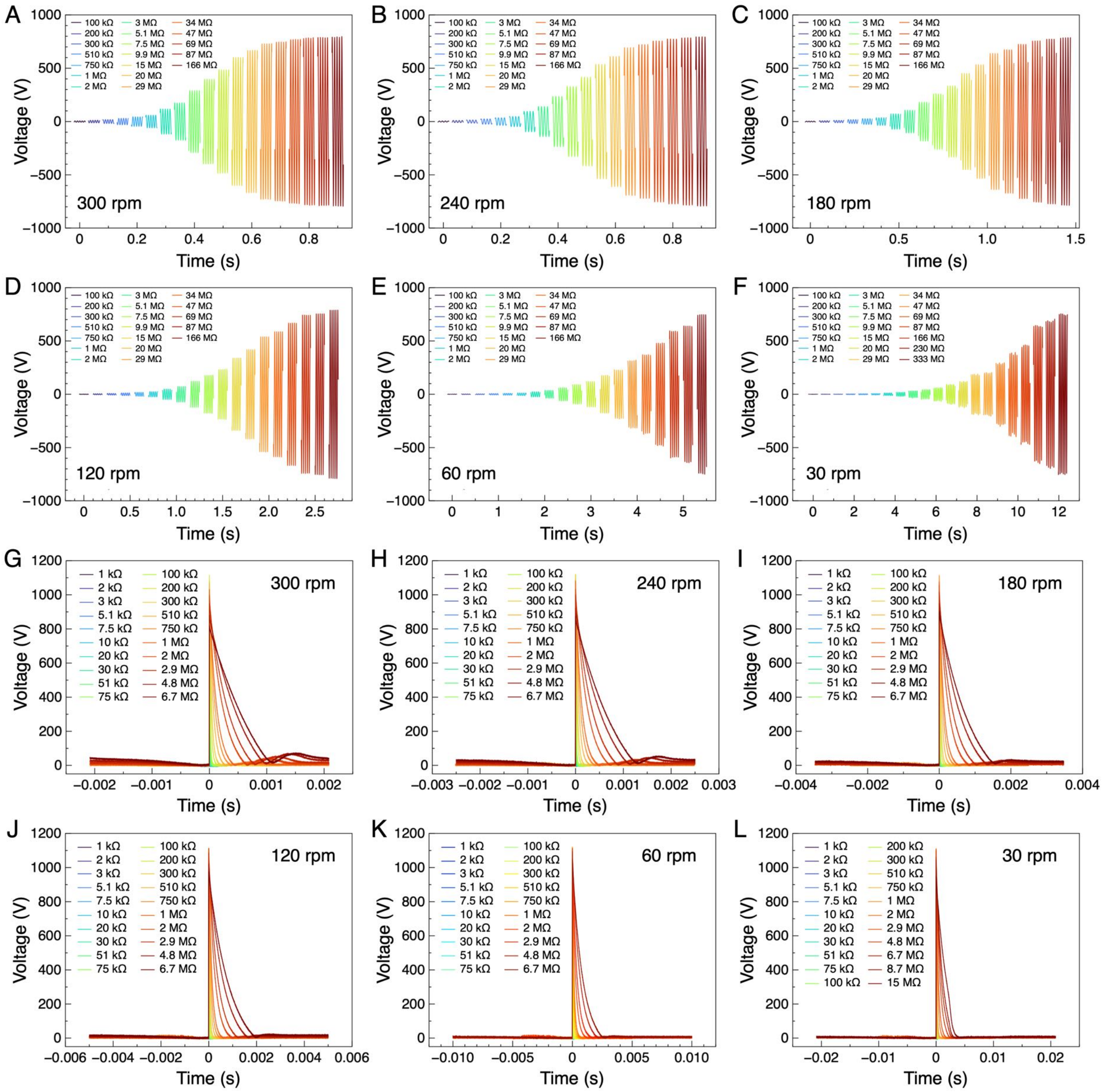

Supplementary Figure 38. (A–F) Direct AC output voltage from the generator and (G–L) pulsed output voltage after the peak-detection switch at rotation speeds ranging from 30 to 300 rpm (n = 24; r = 10 mm; R = 55 mm, interval = 1 mm).

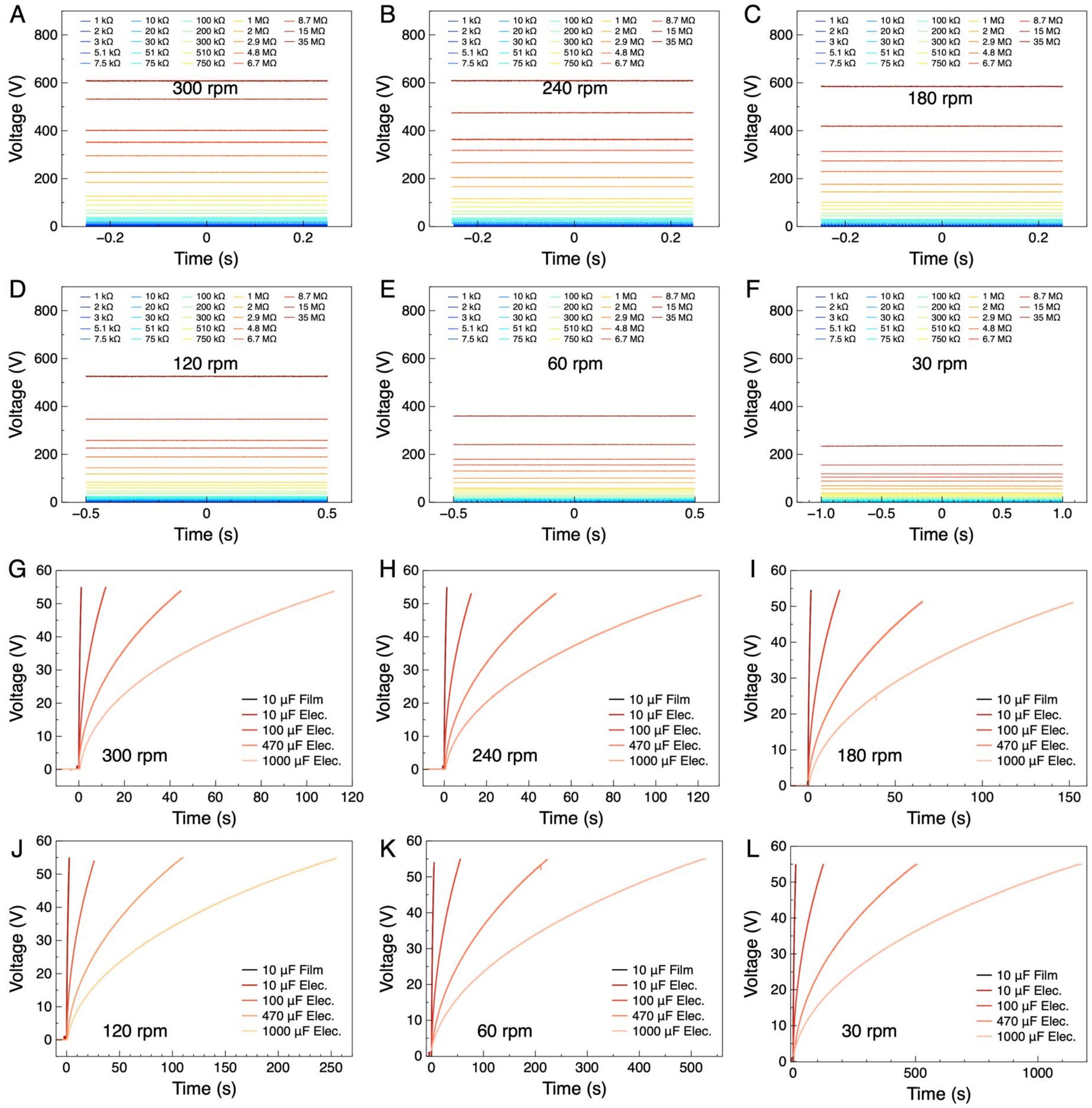

Supplementary Figure 39. (A–F) DC output voltage after the buck converter, and (G–L) charging curves of different capacitors at rotation speeds ranging from 30 to 300 rpm (n = 24; r = 10 mm; R = 55 mm, interval = 1 mm).

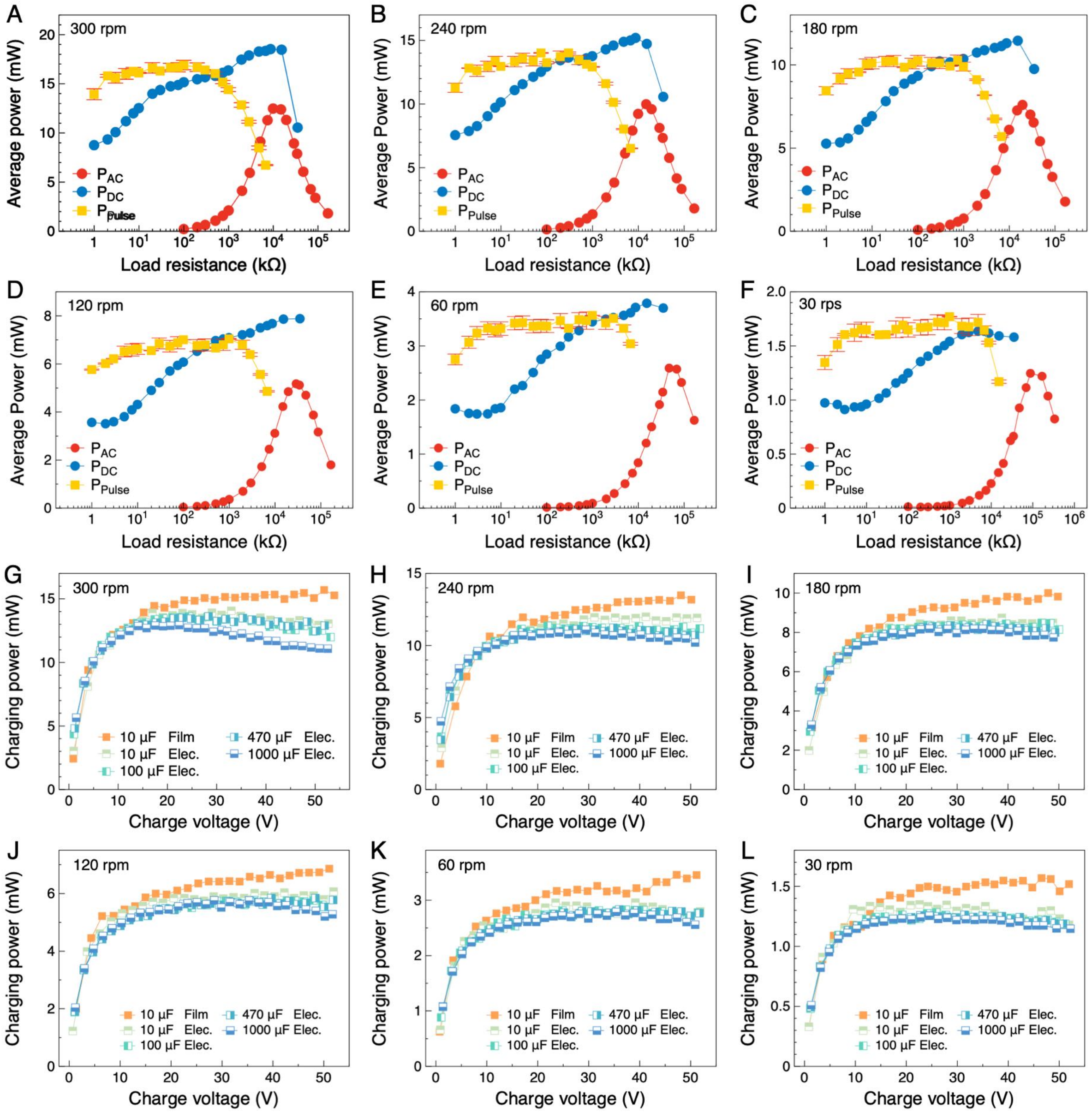

Supplementary Figure 40. (A–F) Comparison of the average output power of the direct AC output, the pulsed output, and the DC output, and (G–L) charging power of various capacitors versus the stored voltages at rotation speeds ranging from 30 to 300 rpm (n = 24; r = 10 mm; R = 55 mm, interval = 1 mm). P_{pulse} values are presented as the mean ± s.d. from n = 5 independent experiments.

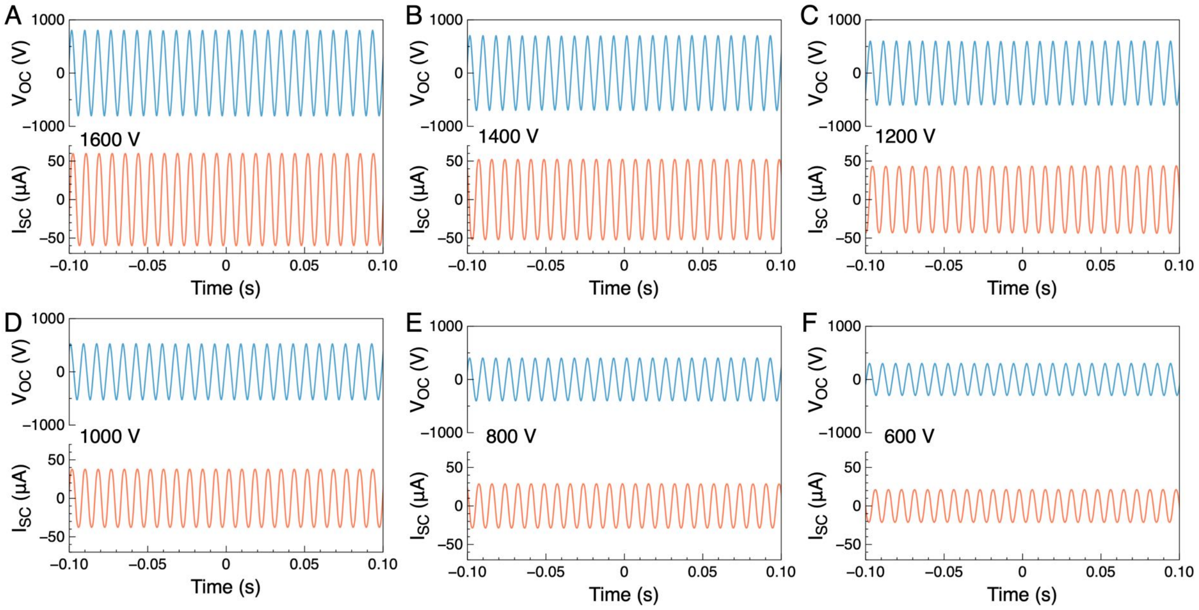

Supplementary Figure 41. Open-circuit voltage and short-circuit current of the generator, with open-circuit voltage ranging from 600 V_{pp} to 1600 V_{pp} (n = 24, r = 10 mm, R = 55 mm, interval = 1 mm, 300 rpm). The open-circuit voltage is adjusted by varying the gap between the rotor and stator.

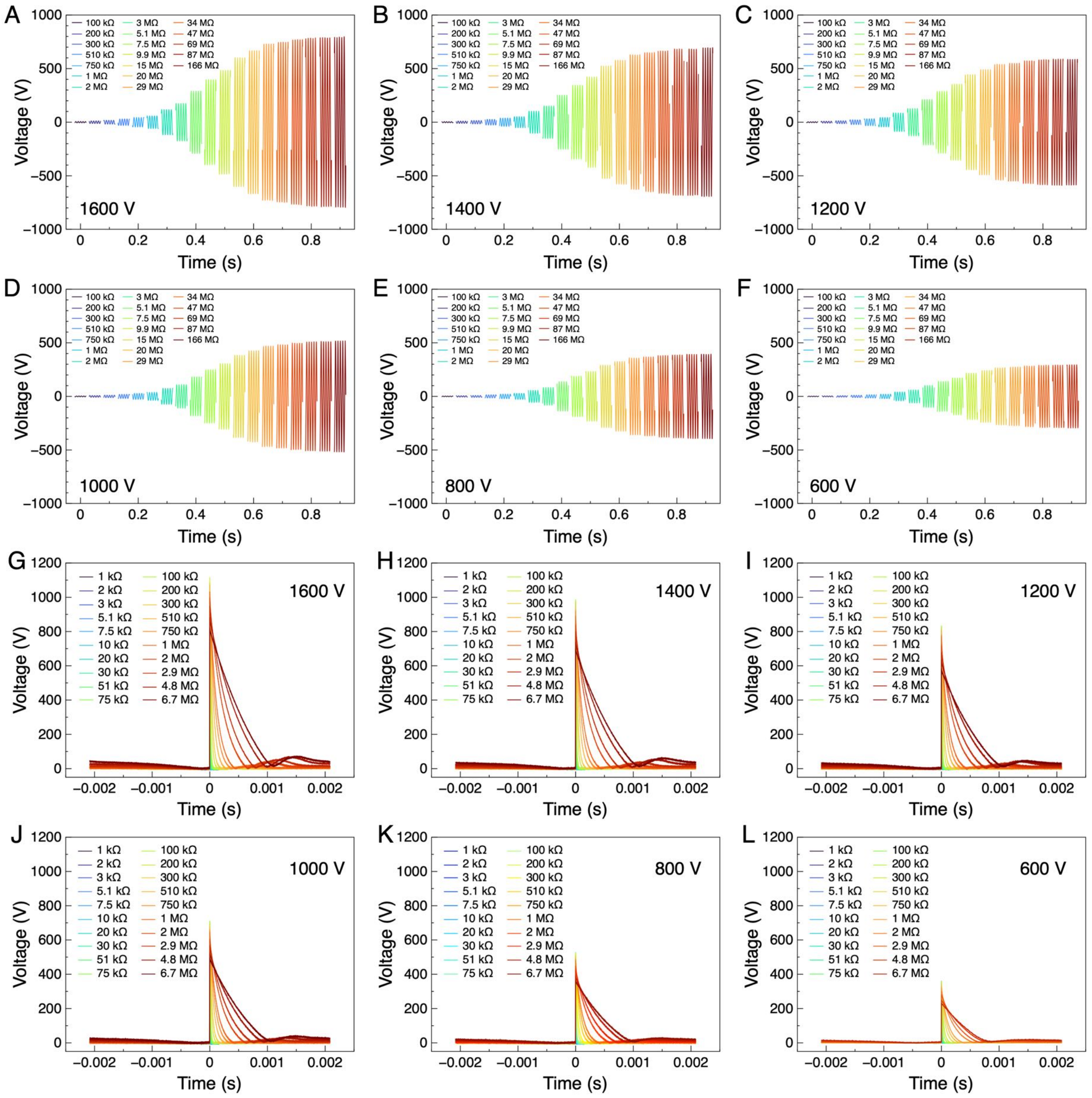

Supplementary Figure 42. (A–F) Direct AC output voltage from the generator. (G–L) pulsed output voltage after the peak-detection switch, with the open-circuit voltage ranging from 600 V_{pp} to 1600 V_{pp} (n = 24; r = 10 mm; R = 55 mm, interval = 1 mm, 300 rpm).

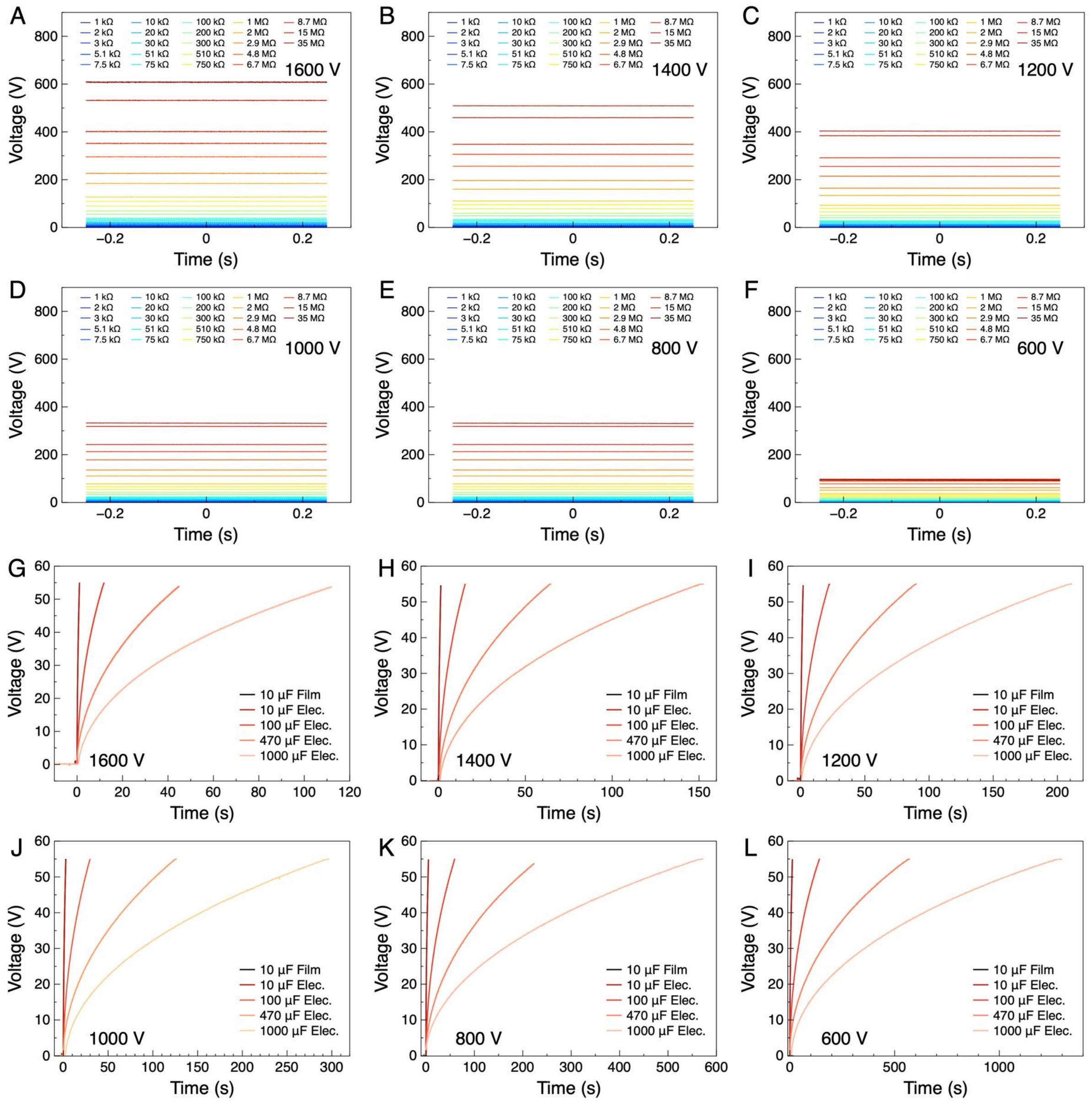

Supplementary Figure 43. (A–F) DC output voltage after the buck converter. (G–L) charging curves of different capacitors, with the open-circuit voltage ranging from 600 V_{pp} to 1600 V_{pp} (n = 24; r = 10 mm; R = 55 mm, interval = 1 mm, 300 rpm).

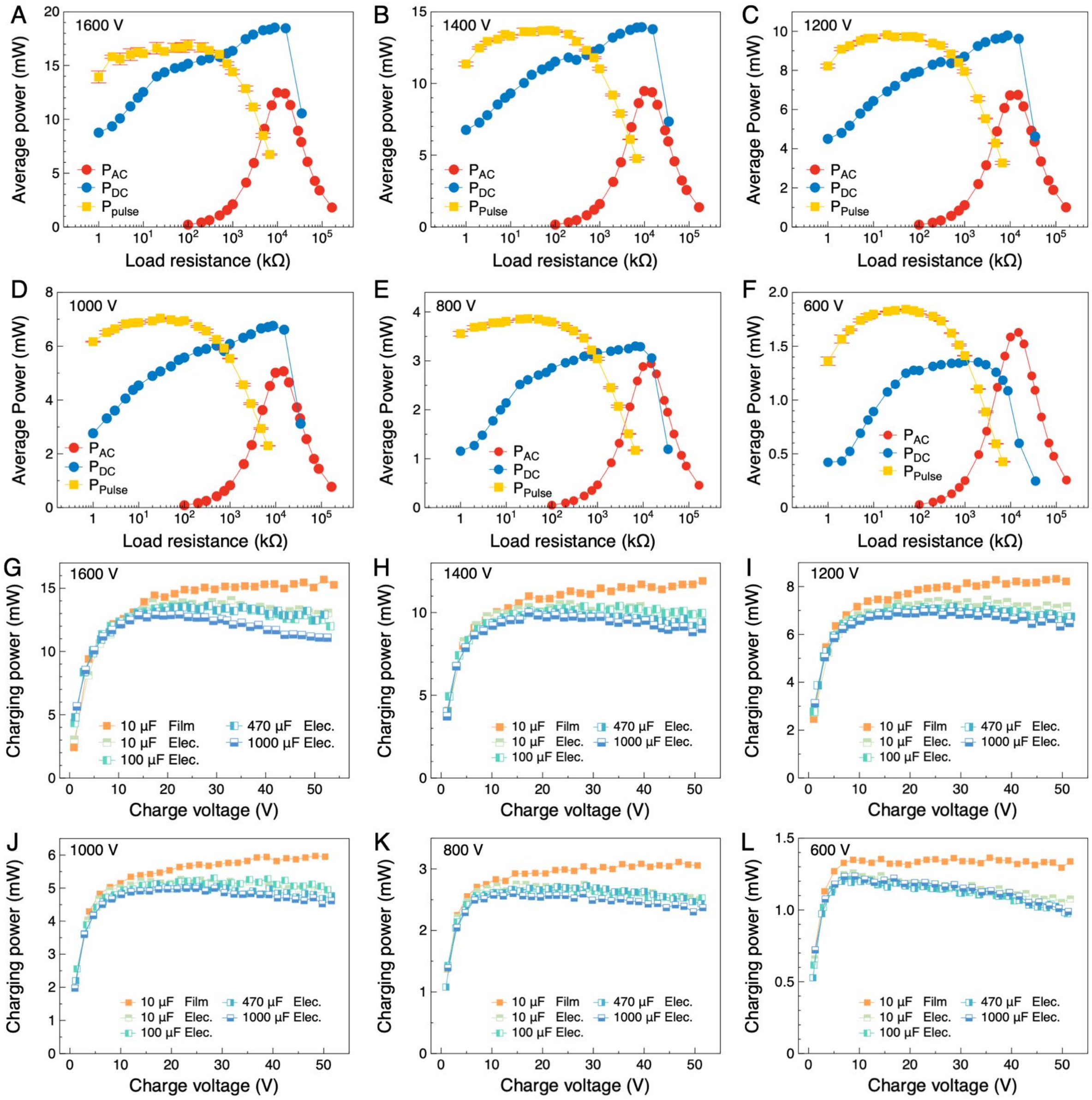

Supplementary Figure 44. (A–F) Comparison of the average output power of the direct AC output, the pulsed output, and the DC output, and (G–L) charging power of various capacitors versus the stored voltages, with the open-circuit voltage ranging from 600 V_{pp} to 1600 V_{pp} (n = 24; r = 10 mm; R = 55 mm, interval = 1 mm, 300 rpm). P_{pulse} values are presented as the mean ± s.d. from n = 5 independent experiments.

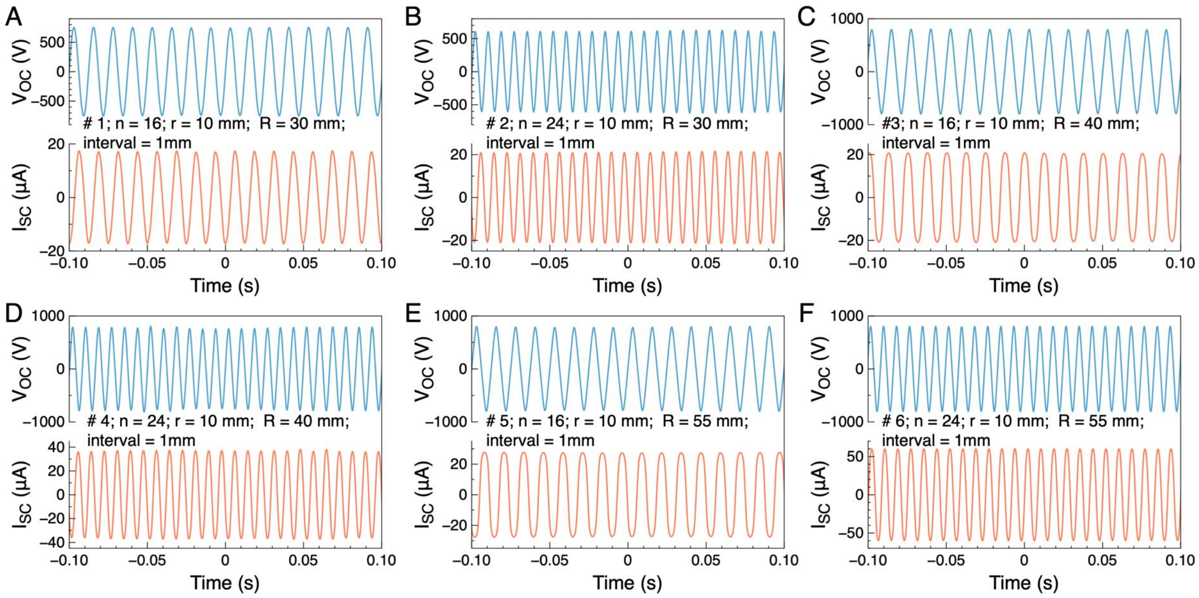

Supplementary Figure 45. Open-circuit voltages and short-circuit currents of the generator with different physical configurations (300 rpm).

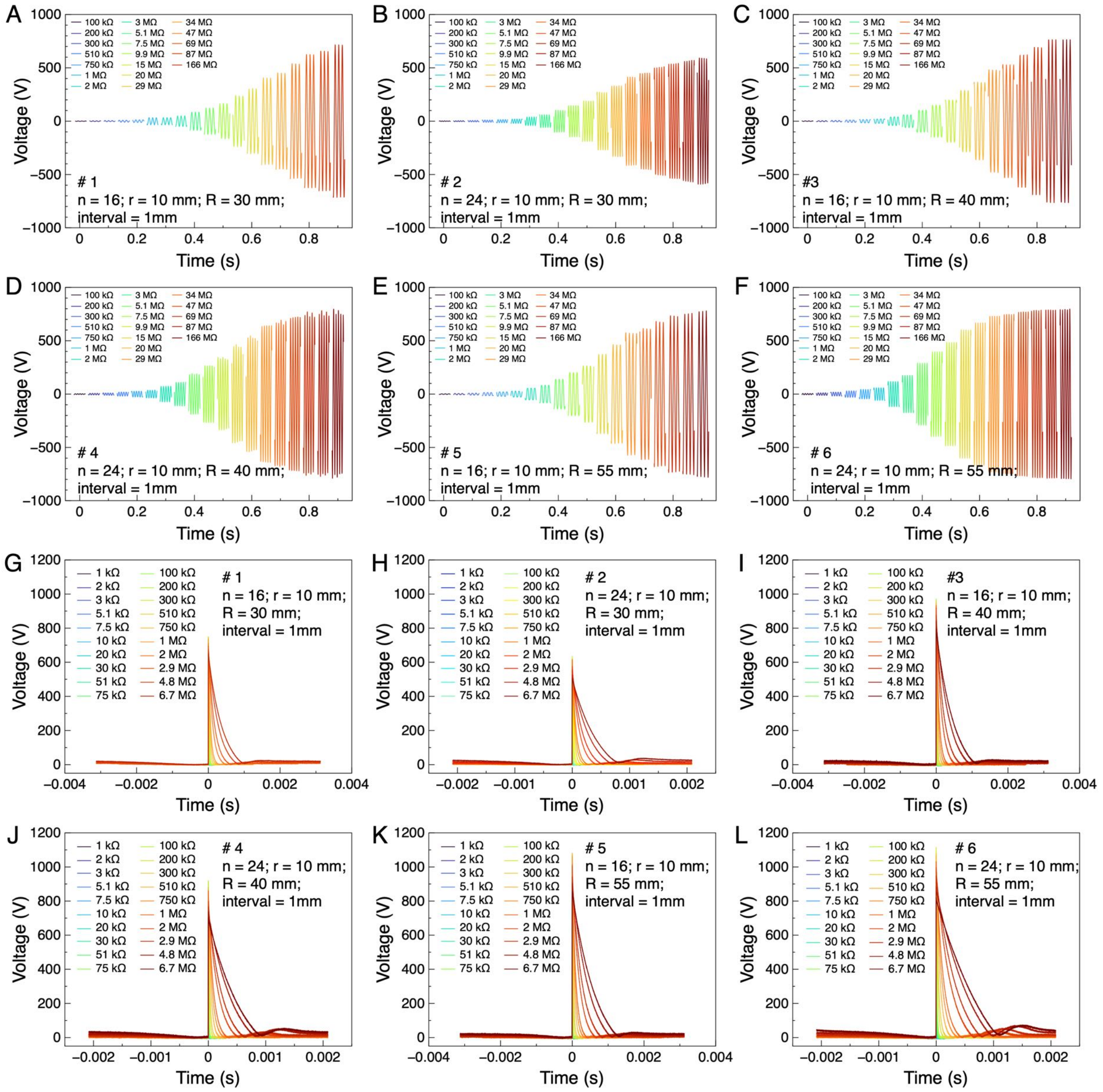

Supplementary Figure 46. (A–F) Direct AC output voltage from the generator. (G–L) pulsed output voltage after the peak-detection switch, with different physical configurations (300 rpm).

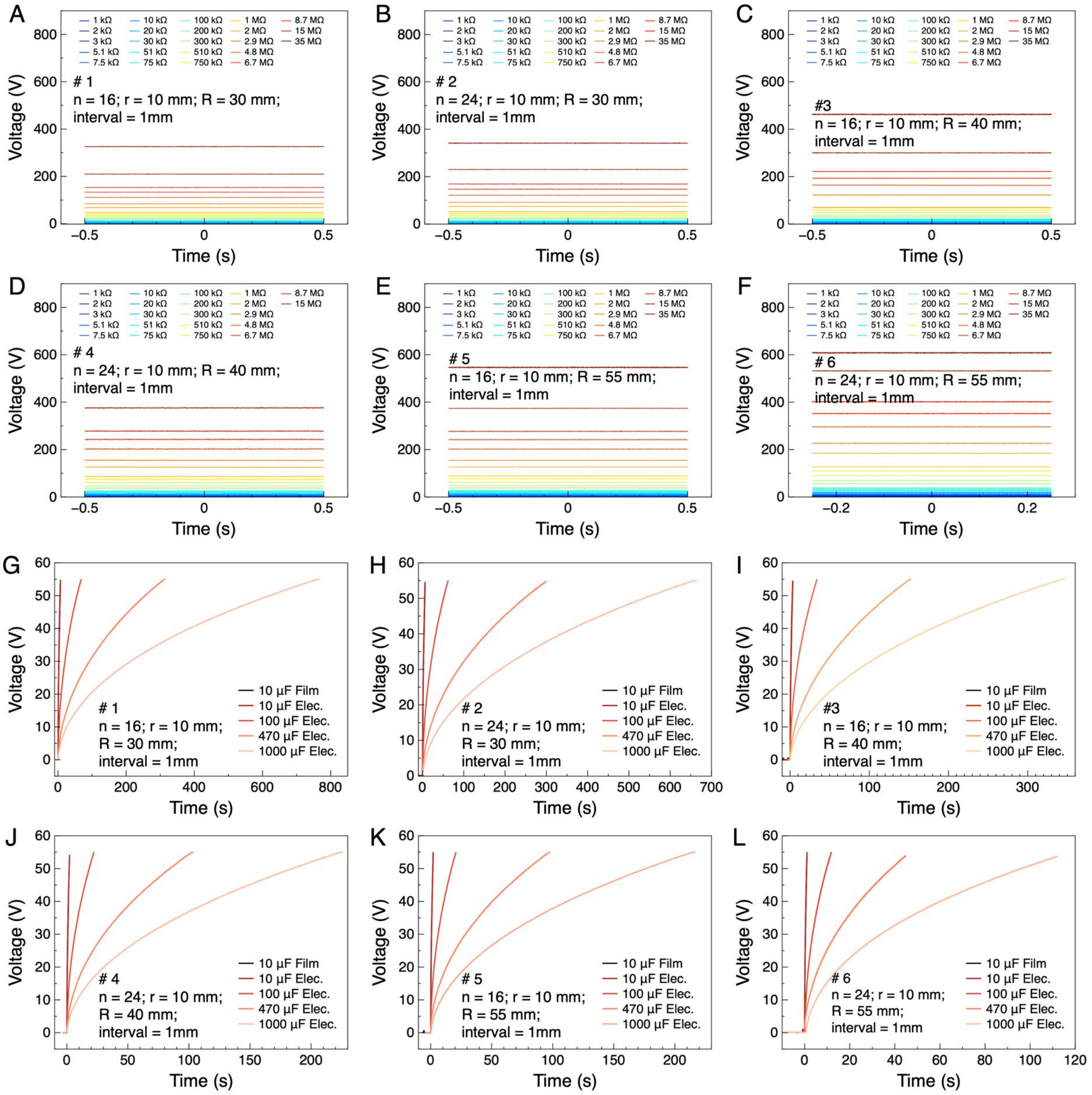

Supplementary Figure 47. (A–F) DC output voltage after the buck converter and (G–L) charging curves of different capacitors with different physical configurations (300 rpm).

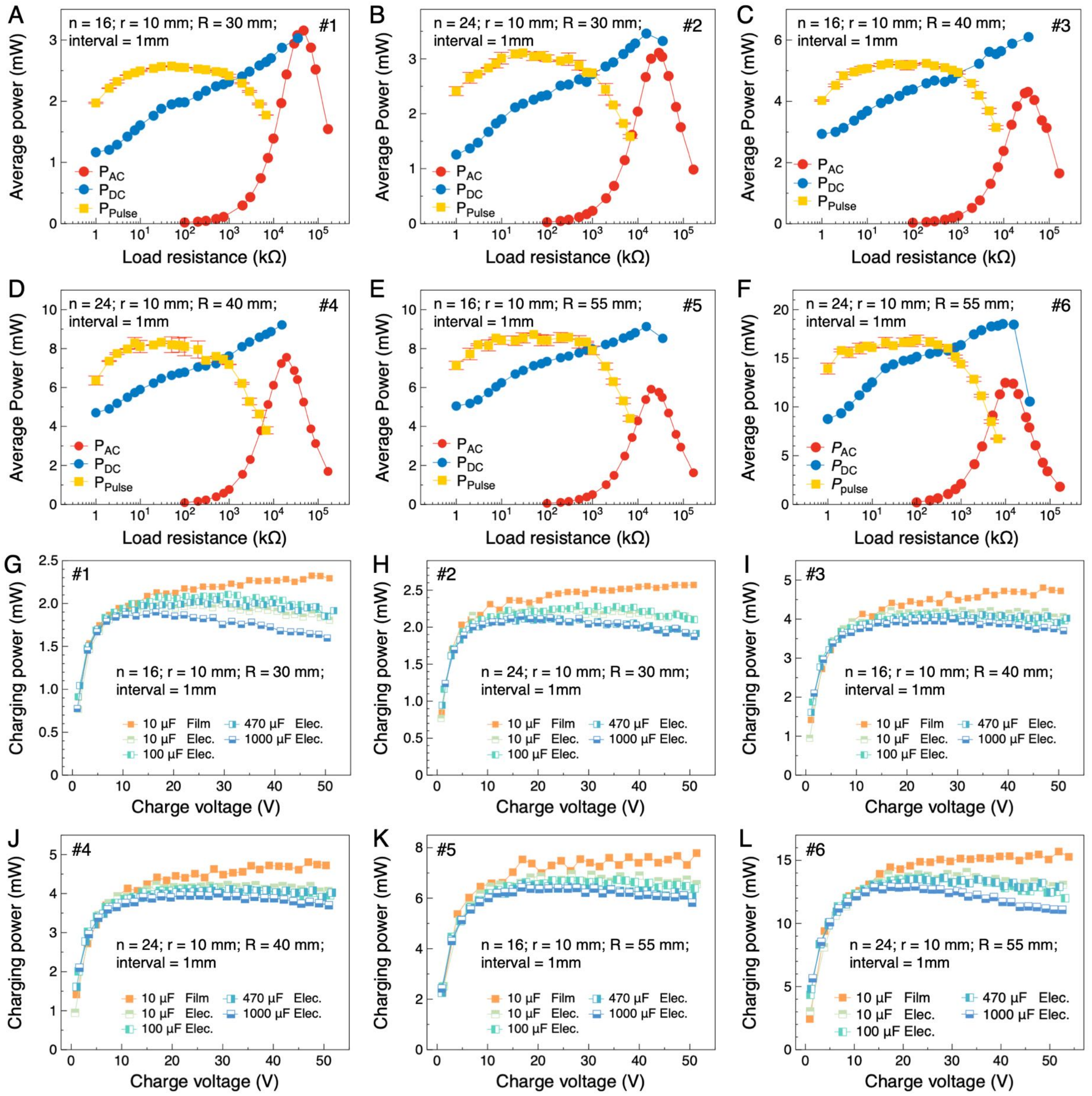

Supplementary Figure 48. (A–F) Comparison of the average output power of the direct AC output, the pulsed output, and the DC output, and (G–L) charging power of various capacitors versus the stored voltages, with different physical configurations (300 rpm). P_{pulse} values are presented as the mean ± s.d. from n = 5 independent experiments.

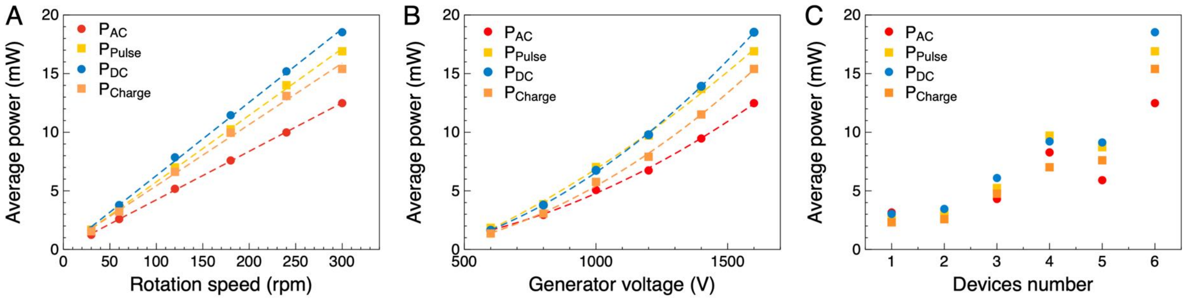

Supplementary Figure 49. Comparison of the average output power of the direct AC output, the pulsed output, the DC output, and charging power with different operation (A) rotation speed, (B) generator voltage, and (C) physical configurations.

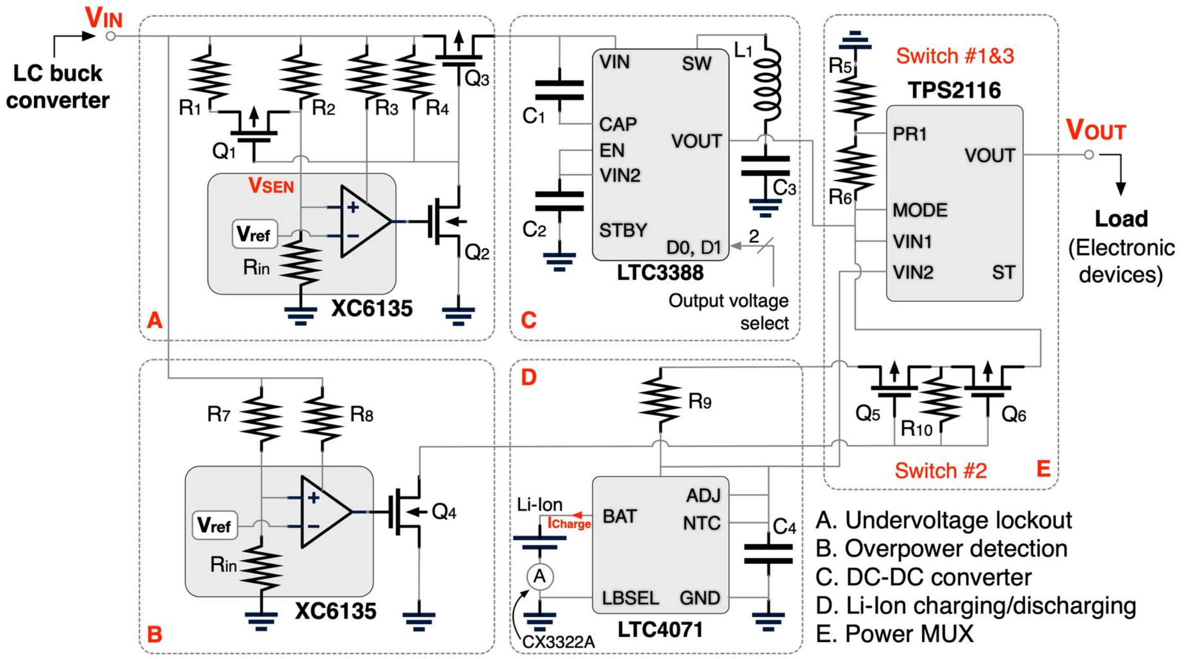

Supplementary Figure 50. Schematic of the energy regulation module.**Note:** Description of functional blocks for energy regulation module

A) Undervoltage lockout (UVLO) module: The UVLO module is implemented using a voltage detector IC (XC6135), which outputs a high level when its sensed voltage (V_{SEN}) is higher than the internal reference voltage (V_{ref}). Initially, the input voltage of the system (V_{IN}) is zero and all MOSFETs (Q_1, Q_2, and Q_3) are in the cut-off state. As the input voltage increases, the voltage applied to the XC6135 (V_{SEN}) rises, and when $V_{SEN} > V_{ref}$, the XC6135 outputs a high level, turning Q_2 and Q_3 on—i.e., the UVLO is activated. The corresponding conduction threshold (upper threshold) can be expressed as:

$$V_{\mathrm{TH,high}} = V_{\mathrm{ref}} \frac{R_{\mathrm{in}} + R_2}{R_{\mathrm{in}}}$$

where R_{in} is the input resistance of the XC6135. We note that when Q_2 conducts, Q_1 is also turned on, which makes V_{SEN} governed simultaneously by R_1 and R_2. As a result, the UVLO remains active until the input voltage drops below

$$V_{\mathrm{TH,low}} = V_{\mathrm{ref}} \frac{R_{\mathrm{in}} + (R_2 \parallel R_1)}{R_{\mathrm{in}}}$$

Here, R_3 is used to limit the supply current to protect the XC6135 at high V_{IN}, and R_4 helps ensure reliable cut-off when the XC6135 output is low.

B) Overpower detection module: the overpower detection is realized by voltage detection circuit based on XC6135. The principle is straightforward: when the input power exceeds the load consumption, the input voltage V_{IN} (i.e., the voltage across the LC buck capacitor) increases continuously; conversely, when the input power is lower than the load consumption, V_{IN} decreases continuously. Thus, we set its threshold slightly higher (~0.1 V) than the upper threshold of the UVLO module. Once V_{IN} exceeds this threshold, it indicates that the input power is greater than the load consumption, thereby enabling switch #2 in the Power MUX

module to divert the excess energy into the battery.

C) DC-DC converter: The design of the DC-DC converter is based on the LTC3388 family and follows the standard application circuit provided in its datasheet. Two variants of the device are available: LTC3388-1, which allows output voltages of 1.2V, 1.5V, 1.8V, or 2.5V, and LTC3388-3, which allows 2.8V, 3.0V, 3.3V, or 5V. The output voltage is selected via the settings of pins D_0 and D_1.

D) Li-Ion charging/discharging module: A Li-ion charging/discharging circuit based on the LTC4071 is designed following the standard application circuit in the datasheet, providing protection against battery overcharge and overdischarge. The charging current is set by the resistor R_9. A Device Current Waveform Analyzer (Keysight, CX3322A) with CX1101A probe is employed to monitor the charging and discharging current of the battery.

E) Power multiplexer (MUX) module: The power MUX consists of three switches. Switches #1 and #3 are realized using TP2116, configured in automatic priority mode, giving priority to V_{IN1} and switching to V_{IN2} when V_{IN1} drops. Thus, when the DC-DC converter (V_{IN1}) provides a valid output, it is connected to the load, while the battery (V_{IN2}) automatically takes over if the converter cannot deliver power due to excessive load demand or insufficient harvested energy. Switch #2 consists of two back-to-back P-MOSFETs (Q_5, Q_6) and is solely controlled by the overpower detection module, enabling excess harvested energy to be diverted to the battery.

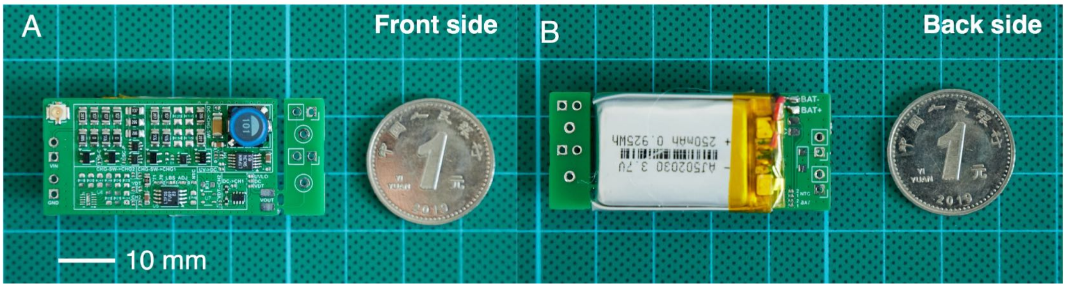

Supplementary Figure 51. Photograph of the energy regulation module.

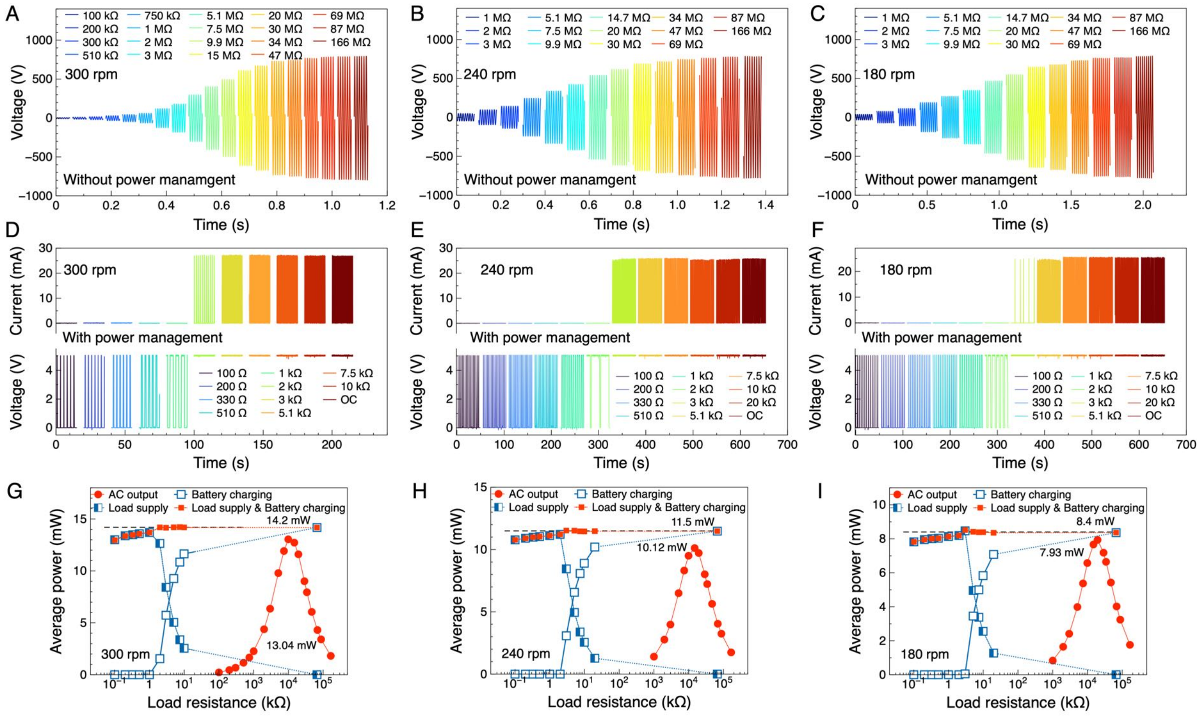

Supplementary Figure 52. Waveforms of (A–C) direct AC outputs without power management, (D–F) DC outputs with power management, and (G–I) average power comparisons with and without power management at rotation speeds of 300, 240, and 180 rpm.

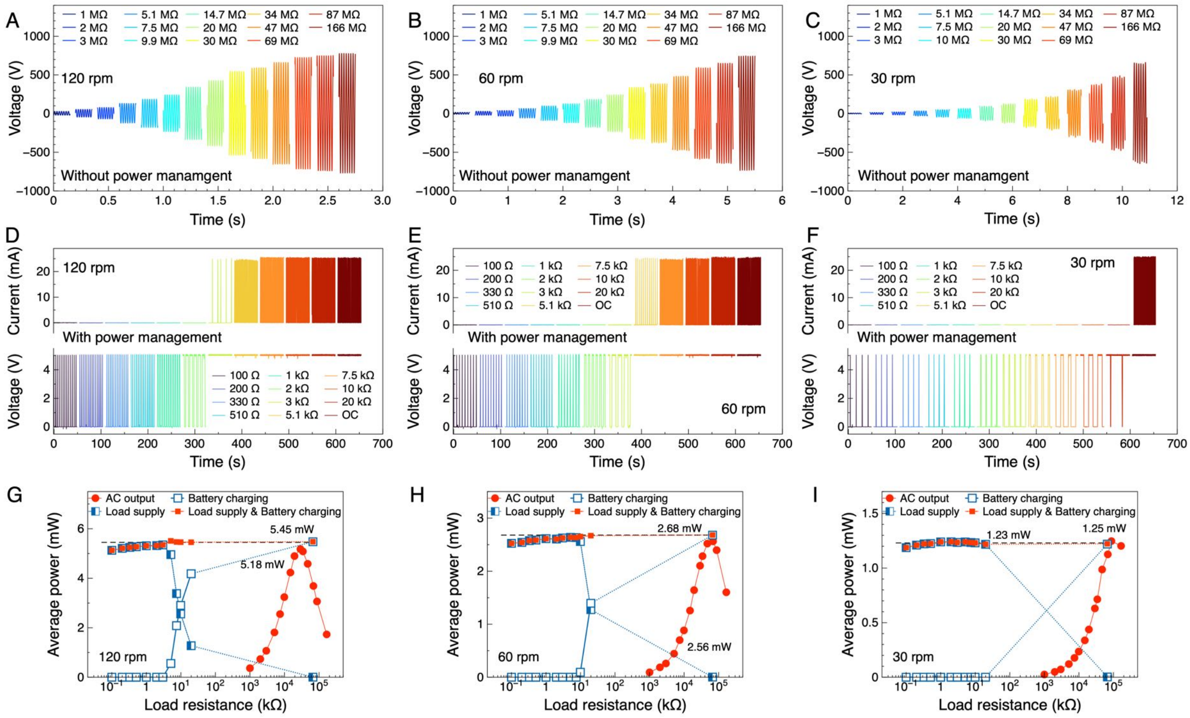

Supplementary Figure 53. Waveforms of (A–C) direct AC outputs without power management, (D–F) DC outputs with power management, and (G–I) average power comparisons with and without power management at rotation speeds of 120, 60, and 30 rpm.

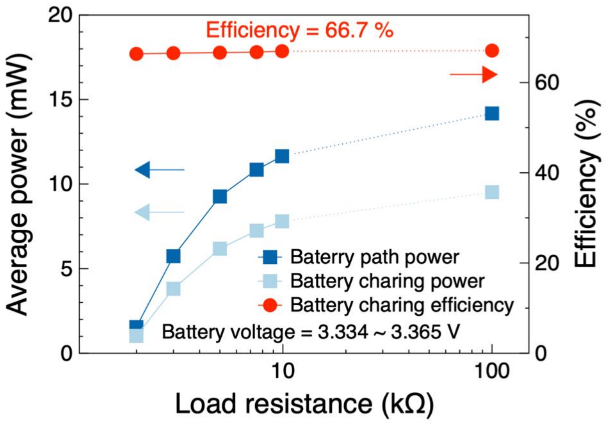

Supplementary Figure 54. Average powers of charging path ($P_{charge} = V_{reg} \times I_{bat}$) and charging battery ($P_{bat} = V_{bat} \times I_{bat}$), along with the battery charging efficiency ($\eta = P_{charge} / P_{bat}$).

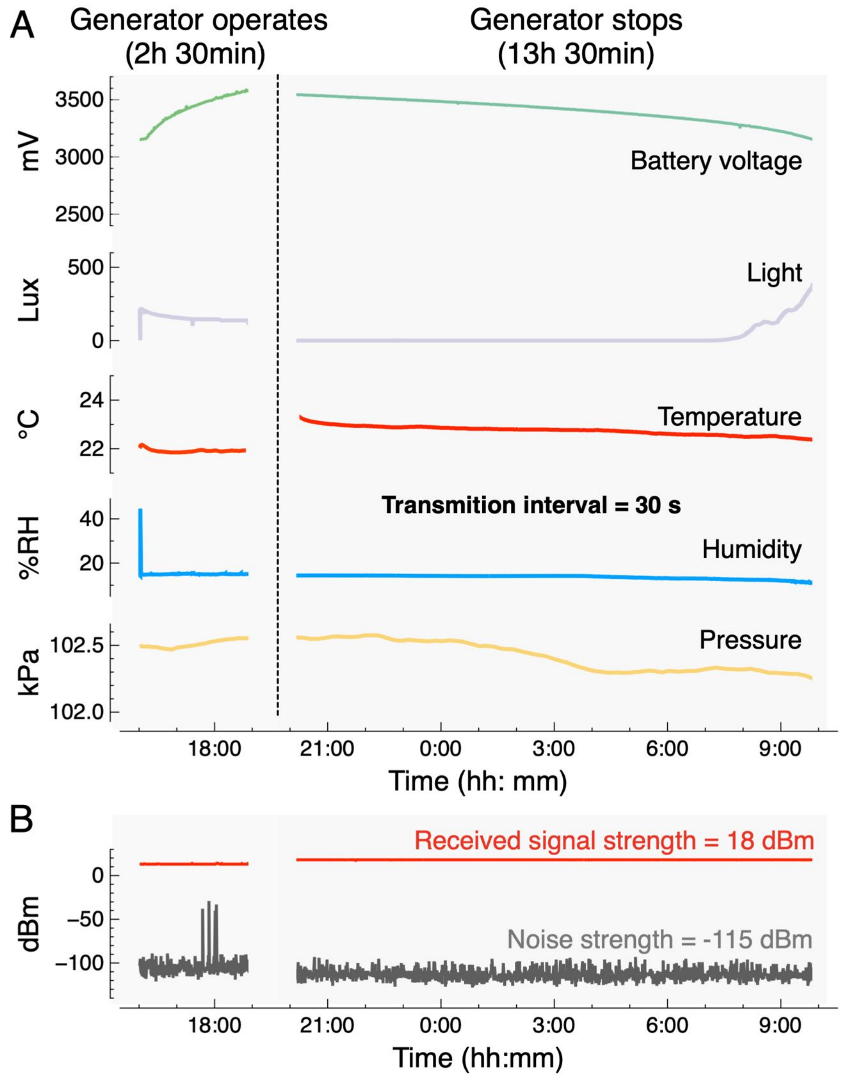

Supplementary Figure 55. (A) Sensing data from the LoRa sensing node. (B) Signal and noise strength at the receiving node.

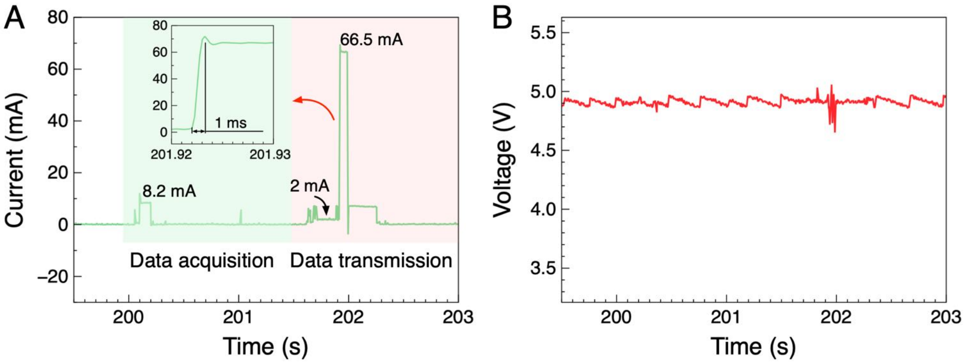

Supplementary Figure 56. (A) Supply current and (B) voltage of the LoRa node during operation.

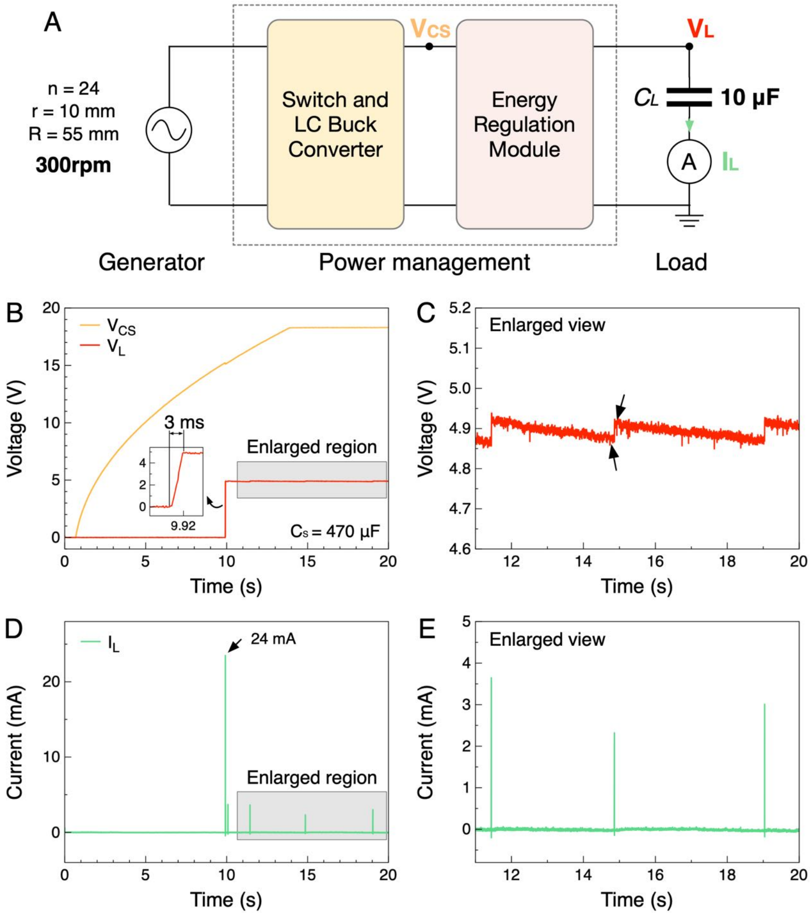

Supplementary Figure 57. Demonstration of the power management system driving a capacitive load (10 μF capacitor). (A) Schematic of the experimental setup. (B) Voltage after the LC buck converter (V_{CS}) and regulated DC output on the load (V_L). (C) Enlarged view of V_L. (D) Current through the load (I_L). (E) Enlarged view of I_L, with inset showing a further zoomed-in waveform.Here, we employ a 10 μF electrolytic capacitor as the load to evaluate the reliability of the proposed power management system under capacitive conditions (Supplementary Figure 57A). When the power management system starts supplying power, a current pulse of ~24 mA flows into the capacitor (Supplementary Figure 57D), charging it to the regulation voltage within 3 ms (Supplementary Figure 57B). The capacitor voltage is then maintained at the regulation point. A small periodic charge–discharge fluctuation (~50 mV) can still be observed (Supplementary Figure 57C), which results from the LTC3388's burst-mode operation: once the output voltage reaches the preset regulation level, the buck converter is disabled. As the capacitor voltage subsequently falls below the internal threshold due to capacitor leakage or the measurement current, the converter restarts and recharges the capacitor. At this moment, a charging current pulse appears (Supplementary Figure 57E).

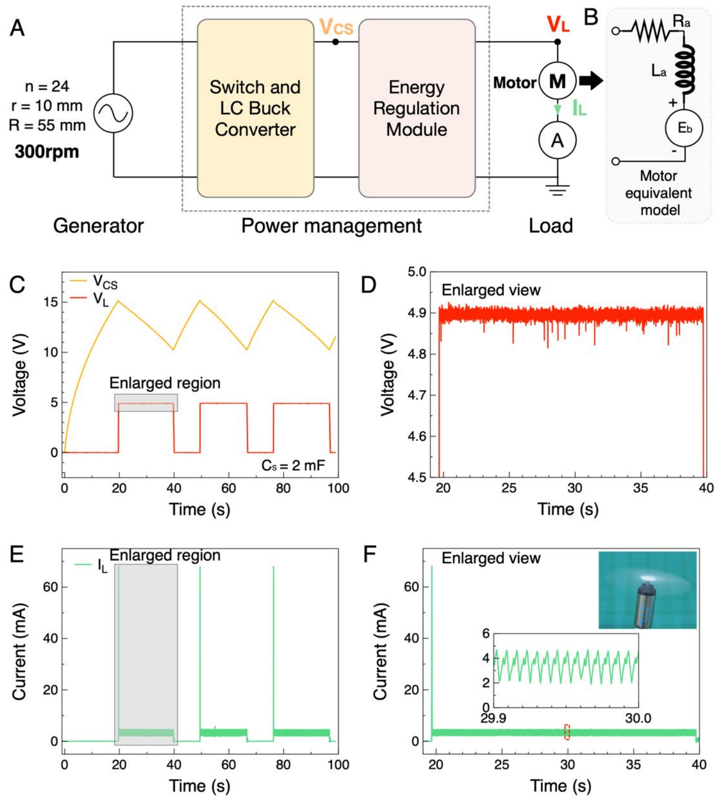

Supplementary Figure 58. Demonstration of the power management system driving an inductive load (DC motor). (A) Schematic of the experimental setup. (B) Simplified equivalent circuit model of the DC motor. (C) Voltage after the LC buck converter (V_{CS}) and regulated DC output on the load (V_L). (D) Enlarged view of V_L. (E) Current through the load (I_L). (F) Enlarged view of I_L, with inset showing a further zoomed-in waveform.

Here, a DC motor is employed as the load to evaluate the reliability of the proposed power management system under inductive conditions (Supplementary Figure 58A). Its simplified equivalent circuit model is shown in Supplementary Figure 58B, consisting of the armature resistance R_a, the armature inductance L_a, and the back electromotive force (EMF) E_b, which is proportional to the angular velocity. The motor used here is the 08G61-105C.3 from Portescap, with parameters R_a = 45.8 Ω, L_a = 271.6 μH, and a back-EMF constant of 0.53 V/1000 rpm. The testing results is shown in Supplementary Figures 58C–F, in which the backup battery is disabled.

The system operates in intermittent mode (Supplementary Figure 58C) because the motor's rated power exceeds the average power delivered by the generator after power management. At the beginning of each activation period, the motor starts from rest. Consequently, the current through the motor exhibits a transient surge (~68 mA, Supplementary Figure 58E), as the back electromotive force (EMF) is negligible at this stage. As the rotor accelerates, the increasing back-EMF reduces the net voltage across the winding, and the current decays to a steady value (~3.5 mA, Supplementary Figure 58F). In steady state, a small periodic ripple appears, mainly due to commutation and rotor-position-dependent variations in inductance and induced voltage. Nevertheless, throughout the entire cycle, the regulated output remains essentially constant

(Supplementary Figure 58D), demonstrating the robustness of the proposed power-management module.

Supplementary Note 1. Comparison of our power management system with previous studies

The detailed comparison of our power management system with previous studies is shown in Supplementary Table 2. These systems share a similar pipeline, including energy extraction, conversion, and utilization, but adopt different implementation methods. To highlight the advancement of our approach, we first compare the architectural differences and then discuss the detailed design considerations in our work that enable high efficiency. The details are elaborated below.

1. Energy extraction: Energy extraction is the first step for electrostatic and triboelectric generators, and several methods have been proposed: a) CMEO (cycles for maximum energy output): Prior study has shown that applying a synchronous switch to operate the generator in CMEO mode can maximize energy extraction, which we note is the maximum energy obtainable for a electrostatic and triboelectric generator.[23] b) MPPT (maximum power point tracking) is an alternative method that employs a bridge rectifier for energy extraction. While relatively straightforward to implement, in theory it can extract only up to 1/4 of the energy achievable with CMEO,[10] representing a considerable performance degradation. Moreover, the maximum power point is reached when the rectified voltage (V_{rec}) equals about half of the generator's open-circuit voltage (V_{OC}). For generators with kilovolt-level V_{OC}, this is still an extremely high voltage and poses a significant challenge for the design of the subsequent DC-DC converter. c) SSHI (synchronized switching harvesting on inductor) is a switch-based method that rapidly flips the voltage polarity of the generator through an LC loop, thereby reducing energy loss and increasing the harvested energy. Its efficiency depends on V_{rec} / $V_{\mathrm{OC}}\cdot\beta$,[10] where V_{rec}, V_{OC}, and β denote the rectified voltage, the generator's open-circuit voltage, and the voltage-flip efficiency, respectively. The maximum efficiency is achieved when V_{rec} approaches V_{OC} and β approaches 1, at which point the energy extracted per cycle equals that of CMEO. This strategy is well-suited for low-voltage sources such as piezoelectric generators, but it will pose significant challenges for the design of the subsequent DC-DC converter for high-voltage electrostatic and triboelectric generators. d) Bennet's doubler leverages an internal charge-pump mechanism to raise the storage voltage above the generator's instantaneous voltage, thereby forming a near-rectangular Q–V loop for higher input energy.[24] However, this strategy is only applicable to generators with intrinsic capacitance variation, and the capacitance ratio $\eta = C_{\mathrm{max}}/C_{\mathrm{min}}$ must exceed 2. If this condition is not met—for example, in our rotational freestanding electret generator—the pumping mechanism fails, and the effective gain approaches zero. e) Inductive transformer can efficiently step down high-voltage outputs. However, the generator impedance ($Z_{\mathrm{s}} = 1/2\pi fC$) decreases with frequency, whereas the transformer reactance ($X_{\mathrm{m}} = 2\pi fL$) increases with frequency. This opposite frequency dependence causes severe impedance mismatch across a broad frequency range, restricting the applicability of this approach to generators with narrow operating frequency range.

In summary, MPPT and SSHI methods suffer from low energy extraction efficiency and generate high DC voltages, which pose significant challenges for subsequent DC–DC converter design. Bennet's doubler is restricted to generators with intrinsic capacitance variation ($\eta > 2$), while inductive transformers are only efficient within a very narrow operating frequency range. By contrast, the CMEO method is simple, universal and achieves the maximum output,

requiring only a synchronous peak switch that closes at the generator's output peak. Thus, it is adopted in our work.

Notably, a variant of CMEO, termed CMEO with matching capacitor, is also use synchronous switch for energy extraction. This strategy is a compromise approach to mitigate the voltage mismatch between the generator and the switch, particularly when a threshold switch is used. As it introduces an additional parallel capacitance to the generator, its output is inherently lower than that of conventional CMEO.

2. Voltage/energy conversion: Since CMEO with a peak switch generates high-voltage pulses, a step-down voltage converter is required. Previous studies have demonstrated that passive LC buck or flyback converters can efficiently convert these pulses into low-voltage DC. In this work, we adopt a passive LC buck converter for simplicity. It is worth noting that the combination of a peak switch and an LC buck/flyback converter is sometimes referred as the synchronous electric charge extraction (SECE) strategy, which is essentially the same approach.

3. Energy regulation: After voltage/energy conversion, the high-voltage pulses are converted into low-voltage DC output. However, this DC output is not stable, and it varies with the generator's operating conditions and the load. The instability arises because the buck circuit operates at a frequency that is same to the pulse frequency after peak switch. Consequently, when the generator frequency shifts, the pulse frequency and the buck circuit frequency changes as well, leading to output fluctuations. This operating mode prevents the circuit from incorporating negative feedback or frequency-control mechanisms, thereby resulting in a load-dependent output. As a result, the output of the LC buck converter is inherently unstable and requires further regulation to be compatible with conventional electronics.

Here, we propose an energy regulation module that ensures a stable output by dynamically allocating harvested energy on demand. Unlike battery-based regulation, which stores all harvested energy before delivery and thus incurs higher losses and accelerates battery degradation, our strategy delivers energy directly to the load and stores only the surplus in the battery to improve overall efficiency. Furthermore, compared with topologies that rely solely on a single Zener regulator or even a more efficient DC–DC converter, our design leverages the battery as an energy buffer: it absorbs excess energy when available and supplies supplementary power when harvesting alone cannot meet the demand. Together, these features enable both stable operation and efficient utilization of harvested energy.

In summary, our power management system integrates three key modules: a peak switch that enables the generator to operate in CMEO mode and maximize energy extraction; an LC buck converter that performs the initial conversion of high-voltage pulses into low-voltage DC; and an energy regulation module that manages the harvested energy while delivering a stable output. To ensure high overall efficiency, each of these modules is carefully designed with several critical considerations, which are detailed below. a) Novel peak switch architecture: We propose a self-powered switch that requires only two MOSFETs and a few passive components, thereby eliminating the complicated controller used in traditional peak-switch architectures.[5,7,10]

This design simplifies fabrication, avoids the static power consumption inherent in the control logic of traditional peak-switches, and enables instant activation at the generator's voltage peak without any cold-start delay. In addition, the careful component selection allows the switch to be triggered by an extremely small charge, corresponding to an input capacitance as low as 10 pF. This minimizes the loading effect on the generator and significantly enhances the overall efficiency. b) LC buck converter optimization methodology: We systematically analyzed the factors influencing the efficiency of the LC buck converter in the context of electrostatic and triboelectric generator energy management, thereby establishing design guidelines for component selection and output regulation strategies to maximize conversion efficiency. **c)** Energy regulation module design: To improve conversion efficiency, the regulation module exploits the fact that an LC buck converter operates more efficiently at higher output voltages. A UVLO circuit is introduced to maintain the buck stage within a relatively high range (11–16 V), then release stored energy in discrete packets once a threshold is reached. This reduces switching losses and ensures consistent energy delivery per activation. Even under low-battery conditions, the UVLO thresholds and storage capacitance can be tuned to match the energy demands of specific tasks, such as a full sensing-and-transmission cycle of an IoT node. The module relies solely on threshold-based power-path control, avoiding complex algorithms and minimizing quiescent consumption. In addition, a high-efficiency DC–DC converter optimized for high-voltage input further enhances performance. Overall, the design achieves a high efficiency of 93% (P_{in} = 15.2 mW, P_{out} = 14.2 mW), even surpassing some integrated energy-regulation ICs[21–23] typically used with low-voltage energy harvesters such as piezoelectric, thermoelectric, and photovoltaic sources. All these factors combined ensure the remarkable power-management performance of this work.

Supplementary Note 2. Metrics for evaluating power management performance.

In this work, we evaluate power-management performance by the ratio of the final regulated output power to the original optimal direct AC output of the generator ($P_{reg}/P_{AC,opt}$). We choose this metric for two main reasons: (1) it is widely adopted in studies on high-voltage electrostatic and triboelectric energy harvesting, as $P_{AC,opt}$ serves as a figure of merit that quantifies the maximum output directly attainable on a resistive load from a generator; and (2) it reflects the overall effectiveness of the power-management system by accounting for both the energy-boosting effect and subsequent conversion efficiency, thereby providing a more realistic assessment of the circuit's performance.

Notably, we calculate the final output power based on the regulated output — in contrast to many previous power management studies that calculated it from an unregulated DC output. This distinction is important: the unregulated DC output depends strongly on the external load resistance and the operating conditions of the generator, making the reported performance highly condition-specific and less representative of practical scenarios where both load and generator conditions can vary. By contrast, power measured from the regulated output is stable, thereby providing a consistent and application-relevant measure of the actual usability of a power management system. However, few reported studies meet the stable-output criterion for benchmarking. Therefore, we extended the comparison presented in Supplementary Table 2 to include studies whose outputs were constrained within a defined voltage range (e.g., 2.8–4.2 V), as a practical approximation to the stable-output condition.

At the same time, we noticed that some studies, particularly for low-voltage generators (<300 V), use the maximum power after a full-wave bridge ($P_{FBR,opt}$, i.e., MPPT power) or the output power under CMEO conditions (P_{CMEO}, which can be easily measured in low-voltage systems) as a Supplementary Figure of merit for generator direct output and as the denominator for power-management performance metric. To enable a comprehensive comparison, we measured $P_{FBR,opt}$ and estimated P_{CMEO} using the relation $P_{CMEO} = 4\ P_{FBR,opt}$,[10] as shown in Supplementary Figures 2E and F. Based on this, we calculated the P_{reg} / P_{CMEO}, with the result listed in Supplementary Table 2. Additional metrics, such as P_{unreg} / P_{CMEO}, were also calculated to allow comparison with other studies from different perspectives, as shown in Supplementary Table 2.

Here, it should be noted that some out-of-plane (contact-separation mode) generators have asymmetric outputs, for which $P_{FBR,opt}$ can be much lower than 1/4 P_{CMEO}. Meanwhile, their output can be further enhanced using Bennet's doubler, owing to their capacitance-variation characteristics, as discussed in Supplementary Note 1. Consequently, sone power management performance metrics (e.g., $P_{unreg}/P_{FBR,opt}$) in such systems can have a much high value. These works are not directly comparable to the works of generators with symmetric outputs, such as ours.

The works that use P_{reg} / $P_{AC,opt}$ as the performance metric are summarized in Supplementary Table 1 for clearer comparison.

Supplementary Note 3. Input capacitance of the LC buck converter.

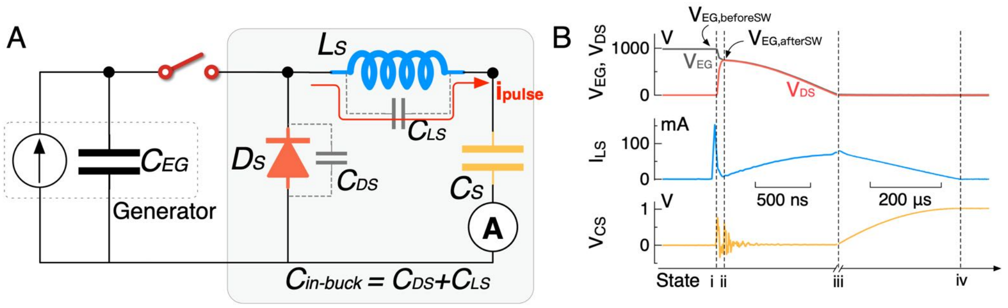

Supplementary Figure 59. (A) Schematic illustration of the input capacitance $C_{\mathrm{in,buck}}$. (B) Measured voltage and current waveforms during an energy transfer cycle.

The input capacitance of the buck converter ($C_{\mathrm{in,buck}}$) consists of two main components: 1) The junction (reverse) capacitance of the diode (C_{DS}), arising from its PN junction, and 2) the parasitic capacitance of the inductor (C_{LS}), originating from the coil structure. During switch closure, $C_{\mathrm{in,buck}}$ will cause a voltage drop at the generator's output. The higher the $C_{\mathrm{in,buck}}$, the larger the relative remaining voltage ($V'_{\mathrm{EG}}/V_{\mathrm{EG}}$) after switch closure, as shown below.

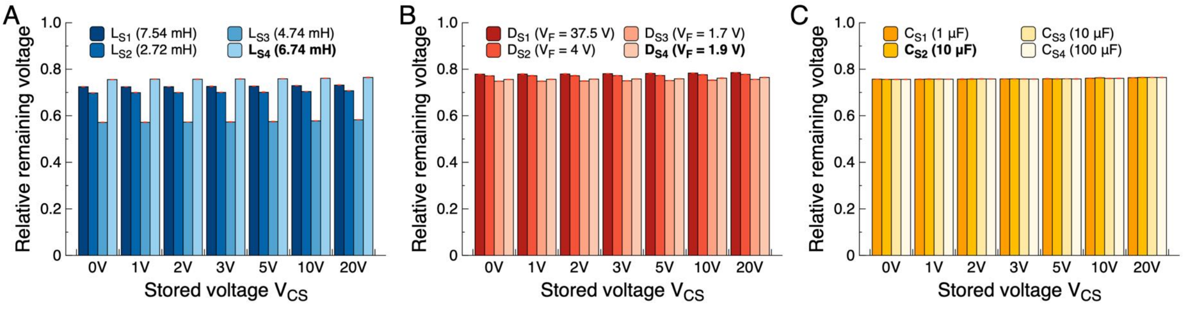

Supplementary Figure 60. Relative remaining voltage after switch closure with different (A) L_{S}, (B) D_{S}, and (C) C_{S}. Efficiency values were presented as the mean ± s.d. from n = 5 independent experiments.

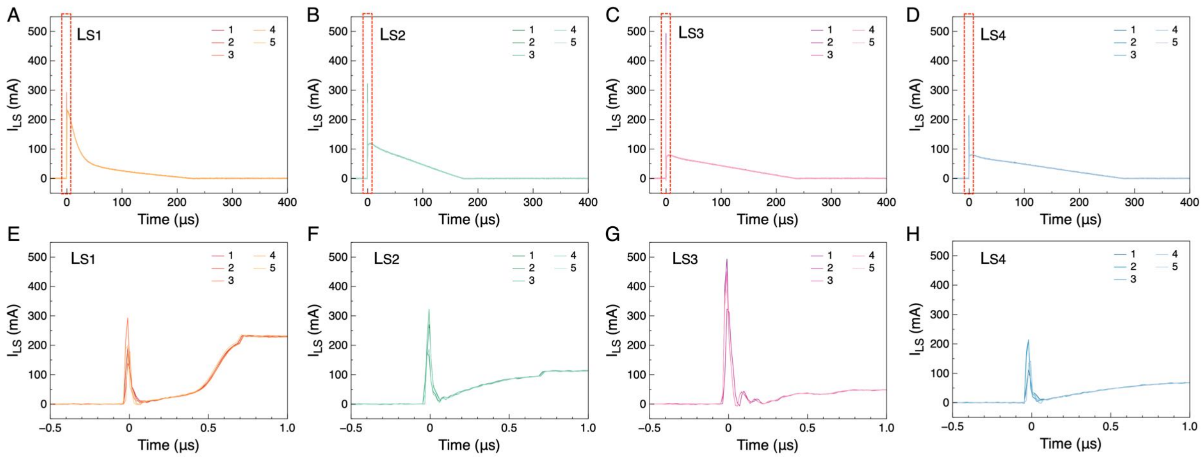

Supplementary Figure 61. Pulsed current through L_{S} during switch closure.

Notably, the parasitic capacitance C_{LS} of the inductor can introduce a pulsed current through L_{S} during switch closure, as illustrated in Supplementary Figure 61. This current is a displacement current flowing through C_{LS}, induced by the rapid voltage rise across the inductor (V_{DS}), and can be expressed as:

$$i_{pulse} = C_{LS}\frac{dV_{DS}}{dt} \tag{1}$$

Given that the switching time is as short as 30–50 ns, i_{pulse} can reach relatively high values. The larger the C_{LS}, the greater the resulting i_{pulse}. Additionally, this displacement current can cause a noticeable voltage oscillation on the measurement probe, primarily due to the lead inductance introduced by the current meter and voltage probe setup. It is important to note that this is merely a transient current flowing through the measurement lead inductance and does not affect the voltage across the storage capacitor (C_S).

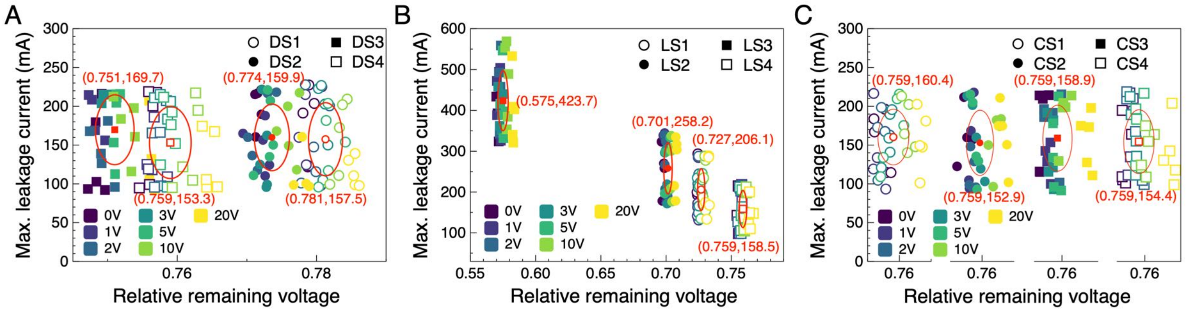

Supplementary Figure 62. Maximum pulsed current and relative remaining voltage during switch closure under various buck converter configurations and V_{CS} levels. The red point represents the average result for each configuration, while the major or minor axes of the ellipse indicate the standard deviations of the relative remaining voltage or the maximum pulsed current, respectively. Each configuration and V_{CS} condition was evaluated using n = 5 independent experiments. The red point indicates the mean value, and the horizontal and vertical axes of the ellipse represent the s.d. of the relative remaining voltage and the maximum pulsed current, respectively.

The relative remaining voltage and the pulsed voltage during switch closure across different buck configurations and V_{CS} values are summarized in Supplementary Figure 62. Replacing the diode (D_S) affects its reverse capacitance C_{DS}, thereby influencing the relative remaining voltage but without affecting the pulsed current. Replacing the inductor (L_S) changes its parasitic capacitance C_{LS}, which leads to observable changes in both the voltage drop and pulsed current. Since the storage capacitor (C_S) is well isolated by L_S, it does not influence either the voltage drop or the pulsed current. Notably, the parasitic parameters are intrinsic to the components themselves and remain unaffected by changes in V_{CS}.

Supplementary Note 4. Calculation method for the normalized energy at each state during the LC buck energy transfer process

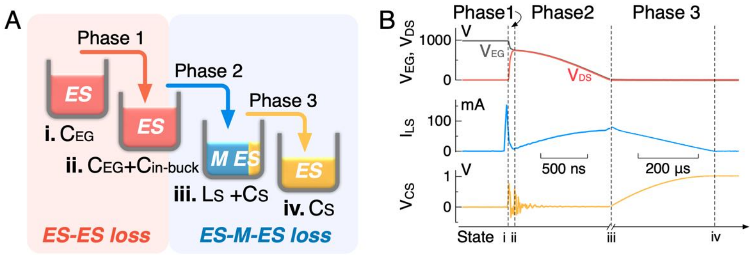

Supplementary Figure 63. (A) Schematic illustration of the three energy-transfer phases and the associated four states, and (B) the corresponding voltage and current waveforms within one energy-transfer cycle.

In State i, the energy is stored as electrostatic energy in the generator's capacitance C_{EG}. In the experiment setup, the input capacitance of the peak-detection switch (C_{in}) also contributes to the output; thus, the energy at this state is given by:

$$E_{\text{state,i}} = \frac{1}{2}(C_{\text{EG}} + C_{\text{in}}) \times V_{\text{EG}}^2 \quad (2)$$

In State ii, the energy redistributes across the combined capacitance of the generator and the input capacitor of the buck converter, $C_{in,buck}$. The energy at this state is:

$$E_{\text{state,ii}} = \frac{1}{2}\left(C_{\text{EG}} + C_{\text{in}} + C_{\text{in,buck}}\right) \times {V_{\text{EG}}'}^2 = E_{\text{state,i}} \times \frac{V_{\text{EG}}'}{V_{\text{EG}}} \quad (3)$$

In State iii, the energy is mostly stored as magnetic energy in the inductor L_S, and the rest as electrostatic energy in the storage capacitor C_S. The total energy at this state is:

$$E_{\text{state,iii}} = \frac{1}{2}L_{\text{S}} \times I_{\text{LS}}^2 + \frac{1}{2}C_{\text{S}} \times (V_{\text{state,iii}}^2 - V_{\text{state,i}}^2) \quad (4)$$

In State iv, all the energy is fully transferred to C_S. The energy at this state is:

$$E_{\text{state,iv}} = \frac{1}{2}C_{\text{S}} \times (V_{\text{state,iv}}^2 - V_{\text{state,i}}^2) \quad (5)$$

The normalized energy at each state is then obtained by dividing the energy at that state by the initial energy $E_{state,i}$.

Supplementary References

1. Wang, Z. *et al.* A universal self-triggered passive management strategy for enhancing the output power of triboelectric nanogenerators. *Energy Environ. Sci.* **18**, 3761–3772 (2025).

2. Wu, H. *et al.* Efficient energy conversion mechanism and energy storage strategy for triboelectric nanogenerators. *Nat. Commun.* **15**, 6558 (2024).

3. Dai, Y. *et al.* Effective charging of commercial lithium cell by triboelectric nanogenerator with ultrahigh voltage energy management. *Adv. Sci.* **11**, 2404253 (2024).

4. Luo, Y. *et al.* Advanced Energy harvesting from low-frequency ocean waves for lithium-ion battery applications. *Energy Environ. Sci.* **18**, 4821–4832 (2025).

5. Cheng, X. *et al.* High efficiency power management and charge boosting strategy for a triboelectric nanogenerator. *Nano Energy* **38**, 438–446 (2017).

6. Song, Y. *et al.* High-efficiency self-charging smart bracelet for portable electronics. *Nano Energy* **55**, 29–36 (2019).

7. Liu, Y., Shi, Z., Badel, A., Miyoshi, T. & Suzuki, Y. Self-powered synchronous electric charge extraction rectifier for rotational electret energy harvester with dual-stage electrodes. *IEEE Trans. Power Electron.* **38**, 13166–13180 (2023).

8. Liufu, Y. H., Dai, D. & Liu, Z. Triple-MOSFETs switch for adaptive maximum capacitance point tracking of triboelectric nanogenerators. *Nano Energy* **106**, 108042 (2023).

9. Chang, S. *et al.* Harvesting high entropy triboelectric energy using a universal synchronous switching unit for self-powered wireless sensing systems. *Nano Energy* **131**, 110271 (2024).

10. Zhen, D. *et al.* A triboelectric energy harvesting IC with high-voltage synchronous electric charge extraction strategy and superior systematic efficiency. *IEEE Trans. Circuits Syst. Regul. Pap.* 1–13 (2024) doi:10.1109/TCSI.2024.3405900.

11. Park, I. *et al.* A 4.5-to-16μW integrated triboelectric energy-harvesting system based on high-voltage dual-input buck converter with MPPT and 70V maximum input voltage. in *2018 IEEE International Solid - State Circuits Conference - (ISSCC)* 146–148 (IEEE, San Francisco, CA, 2018). doi:10.1109/isscc.2018.8310226.

12. Park, I., Maeng, J., Shim, M., Jeong, J. & Kim, C. A bidirectional high-voltage dual-input buck converter for triboelectric energy-harvesting interface achieving 70.72% end-to-end efficiency. in *2019 Symposium on VLSI Circuits* C326–C327 (IEEE, Kyoto, Japan, 2019). doi:10.23919/vlsic.2019.8778018.

13. Wang, Z. *et al.* Ultrahigh electricity generation from low-frequency mechanical energy by efficient energy management. *Joule* **5**, 441–455 (2021).

14. Wang, Z. *et al.* A universal self-triggered passive management strategy for enhancing the output power of triboelectric nanogenerators. *Energy Environ. Sci.* **18**, 3761–3772 (2025).

15. Tu, Y. *et al.* IC-compatible high-efficiency power management for triboelectric nanogenerators based on the concept of limit. *IEEE Trans. Power Electron.* **39**, 6–13 (2024).

16. Lee, J. *et al.* A triboelectric energy-harvesting interface with scalable multi-chip-stacked bias-flip and daisy-chained synchronous signaling techniques. *IEEE J. Solid-State Circuits* **57**, 3825–3839 (2022).

17. Lee, S.-H., Jeong, Y.-W., Park, S.-J. & Shin, S.-U. A rectifier-reusing bias-flip energy harvesting interface circuit with adaptively reconfigurable SC converter for wind-driven triboelectric nanogenerator. *IEEE Trans. Ind. Electron.* **70**, 8022–8031 (2023).

18. Liu, Y., Badel, A. & Suzuki, Y. Enhancing output power of rotational electret energy harvester by synchronized switch harvesting on inductor. *J. Intell. Mater. Syst. Struct.* **33**, 183–195 (2022).

19. Ghaffarinejad, A. *et al.* Bennet's doubler working as a power booster for triboelectric nanogenerators. *Electron. Lett.* **54**, 378–379 (2018).

20. Zhang, H., Lu, Y., Ghaffarinejad, A. & Basset, P. Progressive contact-separate triboelectric nanogenerator based on conductive polyurethane foam regulated with a Bennet doubler conditioning circuit. *Nano Energy* **51**, 10–18 (2018).

21. Hosseini, A., Badeli, A. S., Davari, M., Sheikhaei, S. & Gharehpetian, G. B. A novel, software-defined control method using sparsely activated microcontroller for low-power, multiple-input, single-inductor, multiple-output DC–DC converters to increase efficiency. *IEEE Trans. Ind. Electron.* **70**, 2959–2970 (2023).

22. Li, S., Liu, X. & Calhoun, B. H. A 32nA fully autonomous multi-input single-inductor multi-output energy-harvesting and power-management platform with 1.2×10^5 dynamic range, integrated MPPT, and multi-modal cold start-up. in *2022 IEEE International Solid-State Circuits Conference (ISSCC)* 1–3 (IEEE, San Francisco, CA, USA, 2022). doi:10.1109/ISSCC42614.2022.9731732.

23. Wang, J. *et al.* A cross regulation reduced multi-output and multi-VCR piezoelectric energy harvesting system using shared capacitors. in *2022 IEEE International Symposium on Circuits and Systems (ISCAS)* 2768–2772 (IEEE, Austin, TX, USA, 2022). doi:10.1109/ISCA48785.2022.9937938.

24. Duc Truong, B. & Phu Le, C. Theoretical analysis of electrostatic energy harvester conSupplementary Figured as Bennet's doubler based on Q-V cycles. *Int. J. Circuit Theory Appl.* **51**, 2518–2543 (2023).
