# Supplementary material for: Power management for triboelectric and electrostatic generators enabling continuous and stable power delivery
Source: Nat Commun. 2026 Apr 25;17:5704. doi: 10.1038/s41467-026-72323-w (PMC13320162; doi:10.1038/s41467-026-72323-w)
Supplement: Supplementary file 2 — Description of Additional Supplementary Files [file 41467_2026_72323_MOESM2_ESM.pdf]

Description of Additional Supplementary Files

Supplementary Movie 1: Demonstration of driving 40 parallel hygrothermographs at a rotation speed of 30 rpm.

Supplementary Movie 2: Demonstration of driving 7 wireless IoT sensors at a rotation speed of 30 rpm.

Supplementary Movie 3: Demonstration of a 20 dBm LoRa-based multi-meter monitoring node, battery charging, and battery-backed power supply.
