# Supplementary material for: Power management for triboelectric and electrostatic generators enabling continuous and stable power delivery
Source: Nat Commun. 2026 Apr 25;17:5704. doi: 10.1038/s41467-026-72323-w (PMC13320162; doi:10.1038/s41467-026-72323-w)
Supplement: Supplementary file 6 — Transparent Peer Review file [file 41467_2026_72323_MOESM6_ESM.pdf]

nature portfolio

Peer Review File

Power management for triboelectric and electrostatic generators enabling continuous and stable power delivery

Corresponding Author: Professor Xiongying Ye

This file contains all reviewer reports in order by version, followed by all author rebuttals in order by version.

Version 0:

Reviewer comments:

Reviewer #1

(Remarks to the Author)
This manuscript presents an innovative and comprehensive strategy for energy harvesting and management using triboelectric and electrostatic generators. The work introduces a self-powered peak-detection switch, an optimized LC buck converter, and a self-adaptive energy regulation module that jointly enhance energy conversion efficiency and enable real-world application. The experimental results are rigorous and cover diverse operating scenarios, demonstrating the feasibility and effectiveness of the proposed system.

The work is well-structured, data-rich, and technically sound. The experimental and theoretical analyses are thorough, and the figures and references support the narrative effectively.

Explicitly discuss how this affects low-voltage applications and whether this limits the switch's use with certain types of TENGs.

A more detailed quantitative comparison table between the proposed system and existing energy harvesting strategies (e.g., SSHI, Bennet doubler, Zener-regulated systems) would strengthen the manuscript's impact.

Include a brief discussion on miniaturization, manufacturability, and system integration challenges.

Line 21: "…can even surpass the generator's optimal AC output" → clarify or rephrase for accuracy.

Line 186: "efficiency improves slightly at higher voltages" → consider quantifying this.

Line 415: "ICharge is the current through the battery charging path" → define measurement accuracy/method if possible.

Reviewer #2

(Remarks to the Author)
This manuscript presents a triboelectric energy harvesting system featuring a peak detection mechanism and a voltage elevation strategy aimed at enhancing energy conversion efficiency. While the proposed system demonstrates a functional implementation, the overall novelty and contribution appear limited. Below are several specific comments for the authors to consider:

Comparison with Conventional Detection Circuits:
Since a key contribution of this work is the proposed peak detector, it is important to compare it with existing peak detection circuits. The manuscript lacks a detailed comparison—both conceptual and experimental—with conventional approaches. Furthermore, the authors should benchmark their work against prior triboelectric energy harvesting (TEG) power management circuits that have demonstrated high efficiency. Notable examples include:

Ref. 1: A Triboelectric Energy Harvesting IC With High-Voltage Synchronous Electric Charge Extraction Strategy and Superior Systematic Efficiency, IEEE TCAS-I.

Ref. 2: A 4.5-to-16μW Integrated Triboelectric Energy-Harvesting System Based on High-Voltage Dual-Input Buck Converter with MPPT and 70V Maximum Input Voltage, IEEE ISSCC.

Ref. 3: Power Management Technologies for Triboelectric Nanogenerators.

Including these comparisons would better contextualize the novelty and performance of the proposed circuit.

Architecture and Energy Loss Considerations:
Figure 1 depicts a multi-stage power management system including energy boosting, AC–DC conversion, and regulation stages. Such cascaded architectures are known to suffer from cumulative energy losses. This issue has been addressed in prior works via single-stage or hybrid architectures, which the authors do not discuss.

Energy Regulation Novelty:
Energy regulation is a well-established concept in energy harvesting systems. The novelty of the proposed regulation strategy is not clearly articulated. It is recommended that the authors benchmark their approach against prior works, particularly in the context of MISIMO (Multiple-Input Single-Inductor Multiple-Output) systems. Relevant references include:

Ref. 4: A Novel, Software-Defined Control Method Using Sparsely Activated Microcontroller for Low-Power, Multiple-Input, Single-Inductor, Multiple-Output DC–DC Converters to Increase Efficiency.

Ref. 5: A 32nA Fully Autonomous Multi-Input Single-Inductor Multi-Output Energy-Harvesting and Power-Management Platform with 1.2×10^5 Dynamic Range, Integrated MPPT, and Multi-Modal Cold Start-Up.

Ref. 6: A Cross Regulation Reduced Multi-Output and Multi-VCR Piezoelectric Energy Harvesting System Using Shared Capacitors.

A thorough comparison would help clarify the contributions of the proposed strategy in the context of existing literature.

System Efficiency:
The reported energy conversion efficiency of the proposed system is relatively low. This significantly limits its practical value, especially when compared to state-of-the-art designs. The authors should discuss this limitation explicitly and explore possible avenues for improvement.

Reviewer #3

(Remarks to the Author)
The authors present a universal and efficient power management strategy for triboelectric and electrostatic generators. The strategy includes a self-powered peak-detection switch, an LC buck converter, and an energy regulation module.Experimental results show that the managed DC output achieves power comparable to optimal AC output. It ensures a stable output for conventional electronics. Overall, I think this work bridges the gap between laboratory prototypes and practical applications. After careful evaluation, I suggest a minor revision with the following suggestions:
1. In the introduction section of the manuscript, the authors claim that the power management strategy proposed in this study is “universal” and features efficient energy utilization. However, the comparison with previous works is not adequately in-depth. Although the supplementary materials provide a comparison table (Table S1), only some parameters are listed. As a reviewer, I suggest that the authors conduct a thorough analysis in the text regarding why the power utilization ratio (Pend/PAC) in this study can reach 1.08, a value higher than those of other strategies documented in the literature. This analysis should be integrated with the information provided in the comparison table.
2. The performance of the self-powered peak detection switch is described in the manuscript, but a detailed discussion of the experimental parameter adjustment is missing. The supplementary material provides a large number of test results on switch performance under different conditions (such as Figures S5 to S14). I suggest adding a section in the main text to discuss how these experimental parameters (such as the values of CEG and VEG) affect the switch efficiency. Combined with these test data, a detailed analysis should be provided to explain how to optimize the switch performance by adjusting these parameters.
3. The energy regulation module is rather cursorily described in the paper. However, the supplementary material encompasses detailed schematics (Figure S48) and photographs (Figure S49) of the module. It is suggested that the authors incorporate this information from the supplementary material into the paper. Additionally, the authors should include a detailed explanation of the working principle of the energy regulation module and the functions of its various components.
4. The current experiments mainly focus on the output and charging power of the power management system under various conditions, yet the impact of load characteristics on system performance remains underexplored. It is recommended to further evaluate the system's performance when dealing with loads of different dynamic nature （such as fast - changing and pulsating loads） and types （purely resistive, inductive, and capacitive）. Measurements of key indicators like output voltage stability and power fluctuation range are essential for a more comprehensive assessment of the system's applicability and reliability.
5. In terms of reference citations for power management strategies, it's recommended to expand beyond the current references. Including studies on novel topological structures and intelligent control algorithms would enrich the research background and comparative analysis.

Reviewer #4

(Remarks to the Author)
Considering the importance of mechanical energy harvesters to power autonomous sensors, the authors propose a novel technique/strategy for the energy extraction. After reading the manuscript, the following doubts and questions have arisen:

1. Conceptually, the idea behind the proposed technique/strategy is not clearly explained. In other words, using as a reference the typical voltage-charge plot of a TENG, how is the cycle of energy output desired in the proposed circuit?

2. Also, conceptually, the idea reflected in Fig. 1A needs more explanation. How the power at the second stage (17.9 mW) can be higher than that in the first stage (13 mW)? Probably, the methodology to make the measurements explains this fact.

3. According to the circuit proposed in Fig. 2A, this is able to detect the positive peak, but do you also need to detect the negative peak? How many activations of the switch are required/desired per period? Of course, this is completely related with point #1. Depending on the desired V-Q plot, the number of activations will 1 or 2, but this is unclear in the current version of the manuscript.

4.One of the critical issues (probably the most critical) related to triboelectric nanogenerators is the time-dependent inherent capacitance. Such effects are not clearly evaluated in the manuscript. For example:
a) what is the relation required between the inherent TENG capacitance and the capacitances of the peak-detection switch (Fig. 2A) in order to guarantee an appropriate operating principle?
b) How the effects of the time-dependent TENG capacitance are considered in test platform shown in Fig. 2B?
It is true that the effects of different generator capacitances are shown in Figs. 2E, F, and G, but in TENGs the capacitance changes dynamically with time, and this is not reflected in the current version of manuscript.

Version 1:

Reviewer comments:

Reviewer #2

(Remarks to the Author)
Although the circuit-level contribution is limited, the overall system integration is possible to be considered for publication.

Reviewer #3

(Remarks to the Author)
The authors have addressed all the comments and carefully revised the manuscript. In my opinion, the revision can be accepted.

Reviewer #4

(Remarks to the Author)
After reading the rebuttal coming from the authors, this reviewer still has the following concerns (using the same enumeration employed in the previous round):

1. Several doubts arise from Fig. R8. For example:
a) Considering a resistive load is not the common case considered in the literature. Why do you assume that?
b) Why there are no negative voltages in Fig. R8F for the performance in red? Note that, in many cases, authors talk about a bipolar output of the generator, but according to that figure, the output is unipolar. In addition, this figure should be in the main text, not in the supplementary file.
c) Again for Fig. R8F, the performance without the switch (in orange) is unclear.

2. This reviewer understands the explanation provided by the authors, but in that case, the terminology and drawings employed in the manuscript should be modified. Talking about stages 1, 2, … seems that the circuit has different blocks in cascade, and then the numbers of power provided make no sense.

3. Now it is clear that there are two activations per cycle and the goal is to achieve the CMEO. In that case, before the peak, the TENG has to be in open circuit but, according to Fig. 2 A(i) there is a current path (highlighted in red). How do you explain that?

4. a) The answer provided is not convincing at all. First, it seems that no relationship is required, but then "it is still recommended that the generator capacitance be as large as possible compared to the capacitance of the switch, so as to reduce the loading effect and ensure efficiency". It is completely unclear. How much is the capacitance of the TENG? How much are the capacitances of the peak detector? How much is the reduction of the TENG voltage after introducing the peak detector?
b) The sentence "the switch can also operate with generators (e.g., CS-mode TENG) that have time-dependent capacitance" needs some extra evaluation depending on the answers provided in the previous point.

Version 2:

Reviewer comments:

Reviewer #4

(Remarks to the Author)
In the opinion of this reviewer, some of the answers provided are not convincing. For example:

1.Figure R1 from the response to reviewer file.
Case (E): is this the output of the generator or the signal applied to the load? Why this voltage is higher than the open-circuit voltage represented in Fig. C?
Case (F): even with the inclusion of the full-wave rectifier, the V-Q plot generally shows the performance of the TENG for the negative half cycle, but this is not reflected in your figure. And again, why this voltage is higher than the open-circuit voltage in Fig. C?

2.There is no significant improvement in Figure 1 to better show what was indicated in the comment of the previous round.

3.According to the equation provided in the response-to-reviewers file, it seems that the higher the value of CL, the better, since then V_out,CL is equal to V_out and we have the open-circuit conditions. But this makes no sense, CL should be as low as possible. Therefore, the derivation of this equation and/or the explanation around it are erroneous.

4.It seems that no new information has been included in the main manuscript regarding the effects and relation of the capacitances.

Version 3:

Reviewer comments:

Reviewer #4

(Remarks to the Author)
--

Reviewer #5

(Remarks to the Author)
This manuscript reports an energy management strategy for triboelectric or electrostatic generators based on a self-powered peak-tracking switch, demonstrating excellent energy management performance for devices operating in the independent triboelectric layer mode. The manuscript has undergone multiple rounds of revision and has reached a high-quality level. The authors' responses to the reviewers' comments, particularly those addressing the concerns of Reviewer #4, are accurate and appropriate. Regarding the energy management strategy proposed in this manuscript, I have only one concern: what are the key innovations or differences between the self-powered peak-tracking switch strategy presented here and the one described in this paper (Nano Energy 131 (2024) 110271)?

Open Access This Peer Review File is licensed under a Creative Commons Attribution 4.0 International License, which permits use, sharing, adaptation, distribution and reproduction in any medium or format, as long as you give appropriate credit to the original author(s) and the source, provide a link to the Creative Commons license, and indicate if changes were made.
In cases where reviewers are anonymous, credit should be given to 'Anonymous Referee' and the source.
The images or other third party material in this Peer Review File are included in the article's Creative Commons license, unless indicated otherwise in a credit line to the material. If material is not included in the article's Creative Commons license and your intended use is not permitted by statutory regulation or exceeds the permitted use, you will need to obtain permission directly from the copyright holder.
To view a copy of this license, visit https://creativecommons.org/licenses/by/4.0/

Point-to-Point Response to the Reviewer's Comments
(Comments in black, response in blue, revisions are highlighted)

Dear reviewers:

We sincerely thank you for your recognition of our work and for the detailed, insightful comments and suggestions, which have greatly helped us improve the quality of the manuscript. We have revised the manuscript accordingly and the detailed corrections are listed below point by point.

Reviewer #1 (Remarks to the Author):

This manuscript presents an innovative and comprehensive strategy for energy harvesting and management using triboelectric and electrostatic generators. The work introduces a self-powered peak-detection switch, an optimized LC buck converter, and a self-adaptive energy regulation module that jointly enhance energy conversion efficiency and enable real-world application. The experimental results are rigorous and cover diverse operating scenarios, demonstrating the feasibility and effectiveness of the proposed system. The work is well-structured, data-rich, and technically sound. The experimental and theoretical analyses are thorough, and the figures and references support the narrative effectively.

Answer: Thanks for your comments, and we are very grateful for your recognition of our work.

1. Explicitly discuss how this affects low-voltage applications and whether this limits the switch's use with certain types of TENGs.

Answer: Thanks for your comments and it's a good question.

#1 Regarding the low-voltage application: The switch in this work is primarily designed for TENGs with high output voltages (i.e., 500–1200 V, as shown in Fig. 2E). To extend its operating range to lower-voltage generators, two approaches can be considered:

a) Increasing C_{in}. A larger C_{in} allows sufficient charge to be injected into C_{Drive}, enabling the switch to operate even with a lower input voltage. We validated this concept in Fig. S22A (R1), which shows that the minimum operating voltage decreases as C_{in} increases.

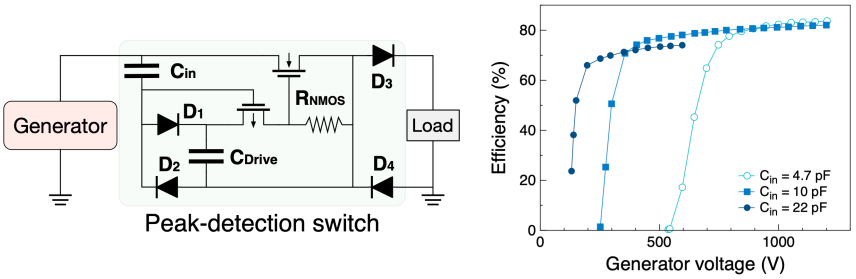

Figure R1 (S22A). Schematic of peak switch and the influence of C_{in} on switch efficiency with various generator voltages. (Voltage drop rate = 20 V ms^{-1}, R_L = 3 MΩ, C_{EG} = 120 pF, unless otherwise noted).

b) Removing the capacitor voltage divider. In this case, Q_{Drive} can be further increased, and the switch circuit is modified to the topology shown below, which can handle even lower input voltages while also simplifying the circuit.

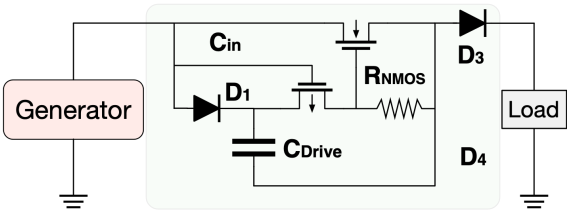

Figure R2 (S23). Switch design for low-voltage generator applications

Revision:

Line 201: Finally, the switch fails to operate below 300 V. Increasing C_{in} (Figure S22) or removing capacitor divider (Figure S23) can mitigate this trigger failure by enhancing the available driving energy with lower generator voltages.

#2 Regarding compatibility with different types of TENGs: The switch can be adapted for different types of TENGs by using switches with different operating voltage ranges. For example, for a contact–separation mode TENG with asymmetric output and a large positive–negative voltage difference, two switches—each tailored to the operating range of its respective polarity—can be combined with the topology shown in Fig. R3 (S34A) to realize a switch configuration for asymmetric voltages, as demonstrated below.

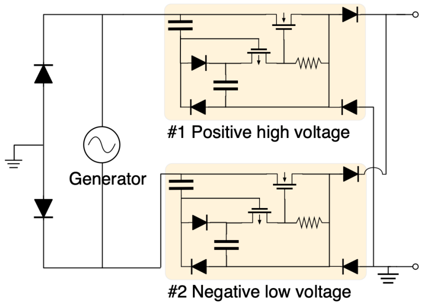

Figure R3 (S34A). Bipolar switch design for generators with asymmetric output.

Revision:

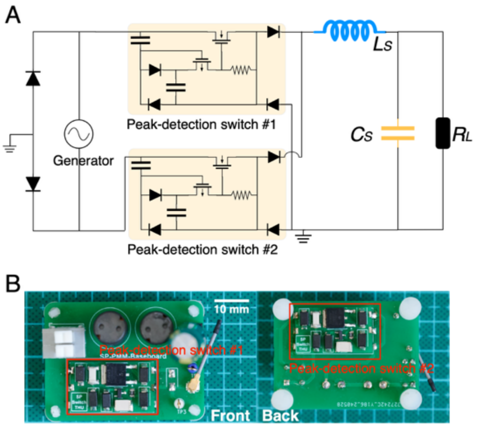

Figure S34. (A) Schematic and (B) photographs of the switches and LC buck converter used in the tests with rotational electret generators with bipolar output.

Note that this structure can also handle generators with asymmetric outputs (e.g., contact–separation mode TENGs) by adjusting each switch's operating voltage range to match the generator voltage of its respective polarity.

2. A more detailed quantitative comparison table between the proposed system and existing energy harvesting strategies (e.g., SSHI, Bennet doubler, Zener-regulated systems) would strengthen the manuscript's impact.

Answer: Thanks for your comments and kind suggestion. We have included representative works on SSHI and Bennet's doubler in Table S2 and added a detailed discussion in Notes S1 and S2 to highlight the differences between our work and these strategies, thereby clarifying the contribution of our study.

For Zener-regulated systems—due to their intrinsically low efficiency and their common use mainly for demonstration purposes—studies in the literature usually do not report efficiency values or any detailed data, and therefore these cases were not included Table S2 and only discussed in Note S1.

3. Include a brief discussion on miniaturization, manufacturability, and system integration challenges.

Answer: Thanks for your comments and kind suggestion. We have added a discussion in the Discussion section.

Revision:

Line 487: Looking ahead, the proposed power management system can be further miniaturized and integrated, broadening its applicability to portable, wearable, and other space-constrained devices. Achieving this goal requires overcoming several challenges. First, miniaturizing the peak-detection switch is challenging because reduced spacing increases parasitic capacitance, which can compromise switching reliability. This necessitates careful circuit layout and shielding. Second, the LC buck converter remains a major size bottleneck, with the inductor dominating the footprint. Techniques such as multiple-release energy extraction, where energy delivery is divided into smaller packets per cycle, probably could help reduce inductor size and enable greater integration. Third, the energy regulation module is still relatively large; integrating it into an application-specific integrated circuit (ASIC) could substantially shrink its footprint. This, however, would demand suitable high-voltage processes and optimized DC–DC conversion to handle the elevated input voltage while preserving efficiency. Addressing these challenges will be critical to advancing the current prototype into a compact, fully integrated solution for broad application.

4. Line 21: "…can even surpass the generator's optimal AC output" → clarify or rephrase for accuracy.

Answer: Thanks for your comments and kind suggestion. We have corrected the corresponding expression in the revision.

Revision:

Line 21: …reaches 1.08 times of the generator's optimal AC output.

5. Line 186: "efficiency improves slightly at higher voltages" → consider quantifying this.

Answer: Thanks for your comments and kind suggestion. We have corrected the corresponding expression in the revision.

Revision:

Line 196: This efficiency shows a modest increase with the generator voltage, rising by about 5 percent from 500 to 1200V.

6. Line 415: “ICharge is the current through the battery charging path” → define measurement accuracy/method if possible.

Answer: Thanks for your comments and kind suggestion. We have added the corresponding measurement methodology to the revised manuscript.

Revision:

Line 428: …I_{Charge} is the current through the battery charging path, measured using a Device Current Waveform Analyzer (see Experimental Section and Figure S50).

Line 514: The current was measured using a Device Current Waveform Analyzer (Keysight CX3322A) with a CX1101A probe (410 mΩ, 40nA-1A).

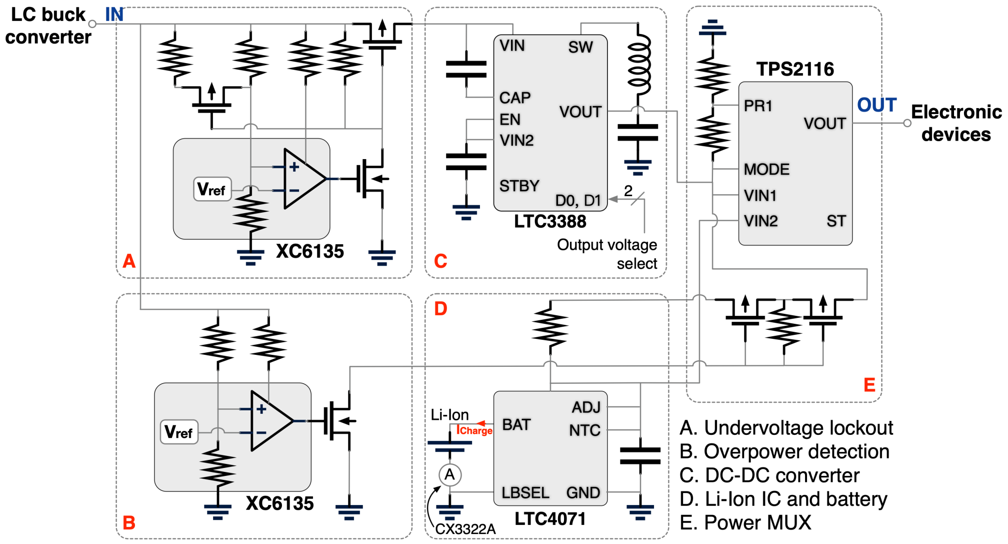

Figure S50. Schematic of the energy regulation module.

Reviewer #2 (Remarks to the Author):

This manuscript presents a triboelectric energy harvesting system featuring a peak detection mechanism and a voltage elevation strategy aimed at enhancing energy conversion efficiency. While the proposed system demonstrates a functional implementation, the overall novelty and contribution appear limited. Below are several specific comments for the authors to consider:

Answer: Thank you for your careful reading and constructive comments. We acknowledge the comparison in the original manuscript may be not sufficient, which as a result did not clearly highlight our novelty. We have accordingly revised the manuscript and have provided a more comprehensive comparison in Table S2 as well as in Notes S1 and S2, which better emphasizes the unique aspects of our work. Our detailed responses to your comments are provided below.

The newly added comparison table (Table S2) and the corresponding notes (Notes S1 and S2) are reproduced below as Table R1 and Notes R1 and R2 for ease of reference.

Table R1. Comparison of power management performance with reported works

References	Energy Source	Energy Extraction Strategy	Switch Type	Switch Architecture	Self-Powered/Cold Start	Static Power Consumption	Tolerated input voltage	Operation Frequency	Convertor Topology	Energy Regulation	Performance metric #1 $P_{reg}^{**}/P_{AC,opt}^{**}$	Performance metric #2 P_{reg}/P_{CMEO}^{**}	Other performance metric
Nano Energy (2017)[1]	Triboelectric (In-plane)	CMEO*	Peak	Discrete controller + low-side N-MOSFET	No	2.69 µW	<450 V	N/A	Flyback	No	N/A	N/A	$P_{unreg}^{**}/P_{AC,opt}^{**}$ 74.45% @ 4.47 V
Nano Energy (2019)[2]	Triboelectric (In-plane)	CMEO	Peak	Discrete controller + low-side N-MOSFET	No	2.69 µW	<305 V	N/A	LC buck	No	N/A	N/A	$P_{unreg}/P_{AC,opt}$ = 69.3% @ 1.7 V
IEEE Transactions on Power Electronics(2023)[3]	Electret (In-plane)	CMEO	Peak	Discrete controller + low-side N-MOSFET	Yes (Delayed start)	1~2 µW	<250 V	~300 Hz	LC buck	No	N/A	N/A	$P_{unreg}/P_{FBR+Buck}^{**}$ = 2.1 @ 4.6 V
Nano Energy (2023)[4]	Triboelectric (Out-of-plane)	CMEO with matching capacitor	Peak	Triple MOSFET Structure	Yes (Immediate start)	0	<300 V	1~3 Hz	LC buck	No	N/A	N/A	P_{unreg}/P_{in}^{*}= 79.3 % @ 6.2 V
IEEE Transactions on Circuits and Systems I[5] (Ref. 1)	Triboelectric (Out-of-plane)	CMEO	Peak	0.18 µm CMOS IC controller + low-side N-MOSFET	Yes (Delayed start)	1.56 µW	<300 V	1~5 Hz	Flyback	No	N/A	N/A	P_{unreg}/P_{CEMO} = 51.2% @ 7.56 V
ISSCC (2018)[6] (Ref. 2)	Triboelectric (Out-of-plane)	FBR*+MPPT*	Threshold	Embedded switch in 0.18 µm BCD* IC	Yes (Delayed start)	N/A	70 V	N/A	LC buck	No	N/A	N/A	P_{unreg}/P_{in} = 51.1 % @ 4.7 V
2019 Symposium on VLSI Circuits (2019)[7]	Triboelectric (N/A)	FBR+MPPT	Threshold	Embedded switch in 0.18 µm BCD IC	Yes (Delayed start)	361.6 nW	70 V	N/A	Dual input buck	N/A	N/A	N/A	P_{unreg}/P_{in} = 70.72% @ P_{in} = 20.7 µW
Joule (2019)[8]	Triboelectric (Out-of-plane)	CMEO	Threshold	Air-discharge switch	Yes (Immediate start)	0	7.5 kV	1~3 Hz	Flyback	No		N/A	$P_{unreg}/P_{AC,opt}$ 78.5% @ 15 V
Advanced Science (2024)[9]	Triboelectric (Out-of-plane)	CMEO with matching capacitor	Threshold	SCR-based switch	Yes (Immediate start)	0	1790 V (fixed)	N/A	LC buck	Battery (2.7-4.41 V)	$0.56\text{-}0.67^{*(1)}$ @ 2.7-4.4 V	N/A	N/A
Nature Communications (2024)[10]	Triboelectric (In-plane)	CMEO	Threshold	Needle-discharge switch	Yes (Immediate start)	0	~2500 V (fixed)	N/A	LC buck	DC-DC (1.8 V)	$0.40^{*(2)}$ @ 1.8 V	N/A	N/A
Energy Environ. Sci. (2025)[11]	Triboelectric (Out-of-plane)	CMEO with matching capacitor	Threshold	SCR-based switch	Yes (Immediate start)	0	N/A	N/A	LC buck	Hysteresis regulation (1.7-2.8 V)	$0.21^{*(3)}$ @ 1.7-2.8 V	N/A	N/A
Energy Environ. Sci. (2025)[12]	Triboelectric (In-plane)	N/A	N/A	N/A	N/A	N/A	N/A	~ 1364 Hz	Inductive transformer	Battery (2.8-4 V)	$0.63\text{-}0.91^{*(4)}$ @ 2.8-4 V	N/A	$P_{unreg}/P_{AC,opt}$ = 83.74% @ 3.21 V
IEEE Transactions on Power Electronics (2024)[13]	Triboelectric (Out-of-plane)	MR-SCE*	Peak	Discrete controller + MOSFETs	No	N/A	142 V	2 Hz	Switch capacitor convertor	No	N/A	N/A	P_{unreg}/P_{CMEO} 22.3 % @ 18 V
IEEE Journal of Solid-State Circuits (2022)[14]	Triboelectric (Out-of-plane)	SSHI*	Zero-Cross Detection	Embedded switch in 0.18 µm BCD IC	Yes (Delayed start)	N/A	<195 V	N/A	Switch Capacitor convertor	Battery (2.7-4.2 V)	N/A	$0.38^{*(5)}$ @ 3.6 V	P_{reg}/P_{in} = 70.7 % @ 3.6 V
IEEE Transactions on Industrial Electronics(2023)[15]	Triboelectric (Out-of-plane)	SSHI	Peak	0.18 µm BCD IC controller + low-side N-MOSFET	Yes (Delayed start)	N/A	<65 V	200~400 Hz	Switch Capacitor convertor	Battery (2.7-4.2 V)	N/A	$0.32^{*(6)}$ @ 3.6 V	P_{reg}/P_{in} = 79.3% @ 3.6 V

Journal of Intelligent Material Systems and Structures (2022)[16]	Electret (In-plane)	SSHI	Peak	Discrete controller with low-side MOSFET	Yes (Delayed start)	6 μW	<240 V	~109 Hz	N/A	No	N/A	N/A	$P_{unreg}/P_{FBR,\ opt}$ = 1.22 @ 3 V
Electronics Letters (2018)[17]	Triboelectric (Out-of-plane)	Bennet doubler	N/A	N/A	N/A	N/A	N/A	N/A	N/A	No	N/A	N/A	$P_{unreg}/P_{FBR,\ opt}$ 152.3 @ 256 V
Nano Energy (2018)[18]	Triboelectric (Out-of-plane)	Bennet doubler	N/A	N/A	N/A	N/A	N/A	N/A	N/A	No	N/A	N/A	$P_{unreg}/P_{FBR,\ opt}$ 2.5 @ 380 V
IEEE Transactions on Industrial Electronics (2023)[19] (Ref. 4)	Thermoelectric Photovoltaic Piezoelectric	MPPT	N/A	N/A	N/A	N/A	2.2-3.2 V 2.6-3.8 V 3-4.2 V	N/A	Discrete MISIMO* converter 7-19; 0-1.8; 2.2-4.2 V		N/A	N/A	P_{reg} / P_{in} = 92.5%
ISSCC (2022)[20] (Ref. 5)	Thermoelectric Photovoltaic Piezoelectric	MPPT SECE*	N/A	N/A	Yes (Delayed start)	N/A	<5 V	N/A	65nm CMOS MISIMO converter 0.6/1.2/3.3 V		N/A	N/A	P_{reg}/P_{in} = 80%
ISCAS (2022)[21] (Ref. 6)	Piezoelectric	SSHI	Zero-cross Detection	N/A	N/A	N/A	V_{PEH} = 1.7 V	250 Hz	0.18μm CMOS Switch capacitor DC-DC converter 0.5/1/2 V		N/A	N/A	P_{reg}/P_{in} = 0.894 (Simulation)
This work	Electret (In-plane)	CMEO	Peak	Positive-feedback-based discrete switch	Yes (Immediate start)	0	<1200 V	12 ~ 120 Hz	LC buck	DC–DC + Automatic Power Allocation (1.5/2.7/5 V)	1.08 @ 5 V	0.45	P_{unreg} /$_{PAC,opt}$ = 1.48 @401 V; $P_{unreg}/P_{FBR,opt}$** =2.4; P_{unreg}/P_{CMEO} =0.6; P_{reg}/P_{unreg} = 93.4% @ V_{unreg} = 12-18 V; P_{reg}/P_{sw}** = 79.3%; $P_{reg}/P_{FBR,opt}$ =1.78

*: CMEO (cycle for maximized energy output); FBR (Full-wave bridge rectifier); MPPT (Maximum power point tracking); MR-SCE (multi-release synchronized charge extraction); SSHI (synchronized switch harvesting on inductor); SECE (synchronous electric charge extraction); BCD (bipolar-CMOS-DMOS); MISIMO (multi-input single-inductor multi-output).

**: Power definitions: P_{reg} (average power with regulated DC output); $P_{AC,opt}$ (optimal average power with AC output); P_{unreg} (maximum average power with unregulated DC output); $P_{FBR+Buck}$ (average power with full-wave bridge rectifier and buck converter); P_{in} (input power to the power management system); $P_{FBR,\ opt}$ (optimal output power under full bridge rectification); P_{sw} (average power output with peak switch); P_{CMEO} (output power of generator operating in CMEO. For a generator with time-independent capacitance and symmetrical output, P_{CMEO} satisfies the relation $P_{CMEO} = 4\ P_{FBR,opt}$); $P_{chg,bat}$ (average battery-charging power).

*(#) Estimated values: (1) $P_{chg,bat}$ = 1.3 mW @ 2.7 V, $P_{chg,bat}$ = 1.56 mW @ 4.4V, $P_{AC,opt}$ = 2.32 mW; (2) P_{reg} = 6.9 mW @ 1.8 V, $P_{AC,opt}$ = 17.31 mW; (3) P_{reg} = 0.67 mW, $P_{AC,opt}$ = 3.1 mW; (4) $P_{chg,bat}$ = 42 mW @ 2.8 V, $P_{chg,bat}$ = 60 mW @ 2.8 V, $P_{AC,opt}$ = 66.14 mW; (5) P_{in} = 823.3 μW, P_{CMEO} = 4*$P_{FBR,opt}$ = 1540.8 μW, P_{reg} / P_{CMEO} = P_{reg} / P_{in} * P_{in}/P_{CMEO} = 70.7% * 823.3 / 1540.8;(6) V_{OC} = 150V, V_R = 60V, β = 1, P_{reg} / P_{CMEO} = P_{reg} / P_{in} * P_{in} / P_{CMEO} = 79.3% * 60 / 150.

Note R1 (S1). Comparison of our power management system with previous studies

The detailed comparison of our power management system with previous studies is shown in Table R1 (S2). These systems share a similar pipeline, including energy extraction, conversion, and utilization, but adopt different implementation methods. To highlight the advancement of our approach, we first compare the architectural differences and then discuss the detailed design considerations in our work that enable high efficiency. The details are elaborated below.

1. Energy extraction: Energy extraction is the first step for electrostatic and triboelectric generators, and several methods have been proposed: **a) CMEO** (cycles for maximum energy output): Prior study has shown that applying a synchronous switch to operate the generator in CMEO mode can maximize energy extraction, which we note is the maximum energy obtainable for a electrostatic and triboelectric generator.[22] **b) MPPT** (maximum power point tracking) is an alternative method that employs a bridge rectifier for energy extraction. While relatively straightforward to implement, in theory it can extract only up to 1/4 of the energy achievable with CMEO,[5] representing a considerable performance degradation. Moreover, the maximum power point is reached when the rectified voltage (V_{rec}) equals about half of the generator's open-circuit voltage (V_{OC}). For generators with kilovolt-level V_{OC}, this is still an extremely high voltage and poses a significant challenge for the design of the subsequent DC-DC converter. **c) SSHI** (synchronized switching harvesting on inductor) is a switch-based method that rapidly flips the voltage polarity of the generator through an LC loop, thereby reducing energy loss and increasing the harvested energy. Its efficiency depends on $\beta \cdot V_{rec}/V_{OC}$,[5] where V_{rec}, V_{OC}, and β denote the rectified voltage, the generator's open-circuit voltage, and the voltage-flip efficiency, respectively. The maximum efficiency is achieved when V_{rec} approaches V_{OC} and β approaches 1, at which point the energy extracted per cycle equals that of CMEO. This strategy is well-suited for low-voltage sources such as piezoelectric generators, but it will pose significant challenges for the design of the subsequent DC-DC converter for high-voltage electrostatic and triboelectric generators. **d) Bennet's doubler** leverages an internal charge-pump mechanism to raise the storage voltage above the generator's instantaneous voltage, thereby forming a near-rectangular Q–V loop for higher input energy.[22] However, this strategy is only applicable to generators with intrinsic capacitance variation, and the capacitance ratio $\eta = C_{max}/C_{min}$ must exceed 2. If this condition is not met—for example, in our rotational freestanding electret generator—the pumping mechanism fails, and the effective gain approaches zero. **e) Inductive transformer** can efficiently step down high-voltage outputs. However, the generator impedance ($Z_s = 1/2\pi fC$) decreases with frequency, whereas the transformer reactance ($X_m = 2\pi fL$) increases with frequency. This opposite frequency dependence causes severe impedance mismatch across a broad frequency range, restricting the applicability of this approach to generators with narrow operating frequency range.

In summary, MPPT and SSHI methods suffer from low energy extraction efficiency and generate high DC voltages, which pose significant challenges for subsequent DC–DC converter design. Bennet's doubler is restricted to generators with intrinsic capacitance variation ($\eta > 2$), while inductive transformers are only efficient within a very narrow operating frequency range. By contrast, the CMEO method is simple, universal and achieves the maximum output, requiring only a synchronous peak switch that closes at the generator's output peak. Thus, it is adopted in our work.

Notably, a variant of CMEO, termed CMEO with matching capacitor, is sometimes employed for energy extraction. This strategy is a compromise approach to mitigate the voltage mismatch between the generator and the switch, particularly when a threshold switch is used. As it introduces an additional parallel capacitance to the generator, its output is inherently lower than that of conventional CMEO.

2. Voltage/energy conversion: Since CMEO with a peak switch generates high-voltage pulses, a step-down voltage converter is required. Previous studies have demonstrated that passive LC buck or flyback converters can efficiently convert these pulses into low-voltage DC. In this work, we adopt a passive LC buck converter for simplicity. It is worth noting that the combination of a peak switch and an LC buck/flyback converter is sometimes referred as the synchronous electric charge extraction (SECE) strategy, which is essentially the same approach.

3. Energy regulation: After voltage/energy conversion, the high-voltage pulses are converted into low-voltage DC output. However, this DC output is not stable, and it varies with the generator's operating conditions and

the load. The instability arises because the buck circuit operates at a frequency that is same to the pulse frequency after peak switch. Consequently, when the generator frequency shifts, the pulse frequency and the buck circuit frequency changes as well, leading to output fluctuations. This operating mode prevents the circuit from incorporating negative feedback or frequency-control mechanisms, thereby resulting in a load-dependent output. As a result, the output of the LC buck converter is inherently unstable and requires further regulation to be compatible with conventional electronics.

Here, we propose an energy regulation module that ensures a stable output by dynamically allocating harvested energy on demand. Unlike battery-based regulation, which stores all harvested energy before delivery and thus incurs higher losses and accelerates battery degradation, our strategy delivers energy directly to the load and stores only the surplus in the battery to improve overall efficiency. Furthermore, compared with topologies that rely solely on a single Zener regulator or even a more efficient DC–DC converter, our design leverages the battery as an energy buffer: it stores excess energy when available and supplies supplementary power when harvesting alone cannot meet the demand. Together, these features enable both stable operation and efficient utilization of harvested energy.

In summary, our power management system integrates three key modules: a peak switch that enables the generator to operate in CMEO mode and maximize energy extraction; an LC buck converter that performs the initial conversion of high-voltage pulses into low-voltage DC; and an energy regulation module that manages the harvested energy while delivering a stable output. To ensure high overall efficiency, each of these modules is carefully designed with several critical considerations, which are detailed below. **a) Novel peak switch architecture**: We propose a self-powered switch that requires only two MOSFETs and a few passive components, thereby eliminating the complicated controller used in traditional peak-switch architectures.[1,3,5] This design simplifies fabrication, avoids the static power consumption inherent in the control logic, and enables instant activation at the generator's voltage peak without any cold-start delay. In addition, the careful component selection allows the switch to be triggered by an extremely small charge, corresponding to an input capacitance as low as 10 pF. This minimizes the loading effect on the generator and significantly enhances the overall efficiency. **b) LC buck converter optimization methodology**: We systematically analyzed the factors influencing the efficiency of the LC buck converter in the context of electrostatic and triboelectric generator energy management, thereby establishing design guidelines for component selection and output regulation strategies to maximize conversion efficiency. **c) Energy regulation module design**: To improve conversion efficiency, the energy regulation module exploits the fact that an LC buck converter operates more efficiently at higher output voltages. A UVLO circuit is introduced to maintain the buck stage within a relatively high range (11–16 V), then release stored energy in discrete packets once a threshold is reached. This reduces switching losses and ensures consistent energy delivery per activation. Even under low-battery conditions, the UVLO thresholds and storage capacitance can be tuned to match the energy demands of specific tasks, such as a full sensing-and-transmission cycle of an IoT node. The module relies solely on threshold-based power-path control, avoiding complex algorithms and minimizing quiescent consumption. In addition, a high-efficiency DC–DC converter optimized for high-voltage input further enhances performance. Overall, the design achieves a high efficiency of 93% (P_{in} = 15.2 mW, P_{out} = 14.2 mW), even surpassing some integrated energy-regulation ICs[19–21] typically used with low-voltage energy harvesters such as piezoelectric, thermoelectric, and photovoltaic sources. All these factors combined ensure the remarkable power-management performance of this work.

Note R2 (S2). Metrics for evaluating power management performance

In this work, we evaluate power-management performance by the ratio of the final regulated output power to the original optimal direct AC output of the generator (P_{reg} / $P_{AC,opt}$). We choose this metric for two main reasons: (1) it is widely adopted in studies on high-voltage electrostatic and triboelectric energy harvesting, as $P_{AC,opt}$ serves as a figure of merit that quantifies the maximum output directly attainable on a resistive load from a generator; and (2) it reflects the overall effectiveness of the power-management system by accounting for both the energy-boosting effect and subsequent conversion efficiency, thereby providing a more realistic assessment of the circuit's performance.

Notably, we calculate the final output power based on the regulated output — in contrast to many previous power management studies that calculated it from an unregulated DC output. This distinction is important: the unregulated DC output depends strongly on the external load resistance and the operating conditions of the generator, making the reported performance highly condition-specific and less representative of practical scenarios where both load and generator conditions can vary. By contrast, power measured from the regulated output is stable, thereby providing a consistent and application-relevant measure of the actual usability of a power management system. However, few reported studies meet the stable-output criterion for benchmarking. Therefore, we extended the comparison presented in Tables S2 (R1) to include studies whose outputs were constrained within a defined voltage range (e.g., 2.8–4.2 V), as a practical approximation to the stable-output condition.

At the same time, we noticed that some studies, particularly for low-voltage generators (<300 V), use the maximum power after a full-wave bridge ($P_{FBR,opt}$, i.e., MPPT power) or the output power under CMEO conditions (P_{CMEO}, which can be easily measured in low-voltage systems) as a figure of merit for generator direct output and as the denominator for power-management metrics. To enable a comprehensive comparison, we measured $P_{FBR,opt}$ and obtained P_{CMEO} using the relation $P_{CMEO} = 4\ P_{FBR,opt}$,[5] as shown in Figures S2E and F. Based on this, we calculated the P_{reg} / P_{CMEO}, with the result listed in Table S2. Additional metrics, such as P_{unreg} / P_{CMEO}, were also calculated to allow comparison with other studies from different perspectives, as shown in Table S2 (R2).

Here, it should be noted that some out-of-plane (contact-separation mode) generators have asymmetric outputs, for which $P_{FBR,opt}$ can be much lower than 1/4 P_{CMEO}. Meanwhile, their output can be further enhanced using Bennet's doubler, owing to their capacitance-variation characteristics, as discussed in Note R1 (S1). Consequently, power management performance metrics (e.g., P_{unreg} / $P_{FBR,opt}$) in such systems can have a much high value. These works are not directly comparable to the works of generators with symmetric outputs, such as ours.

The works that use P_{reg} / $P_{AC,opt}$ as the performance metric are summarized in Table S1 for clearer comparison.

1. Comparison with Conventional Detection Circuits:
Since a key contribution of this work is the proposed peak detector, it is important to compare it with existing peak detection circuits. The manuscript lacks a detailed comparison—both conceptual and experimental—with conventional approaches. Furthermore, the authors should benchmark their work against prior triboelectric energy harvesting (TEG) power management circuits that have demonstrated high efficiency. Notable examples include:
Ref. 1: A Triboelectric Energy Harvesting IC With High-Voltage Synchronous Electric Charge Extraction Strategy and Superior Systematic Efficiency, IEEE TCAS-I.
Ref. 2: A 4.5-to-16μW Integrated Triboelectric Energy-Harvesting System Based on High-Voltage Dual-Input Buck Converter with MPPT and 70V Maximum Input Voltage, IEEE ISSCC.
Ref. 3: Power Management Technologies for Triboelectric Nanogenerators.
Including these comparisons would better contextualize the novelty and performance of the proposed circuit.

Answer: Thank you for your comments and kind suggestion. We have added a detailed comparison between our peak-detection switch and those reported in previous studies, along with benchmarking against a greater number of representative prior power-management works, presented as Table R1 (Table S2 in the revised Supplementary Information).

1. Switch novelty: According to the comparison in Table R1, our switch shows several clear advancements over previous designs.

From the conceptual perspective: 1) The proposed peak-detection switch consists of only a few passive components and employs a positive-feedback mechanism to achieve reliable, fast peak detection and switching. This is fundamentally different from conventional peak switches, which usually rely on an additional controller to drive the main MOSFET[1,3,5]. 2) Our simple structure makes the switch easy to fabricate entirely from several off-the-shelf components, avoiding the cost and complexity of a custom ASIC switch control system.

From the performance perspective: 3) The proposed switch design removes the need for a separate power-supply rail for logic control, which not only eliminates static power consumption, but also enables instant start-up without cold-start delay. 4) In combination with high-voltage component selection and our unique topology, the proposed self-powered switch can directly accommodate kilovolt-level inputs—far exceeding the <300 V limits reported in prior peak-switch designs. 5) Furthermore, the working frequency range of 12–120 Hz, spanning an order of magnitude, also outperforms those of previous designs.

Since most of the previous works did not report switch efficiency, a direct efficiency comparison at the switch level was not possible. We therefore benchmarked the system-level performance, as shown in Table R1 (S2).

2. Benchmark of the power management circuits: In addition to the references you kindly mentioned, we further expanded Table R1 (S1) to include more representative power-management circuits employing diverse strategies, allowing for a more comprehensive comparison. Here, we use P_{reg} / $P_{AC,opt}$ as the performance metric, where P_{reg} is the stabilized DC load power after power management and $P_{AC,opt}$ is the generator's optimal AC output without power management. This metric is widely used in evaluating the performance of a power management system in the context of high-voltage electrostatic/triboelectric generator, and the rationale for using this metric is discussed in detail in our response to Comment #5 and Note R2 (S2).

Here, it should be noted that not all prior works provide a stabilized output and report P_{reg} / $P_{AC,opt}$, and some of them use totally different metrics for power management evaluation. This makes the direct comparison of our work with those works difficult. Nevertheless, to provide a comprehensive comparison, we list those metrics in Table R1 (S2) and, where possible, calculated the same metrics from our data according to their definitions.

Besides Table R1 (S2), we also discussed the power management topology in comparison with previous works in detail, and outlined the design considerations adopted in this work to enhance performance. This discussion has been added as Note R1 (S1).

2. Architecture and Energy Loss Considerations:
Figure 1 depicts a multi-stage power management system including energy boosting, AC–DC conversion, and regulation stages. Such cascaded architectures are known to suffer from cumulative energy losses. This issue has been addressed in prior works via single-stage or hybrid architectures, which the authors do not discuss.

Answer: Thank you for your comments. The main reason for adopting a multi-stage power-management architecture, rather than a single stage, is that the combination of the generator's kilovolt-level output and the need for event(peak)-triggered switching to maximize energy extraction makes a stable, load-compatible single-stage conversion impractical.

Specifically, unlike single-stage or hybrid architectures that generally extract and convert energy from low-voltage, low-impedance sources (typically < 20 V, e.g., thermoelectric, photovoltaic, or piezoelectric generators), triboelectric and electrostatic generators produce much higher AC output voltages of hundreds to thousands of volts, while delivering low output currents and exhibiting high impedance. Previous studies have shown that, to maximize energy extraction from such generators, they must operate in the cycle of maximized energy output (CMEO), which requires a switch to close synchronously at each generator voltage peak. This synchronous peak-switching produces high-voltage pulses at the generator's operating frequency, which in turn sets the operating frequency of the downstream LC buck converter. As this frequency varies with the generator's operating frequency, the converter's output voltage and ripple frequency also fluctuate. This operating mode prevents the circuit from incorporating negative feedback or frequency-control mechanisms, thereby resulting in a load-dependent output. Such variability prevents a single-stage architecture from delivering a stable output and causes both voltage and power to depend on the load. Therefore, a secondary down-conversion and regulation stage is required to ensure stable voltage and reliable power delivery.

We discussed and thought there may be two possible approaches for realizing a single-stage architecture: 1) One is to rectify the generator's output onto a capacitor, apply maximum power point tracking (MPPT) to optimize energy harvesting, and then use a single architecture to produce a stable output. However, MPPT can get at most 25% of the CMEO energy,[5] resulting in a significant reduction in total harvested energy. Moreover, achieving MPPT requires the storage capacitor voltage to be about half of the open-circuit voltage (V_{OC}), which can still be very high (hundreds of volts), posing challenges for efficient DC–DC conversion to electronics-compatible voltage levels. In addition, variations in V_{OC} due to operating conditions or intrinsic asymmetric outputs of the generator (e.g., contact–separation mode) may also increase the complexity and reduce the reliability of the MPPT strategy, limiting its general applicability. 2) The other approach is a multi-step energy extraction strategy, in which the switch threshold is set low to discharge the generator in many small voltage steps—for example, sixty 20 V pulses from a 1200 V. While this approach can enhance DC–DC conversion efficiency per transfer and ensure stable conversion, it significantly reduces the total amount of extracted energy: because stored energy scales with the square of voltage, a single 1200 V discharge delivers 72 μJ (100 pF source), whereas sixty 20 V discharges yield only 1.2 μJ, resulting in an order-of-magnitude loss.

Thus, for power management of high-voltage electrostatic/triboelectric generators, a three-stage architecture is necessary: the first stage extracts energy from the generator as discrete pulses, the second stage converts these kilo-volts pulses into an intermediate DC level, and the third stage regulates this intermediate voltage to a stable, electronics-compatible output. In our implementation, we achieve ~84.9% efficiency when converting a kilovolt-level pulse input into a stable 5 V output ($\eta = P_{reg} / P_{pulse}$ = 15.2 mW / 17.9 mW). Additionally, it is worth noting that the combination of a peak switch and an LC buck/flyback

converter is sometimes referred to as the synchronous electric charge extraction (SECE) strategy in the literature. Under this definition, our work can also be regarded as a two-stage strategy.

3. Energy Regulation Novelty:
Energy regulation is a well-established concept in energy harvesting systems. The novelty of the proposed regulation strategy is not clearly articulated. It is recommended that the authors benchmark their approach against prior works, particularly in the context of MISIMO (Multiple-Input Single-Inductor Multiple-Output) systems. Relevant references include:
Ref. 4: A Novel, Software-Defined Control Method Using Sparsely Activated Microcontroller for Low-Power, Multiple-Input, Single-Inductor, Multiple-Output DC–DC Converters to Increase Efficiency.
Ref. 5: A 32nA Fully Autonomous Multi-Input Single-Inductor Multi-Output Energy-Harvesting and Power-Management Platform with 1.2×10^5 Dynamic Range, Integrated MPPT, and Multi-Modal Cold Start-Up.
Ref. 6: A Cross Regulation Reduced Multi-Output and Multi-VCR Piezoelectric Energy Harvesting System Using Shared Capacitors.
A thorough comparison would help clarify the contributions of the proposed strategy in the context of existing literature.

Answer: Thank you for your comments and kind suggestion. We note that well-designed MISIMO energy-regulation modules have been reported [Ref. 4–6] and that related commercial ICs exist (e.g., BQ25570 from Texas Instruments). We have added them in the comparison Table. R2 (S1). However, these solutions primarily target low-voltage energy harvesters—such as thermoelectric, photovoltaic, and conventional piezoelectric generator—operating at a few volts. By contrast, electrostatic/triboelectric generators operate at hundreds to thousands of volts, for which efficient regulation remains limited. Thus, unlike the mentioned regulation modules that directly handle generator's low voltage outputs, our regulation module is a downstream stage co-designed with the high-voltage peak switch + LC-buck front end to handle the kilovolt-level outputs of electrostatic/triboelectric generators. This architecture entails a different set of design targets and considerations than those of prior energy regulation modules.

1) To improve the overall efficiency of the power-management and to handle the intermittency and instability of real-world electrostatic/triboelectric generator outputs, the input stage of the proposed regulation module incorporates a custom UVLO module. This UVLO module keeps the buck stage operating at a relatively high output voltage (11~16V) to maintain conversion efficiency, and releases stored energy in a discrete packet once a set energy threshold is met, thereby minimizing switching losses from frequent source toggling. Crucially, it ensures that each activation delivers a consistent amount of energy. Even with a depleted battery, the UVLO thresholds or storage capacitance can be tuned to match the energy needs of a specific task—such as a full sensing-and-transmission cycle of an IoT node—guarantee proper load functionality even under random, intermittent energy input.

2) Beyond the UVLO stage, the remaining high input voltage poses a challenge for achieving high-efficiency DC–DC conversion while delivering load-compatible voltages. To address this, we employ a commercial DC–DC converter (LTC3388) designed for high-voltage inputs and featuring a self-adjusting buck switching strategy—similar to the variable-frequency switching method in the mentioned works[19,20]—to optimize efficiency for low-power scenario. This approach significantly improves the end-to-end efficiency from the LC-buck stage to the final load. Under our test conditions, the proposed energy regulation module achieved an efficiency of 93% ($\eta = P_{reg} / P_{buck}$ = 14.2 mW / 15.2 mW), even a little higher than that in the mentioned works for low voltage energy harvesters (Ref. 4-6).[19–21]

3) Notably, the proposed energy regulation module relies only on threshold-based control for power-path selection, without relying on complex control algorithms. This approach enables automatic energy allocation with minimal control overhead, resulting in low quiescent power consumption. Moreover, its

modular architecture provides high flexibility, allowing users to assemble customized, high-performance energy regulation systems from off-the-shelf components to suit specific applications—thereby broadening the design's applicability.

In summary, from a system perspective, the proposed regulation module and the co-designed switch + LC-buck converter form the overall energy-regulation system for high-voltage electrostatic/triboelectric generator applications. It is specifically tailored to address the unique challenges of managing kilovolt-level, high-impedance, and intermittent electrostatic/triboelectric generator inputs, while reliably delivering power to both the load and the battery. The current design is validated through a discrete-component prototype, demonstrating its feasibility. Looking ahead, a dedicated application specific integrated circuit (ASIC) could further reduce size, enhance performance, and provide a compact, high-efficiency solution.

System Efficiency:

4. The reported energy conversion efficiency of the proposed system is relatively low. This significantly limits its practical value, especially when compared to state-of-the-art designs. The authors should discuss this limitation explicitly and explore possible avenues for improvement.

Answer: Thank you for your comments. We are sorry that the possibly unclear definition and explanation of the performance metrics in the original manuscript may have caused a misunderstanding of the performance of this work. To clarify this point, we have added a detailed explanation of the performance metric definition in Note S2 (R2).

Specifically, in this work, we evaluate power-management performance using the ratio of the final regulated output power to the original optimal direct AC output of the generator (P_{reg} / $P_{AC,opt}$) as the performance metric. We choose this metric for two main reasons: (1) it is widely adopted in studies on high-voltage electrostatic and triboelectric energy harvesting, as $P_{AC,opt}$ serves as a figure of merit that quantifies the maximum output directly attainable on a resistive load from a generator; and (2) it reflects the overall effectiveness of the power-management system by accounting for both the energy-boosting effect and subsequent conversion efficiency, thereby providing a more realistic assessment of the circuit's performance.

Notably, we calculate the final output power based on the regulated output — in contrast to many previous power management studies that calculated it from an unregulated DC output. This distinction is important: the unregulated DC output depends strongly on the external load resistance and the operating conditions of the generator, making the reported performance highly condition-specific and less representative of practical scenarios where both load and generator conditions can vary. By contrast, power measured from the regulated output is stable, thereby providing a consistent and application-relevant measure of the actual usability of a power management system. However, few reported studies meet the stable-output criterion for benchmarking. Therefore, we extended the comparison presented in Tables S2 (R1) and S1 to include studies whose outputs were constrained within a defined voltage range (e.g., 2.8–4.2 V), as a practical approximation to the stable-output condition. Within this broader set of works, our power management system achieves superior performance.

At the same time, we noticed that some studies, particularly for low-voltage generators (<300 V), use the maximum power after a full-wave bridge ($P_{FBR,opt}$, i.e., MPPT power) or the output power under CMEO conditions (P_{CMEO}, which can be easily measured in low-voltage systems) as a figure of merit for generator direct output and as the denominator for power-management metrics. To enable a comprehensive comparison, we also measured $P_{FBR,opt}$ and estimated P_{CMEO} using the relation $P_{CMEO} = 4 P_{FBR,opt}$, as shown in the newly added subfigures (Figs. S2E and S2F). Based on these values ($P_{FBR,opt}$ = 7.96 mW, P_{CMEO} = 31.8 mW), we derived the corresponding power-management metrics and added them to Table S2 (R2). For the regulated output, the ratios P_{reg} / $P_{FBR,opt}$ and P_{reg} / P_{CMEO} are 1.78 and 0.45, respectively. For the unregulated output, the values are 2.4 and 0.6. These results are superior to those reported in previous

works. Moreover, additional metrics such as P_{unreg} / $P_{AC,opt}$, were also calculated to enable comparison from different perspectives, as shown in Table S2 (R2).

Here, it should be noted that some out-of-plane (contact-separation mode) generators have asymmetric outputs, for which $P_{FBR,opt}$ can be much lower than 1/4 P_{CMEO}. Meanwhile, their output can be further enhanced using Bennet's doubler, owing to their capacitance-variation characteristics, as discussed in Note R1 (S1). Consequently, power management metrics (e.g., P_{unreg} / $P_{FBR,opt}$) in such systems can have a much high value. These works are not directly comparable to the works of generators with symmetric outputs, such as ours.

Reviewer #3 (Remarks to the Author):
The authors present a universal and efficient power management strategy for triboelectric and electrostatic generators. The strategy includes a self-powered peak-detection switch, an LC buck converter, and an energy regulation module. Experimental results show that the managed DC output achieves power comparable to optimal AC output. It ensures a stable output for conventional electronics. Overall, I think this work bridges the gap between laboratory prototypes and practical applications. After careful evaluation, I suggest a minor revision with the following suggestions:

Answer: Thanks for your comment, and we are grateful for your recognition of our work.

1. In the introduction section of the manuscript, the authors claim that the power management strategy proposed in this study is "universal" and features efficient energy utilization. However, the comparison with previous works is not adequately in-depth. Although the supplementary materials provide a comparison table (Table S1), only some parameters are listed. As a reviewer, I suggest that the authors conduct a thorough analysis in the text regarding why the power utilization ratio (Pend/PAC) in this study can reach 1.08, a value higher than those of other strategies documented in the literature. This analysis should be integrated with the information provided in the comparison table.

Answer: Thank you for your comment and kind suggestion. We have added a more detailed comparison table (Table S2) with additional representative works to enable a comprehensive comparison and highlight the advancement of our work. Furthermore, to explicitly demonstrate the remarkable performance of our design, we have included a thorough analysis of the key considerations for achieving high efficiency in our power management system in Note S1.

Revision: Table S2 and Note S1 in the revised Supplemental Information.

2. The performance of the self-powered peak detection switch is described in the manuscript, but a detailed discussion of the experimental parameter adjustment is missing. The supplementary material provides a large number of test results on switch performance under different conditions (such as Figures S5 to S14). I suggest adding a section in the main text to discuss how these experimental parameters (such as the values of CEG and VEG) affect the switch efficiency. Combined with these test data, a detailed analysis should be provided to explain how to optimize the switch performance by adjusting these parameters.

Answer: Thank you for your comment and kind suggestion. In the original manuscript, we discussed the influence of V_{EG}, C_{EG}, dV/dt, and R_L on switch efficiency, as well as possible ways to broaden switch adaptability by adjusting switch parameters such as C_{in}, C_{Drive} and R_{NMOS}. However, this part may not have been expressed clearly. In the revision, we clarified the discussion with consistent symbolic notation and made stronger links to the related measurements in the Supplementary Figures, so that the effect of these parameters and the optimization strategy are now more explicit.

Revision:
Lines 185 to 218 in the revised manuscript.

3. The energy regulation module is rather cursorily described in the paper. However, the supplementary material encompasses detailed schematics (Figure S48) and photographs (Figure S49) of the module. It is suggested that the authors incorporate this information from the supplementary material into the paper. Additionally, the authors should include a detailed explanation of the working principle of the energy regulation module and the functions of its various components.

Answer: Thanks for your comment and kind suggestion. After careful consideration, we decided to retain

the original functional-module-level description of the working principle for energy-regulation modules, in order to maintain clarity for general readers and keep the main text concise. However, we do agree that providing component-level explanations for each functional module would benefit readers seeking more technical detail, so these have been added to the Supplementary Information below Fig. S50.

Revision:

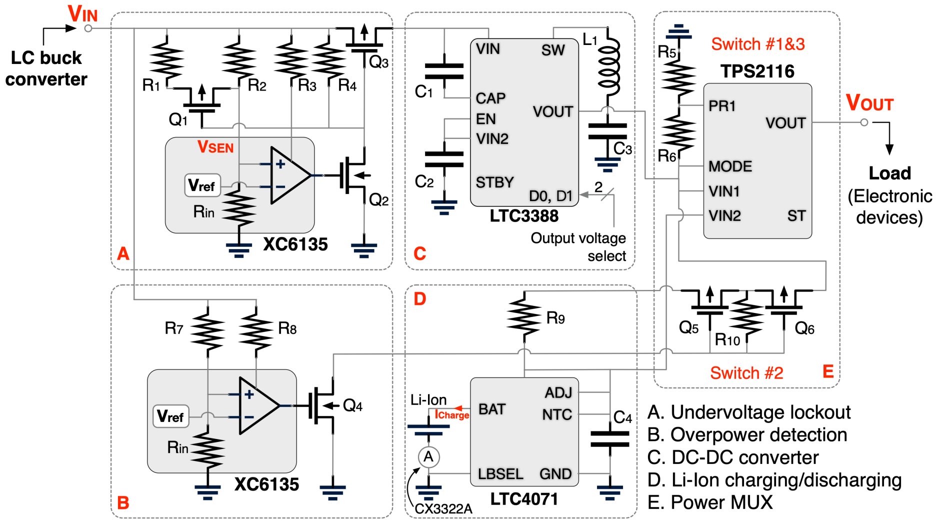

Figure R4 (S50). Schematic of the energy regulation module.

Note: Description of functional blocks for energy regulation module

A) Undervoltage lockout (UVLO) module: The UVLO module is implemented using a voltage detector IC (XC6135), which outputs a high level when its sensed voltage (V_{SEN}) is higher than the internal reference voltage (V_{ref}). Initially, the input voltage of the energy regulation module (V_{IN}) is zero and all MOSFETs (Q_1, Q_2, and Q_3) are in the cut-off state. As the input voltage increases, the voltage applied to the XC6135 (V_{SEN}) rises, and when $V_{SEN} > V_{ref}$, the XC6135 outputs a high level, turning Q_2 and Q_3 on—i.e., the UVLO is activated. The corresponding conduction threshold (upper threshold) can be expressed as:

$$V_{\text{TH,upper}} = V_{\text{ref}} \frac{R_{\text{in}} + R_2}{R_{\text{in}}}$$

where R_{in} is the input resistance of the XC6135. We note that when Q_2 conducts, Q_1 is also turned on, which makes V_{SEN} governed simultaneously by R_1 and R_2. As a result, the UVLO remains active until the input voltage V_{IN} drops below

$$V_{\text{TH,lower}} = V_{\text{ref}} \frac{R_{\text{in}} + (R_2 \parallel R_1)}{R_{\text{in}}}$$

Here, R_3 is used to limit the supply current to protect the XC6135 at high V_{IN}, and R_4 helps ensure reliable cut-off when the XC6135 output is low.

B) Overpower detection module: the overpower detection is realized by voltage detection circuit based on XC6135. The principle is straightforward: when the input power exceeds the load consumption, the input voltage V_{IN} (i.e., the voltage across the LC buck capacitor) increases continuously; conversely, when the input power is lower than the load consumption, V_{IN} decreases continuously. Thus, we set its threshold slightly higher (~0.1 V) than the upper threshold of the UVLO module. Once V_{IN} exceeds this threshold, it indicates that the input power is greater than the load consumption, thereby enabling switch #2 in the Power MUX module to divert the excess energy into the battery.

C) DC-DC converter: The design of the DC-DC converter is based on the LTC3388 family and follows the

standard application circuit provided in its datasheet. Two variants of the device are available: LTC3388-1, which allows output voltages of 1.2V, 1.5V, 1.8V, or 2.5V, and LTC3388-3, which allows 2.8V, 3.0V, 3.3V, or 5V. The output voltage is selected via the settings of pins D_0 and D_1.

D) Li-Ion charging/discharging module: A Li-ion charging/discharging circuit based on the LTC4071 is designed following the standard application circuit in the datasheet, providing protection against battery overcharge and overdischarge. The charging current is set by the resistor R_9. A Device Current Waveform Analyzer (Keysight, CX3322A) with CX1101A probe is employed to monitor the charging and discharging current of the battery.

E) Power multiplexer (MUX) module: The power MUX consists of three switches. Switches #1 and #3 are realized using TPS2116, configured in automatic priority mode, giving priority to V_{IN1} and switching to V_{IN2} when V_{IN1} drops. Thus, when the DC-DC converter (V_{IN1}) provides a valid output, it is connected to the load, while the battery (V_{IN2}) automatically takes over if the converter cannot deliver power due to excessive load demand or insufficient harvested energy. Switch #2 consists of two back-to-back P-MOSFETs (Q_5, Q_6) and is solely controlled by the overpower detection module, enabling excess harvested energy to be diverted to the battery.

4. The current experiments mainly focus on the output and charging power of the power management system under various conditions, yet the impact of load characteristics on system performance remains underexplored. It is recommended to further evaluate the system's performance when dealing with loads of different dynamic nature（such as fast - changing and pulsating loads）and types（purely resistive, inductive , and capacitive）. Measurements of key indicators like output voltage stability and power fluctuation range are essential for a more comprehensive assessment of the system's applicability and reliability.

Answer: Thanks for your comments and kind suggestion.

1) Regarding the system’s performance with dynamically changing loads, the demonstration using a LoRa node is a representative example. We have added the measured current and voltage profile during its operation as Fig. S56 (R5). When the node transmitting data at 20 dBm, the current rapidly increases from 2mA to 66.5 mA in 1 ms. Despite this large transient, the output voltage remains stable with only a small fluctuation, demonstrating the system’s strong adaptability to dynamic loads.

2) The output on resistive loads is shown in Figs. 5C and S52–53. In the revision, we added performance evaluations of the power management system with capacitive (10 μF electrolytic capacitor) and inductive (DC motor) loads, as presented in Figs. S57 (R6) and S58 (R7). In both cases, the output voltage remained stable, further confirming the robustness of the design under different load conditions.

Revision:

Line 465: Notably, the LoRa node is a dynamically varying load, whose supply current can surge from 2 mA to 66.5 mA within 1 ms during transmission (Figure S56). Even under this condition, the output of our management system remains stable, demonstrating robust performance. We further tested our power management system with both capacitive (Figure S57) and inductive and loads (Figure S58), confirming its ability to maintain stable operation under different load conditions.

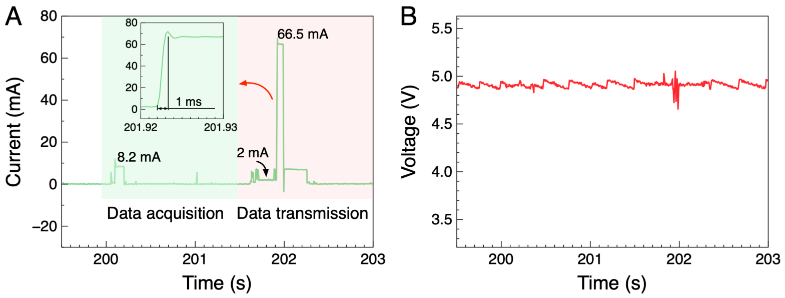

Figure R5 (S56). (A) Supply current and (B) voltage of the LoRa node during operation.

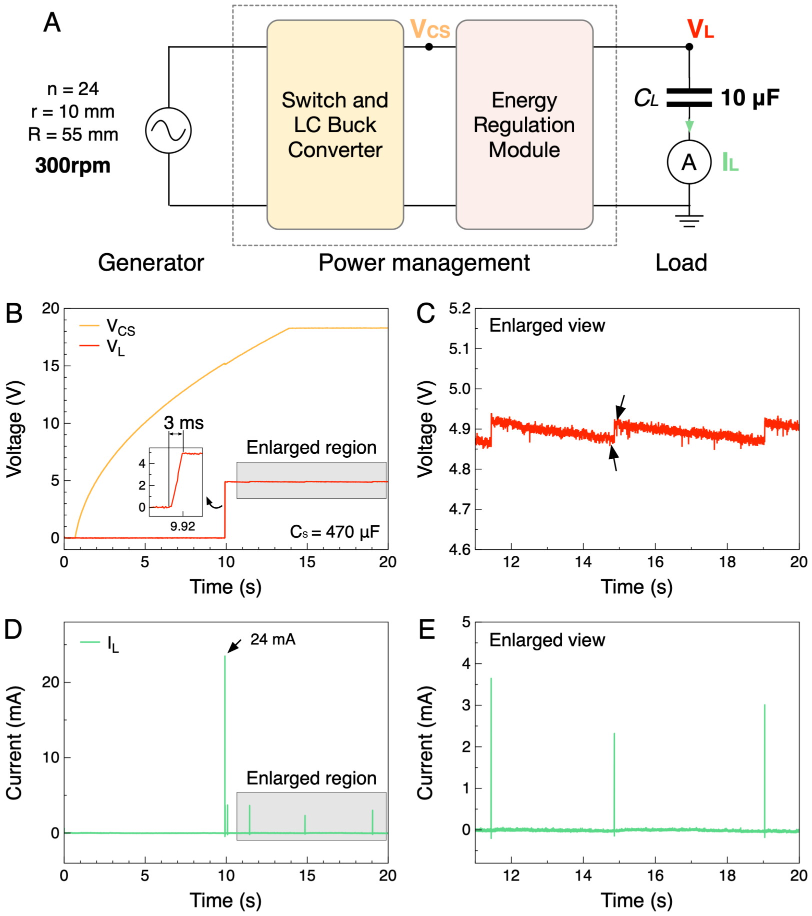

Figure R6 (S57). Demonstration of the power management system driving a capacitive load (10 μF capacitor). (A) Schematic of the experimental setup. (B) Voltage after the LC buck converter (V_{CS}) and regulated DC output on the load (V_L). (C) Enlarged view of V_L. (D) Current through the load (I_L). (E) Enlarged view of I_L, with inset showing a further zoomed-in waveform.

Here, we employ a 10 μF electrolytic capacitor as the load to evaluate the reliability of the proposed power management system under capacitive conditions (Figure S57A). When the system starts operating, a current pulse of ~24 mA flows into the capacitor (Figure S57D), charging it to the regulation voltage within 3 ms (Figure S57B). The capacitor voltage is then maintained at the regulation point. A small periodic charge–discharge fluctuation (~50 mV) can still be observed (Figure S57C), which results from the LTC3388's burst-mode operation: once the output voltage reaches the preset regulation level, the buck converter is disabled. As the capacitor voltage subsequently falls below the internal threshold due to capacitor leakage or the measurement current, the converter restarts and recharges the capacitor. At this moment, a charging current pulse appears (Figure S57E).

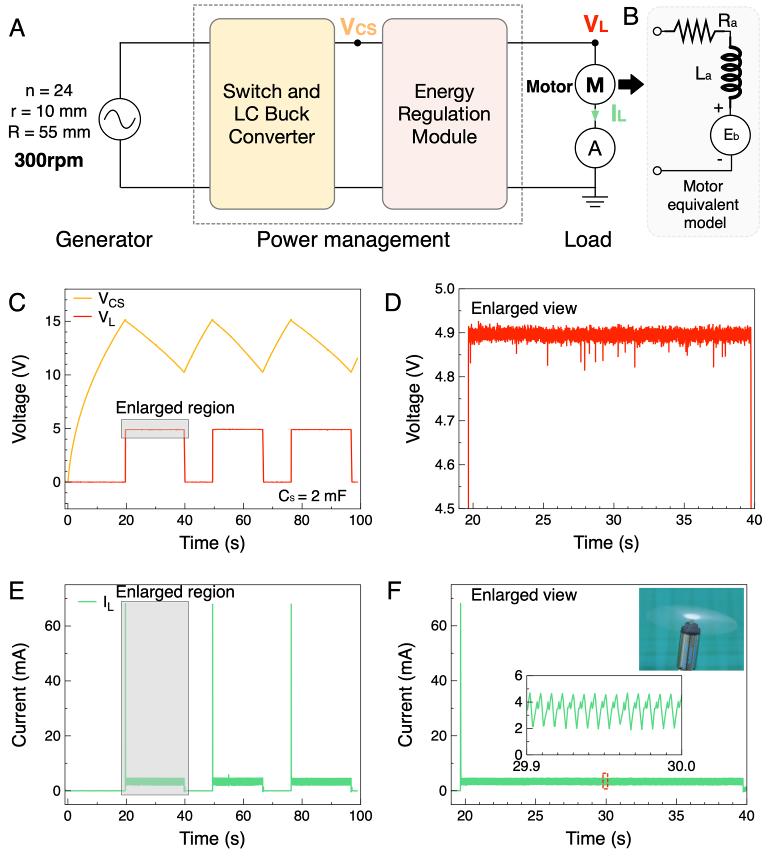

Figure R7 (S58). Demonstration of the power management system driving an inductive load (DC motor). (A) Schematic of the experimental setup. (B) Simplified equivalent circuit model of the DC motor. (C) Voltage after the LC buck converter (V_{CS}) and regulated DC output on the load (V_L). (D) Enlarged view of V_L. (E) Current through the load (I_L). (F) Enlarged view of I_L, with inset showing a further zoomed-in waveform.

Here, a DC motor is employed as the load to evaluate the reliability of the proposed power management system under inductive conditions (Figure S58A). Its simplified equivalent circuit model is shown in Figure S58B, consisting of the armature resistance R_a, the armature inductance L_a, and the back electromotive force (EMF) E_b, which is proportional to the angular velocity. The motor used here is the 08GS61-105C.3 from Portescap, with parameters R_a = 45.8 Ω, L_a = 271.6 μH, and a back-EMF constant of 0.53 V/1000 rpm. The testing results is shown in Figures S58C–F, in which the backup battery is disabled.

The system operates in intermittent mode (Figure S58C) because the motor's rated power exceeds the average power delivered by the generator after power management. At the beginning of each activation period, the motor starts from rest. Consequently, the current through the motor exhibits a transient surge (~68 mA, Figure S58E), as the EMF is negligible at this stage. As the rotor accelerates, the increasing back-EMF reduces the net voltage across the winding, and the current decays to a steady value (~3.5 mA, Figure S58F). In steady state, a small periodic ripple appears, mainly due to commutation and rotor-position-dependent variations in inductance and induced voltage. Nevertheless, throughout the entire cycle, the regulated output remains essentially constant (Figure S58D), demonstrating the robustness of the proposed power-management module.

5. In terms of reference citations for power management strategies, it's recommended to expand beyond the current references. Including studies on novel topological structures and intelligent control algorithms would enrich the research background and comparative analysis.

Answer: Thanks for your comments. In the revised manuscript, we have added related works on power management integrated with novel topological structures and intelligent control algorithms to enrich the research background and strengthen the comparative analysis.

Revision:

Line 69: Some multi-input single-inductor multi-output (MISIMO) systems[46-48] with novel topologies and intelligent control algorithms demonstrate excellent energy regulation performance. However, they are typically designed for low-voltage sources such as thermoelectric, photovoltaic, and piezoelectric energy harvesters, usually delivering only a few volts, and are not suitable for high-voltage electrostatic or triboelectric generators.

Reviewer #4 (Remarks to the Author):
Considering the importance of mechanical energy harvesters to power autonomous sensors, the authors propose a novel technique/strategy for the energy extraction. After reading the manuscript, the following doubts and questions have arisen:

Answer: We thank you for your valuable comments and positive feedback on our work.

1. Conceptually, the idea behind the proposed technique/strategy is not clearly explained. In other words, using as a reference the typical voltage-charge plot of a TENG, how is the cycle of energy output desired in the proposed circuit?

Answer: We thank you for your comments and sorry for not stating this clearly. We employed two peak switches and develop a bipolar peak switch topology, as shown in Fig. R8B. The switch closes at each peak of the generator's output (Fig. R8C, red arrows), meaning that it closes twice per voltage cycle (Fig. 8E). This makes the generator operate in the Cycle for Maximized Energy Output (CMEO) mode, thereby increasing the area enclosed by the V–Q plot and enhancing the output power (Fig. R8F). To clarify this, we have added the corresponding description in the main text and have added Fig. R8 as Fig. S1 in the revised Supplementary Information.

Revision:
Line 108: The switch closes at exactly the generator's output peak, making the generator operate in cycle for maximized energy output (CMEO) and converting the generator's continuous AC output to discrete high-energy pulses, thereby improving its output power (Figure S1) while enabling wide load compatibility.

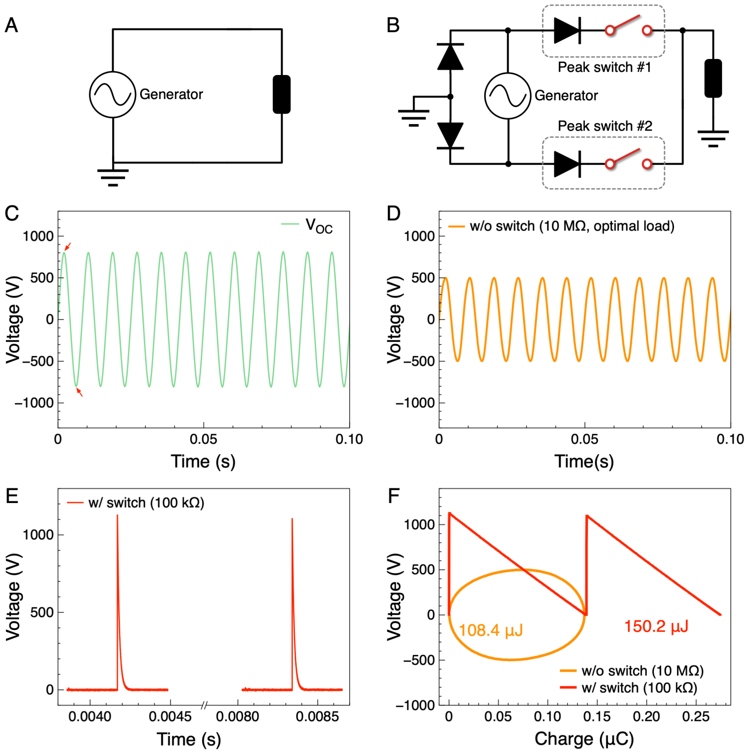

Figure R8 (S1). Demonstration of the energy-enhancement effect enabled by the synchronous switch. (A) Schematic of the generator with a resistive load (direct AC output). (B) Schematic of the generator with the bipolar peak-detection switch topology. (C) Open-circuit voltage of the generator, with red arrows indicating the closure times of the peak switch. (D) Direct AC output of the generator under the optimal load resistance. (E) Output of the generator with the synchronous switches. (F) V–Q plots and the corresponding output energy per cycle, with and without the switch.

2. Also, conceptually, the idea reflected in Fig. 1A needs more explanation. How the power at the second

stage (17.9 mW) can be higher than that in the first stage (13 mW)? Probably, the methodology to make the measurements explains this fact.

Answer: Thanks for your comments. As explained in response of comment #1, switching at the voltage peaks makes the generator operate in CMEO mode and increases the area enclosed by the V–Q plot, thereby enhancing the overall output power. As a result, the output power after the switch (17.9 mW, Stage 2) can be higher than the maximum direct AC output power without the switch (13.04 mW, Stage 1).

3. According to the circuit proposed in Fig. 2A, this is able to detect the positive peak, but do you also need to detect the negative peak? How many activations of the switch are required/desired per period? Of course, this is completely related with point #1. Depending on the desired V-Q plot, the number of activations will 1 or 2, but this is unclear in the current version of the manuscript.

Answer: Thank you for your comments and it's a good question. A single switch can detect only one voltage peak (either positive or negative). Therefore, for a bipolar voltage output, two switches are required, each triggered once per cycle, as explained in response of comment #1. The bipolar switch design was also shown in Fig. S34.

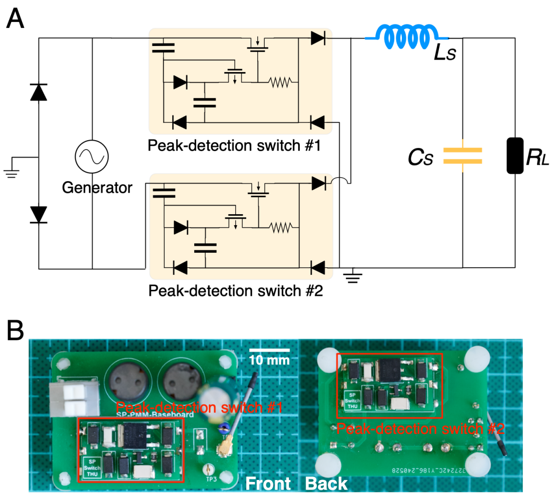

Figure R9 (S34). (A) Schematic and (B) photographs of the switches and LC buck converter used in the tests with rotational electret generators with bipolar output.

Revision:

Line 338: Here, to accommodate the generator's bipolar output, a switch topology with two peak-detection switches along with rectifying diodes is developed. The schematic of this switch topology, combined with an LC buck converter, is shown in Figure S34.

4. One of the critical issues (probably the most critical) related to triboelectric nanogenerators is the time-dependent inherent capacitance. Such effects are not clearly evaluated in the manuscript. For example: a) what is the relation required between the inherent TENG capacitance and the capacitances of the peak-detection switch (Fig. 2A) in order to guarantee an appropriate operating principle?

Answer: Thanks for your comment and it's a good question. In fact, there is no required relationship between the generator's inherent capacitance and the switch capacitance for ensuring switch triggering, because the closure of the switch depends solely on the voltage change after the generator output reaches its peak, and is unrelated to the generator's capacitance. This is because the startup trigger originates from the conduction of the P-MOSFET, which is determined only by its gate–source voltage difference,

itself set solely by the generator's post-peak voltage change. We have added this clarification in the revised manuscript to make our explanation clearer.

That said, it is still recommended that the generator capacitance be as large as possible compared to the capacitance of the switch, so as to reduce the loading effect and ensure efficiency.

Revision:

Line 164: Notably, the trigger of the positive feedback process is governed solely by the voltage drop occurring after the peak, allowing the switch to operate stably even with varying generator capacitance, as long as the voltage drop meets the trigger condition.

b) How the effects of the time-dependent TENG capacitance are considered in test platform shown in Fig. 2B? It is true that the effects of different generator capacitances are shown in Figs. 2E, F, and G, but in TENGs the capacitance changes dynamically with time, and this is not reflected in the current version of manuscript.

Answer: Thanks for your comments. In the current work, we considered only variations in generator capacitance without time dependence, since the capacitance of rotating electret/dielectric-freestanding triboelectric generators discussed here depends primarily on the electrode pairs and remains essentially constant during operation. As discussed in Comment #4a, capacitance variation does not affect the switch's triggering; therefore, the switch can also operate with generators (e.g., CS-mode TENG) that have time-dependent capacitance.

Nevertheless, evaluating the switch's performance under such conditions is important and is planned as part of our future work.

References

1. Cheng, X. *et al.* High efficiency power management and charge boosting strategy for a triboelectric nanogenerator. *Nano Energy* **38**, 438–446 (2017).
2. Song, Y. *et al.* High-efficiency self-charging smart bracelet for portable electronics. *Nano Energy* **55**, 29–36 (2019).
3. Liu, Y., Shi, Z., Badel, A., Miyoshi, T. & Suzuki, Y. Self-Powered Synchronous Electric Charge Extraction Rectifier for Rotational Electret Energy Harvester With Dual-Stage Electrodes. *IEEE Trans. Power Electron.* **38**, 13166–13180 (2023).
4. Liufu, Y. H., Dai, D. & Liu, Z. Triple-MOSFETs switch for adaptive maximum capacitance point tracking of triboelectric nanogenerators. *Nano Energy* **106**, 108042 (2023).
5. Zhen, D. *et al.* A Triboelectric Energy Harvesting IC With High-Voltage Synchronous Electric Charge Extraction Strategy and Superior Systematic Efficiency. *IEEE Trans. Circuits Syst. Regul. Pap.* 1–13 (2024) doi:10.1109/TCSI.2024.3405900.
6. Park, I. *et al.* A 4.5-to-16μW integrated triboelectric energy-harvesting system based on high-voltage dual-input buck converter with MPPT and 70V maximum input voltage. in *2018 IEEE International Solid - State Circuits Conference - (ISSCC)* 146–148 (IEEE, San Francisco, CA, 2018). doi:10.1109/isscc.2018.8310226.
7. Park, I., Maeng, J., Shim, M., Jeong, J. & Kim, C. A Bidirectional High-Voltage Dual-Input Buck Converter for Triboelectric Energy-Harvesting Interface Achieving 70.72% End-to-End Efficiency. in *2019 Symposium on VLSI Circuits* C326–C327 (IEEE, Kyoto, Japan, 2019). doi:10.23919/vlsic.2019.8778018.
8. Wang, Z. *et al.* Ultrahigh Electricity Generation from Low-Frequency Mechanical Energy by Efficient Energy Management. *Joule* **5**, 441–455 (2021).
9. Dai, Y. *et al.* Effective Charging of Commercial Lithium Cell by Triboelectric Nanogenerator with Ultrahigh Voltage Energy Management. *Adv. Sci.* **11**, 2404253 (2024).
10. Wu, H. *et al.* Efficient energy conversion mechanism and energy storage strategy for triboelectric nanogenerators. *Nat. Commun.* **15**, 6558 (2024).
11. Wang, Z. *et al.* A universal self-triggered passive management strategy for enhancing the output power of triboelectric nanogenerators. *Energy Environ. Sci.* **18**, 3761–3772 (2025).
12. Luo, Y. *et al.* Advanced Energy Harvesting from Low-Frequency Ocean Waves for Lithium-Ion Battery Applications. *Energy Environ. Sci.* 10.1039.D5EE01492A (2025) doi:10.1039/D5EE01492A.
13. Tu, Y. *et al.* IC-Compatible High-Efficiency Power Management for Triboelectric Nanogenerators Based on the Concept of Limit. *IEEE Trans. Power Electron.* **39**, 6–13 (2024).
14. Lee, J. *et al.* A Triboelectric Energy-Harvesting Interface With Scalable Multi-Chip-Stacked Bias-Flip and Daisy-Chained Synchronous Signaling Techniques. *IEEE J. Solid-State Circuits* **57**, 3825–3839 (2022).
15. Lee, S.-H., Jeong, Y.-W., Park, S.-J. & Shin, S.-U. A Rectifier-Reusing Bias-Flip Energy Harvesting Interface Circuit With Adaptively Reconfigurable SC Converter for Wind-Driven Triboelectric Nanogenerator. *IEEE Trans. Ind. Electron.* **70**, 8022–8031 (2023).
16. Liu, Y., Badel, A. & Suzuki, Y. Enhancing output power of rotational electret energy harvester by synchronized switch harvesting on inductor. *J. Intell. Mater. Syst. Struct.* **33**, 183–195 (2022).
17. Ghaffarinejad, A. *et al.* Bennet's doubler working as a power booster for triboelectric nano-generators. *Electron. Lett.* **54**, 378–379 (2018).
18. Zhang, H., Lu, Y., Ghaffarinejad, A. & Basset, P. Progressive contact-separate triboelectric nanogenerator based on conductive polyurethane foam regulated with a Bennet doubler conditioning circuit. *Nano Energy* **51**, 10–18 (2018).
19. Hosseini, A., Badeli, A. S., Davari, M., Sheikhaei, S. & Gharehpetian, G. B. A Novel, Software-Defined Control Method Using Sparsely Activated Microcontroller for Low-Power, Multiple-Input, Single-Inductor, Multiple-Output DC–DC Converters to Increase Efficiency. *IEEE Trans. Ind. Electron.* **70**, 2959–2970 (2023).

20. Li, S., Liu, X. & Calhoun, B. H. A 32nA Fully Autonomous Multi-Input Single-Inductor Multi-Output Energy-Harvesting and Power-Management Platform with 1.2×10^5 Dynamic Range, Integrated MPPT, and Multi-Modal Cold Start-Up. in *2022 IEEE International Solid- State Circuits Conference (ISSCC)* 1–3 (IEEE, San Francisco, CA, USA, 2022). doi:10.1109/ISSCC42614.2022.9731732.
21. Wang, J. *et al.* A Cross Regulation Reduced Multi-Output and Multi-VCR Piezoelectric Energy Harvesting System Using Shared Capacitors. in *2022 IEEE International Symposium on Circuits and Systems (ISCAS)* 2768–2772 (IEEE, Austin, TX, USA, 2022). doi:10.1109/ISCAS48785.2022.9937938.
22. Duc Truong, B. & Phu Le, C. Theoretical analysis of electrostatic energy harvester configured as Bennet's doubler based on Q-V cycles. *Int. J. Circuit Theory Appl.* **51**, 2518–2543 (2023).

Point-to-Point Response to the Reviewer's Comments
(Comments in black, response in blue, revisions are highlighted)

We sincerely thank you for your continued support of our work and for your detailed, insightful comments and suggestions. We have revised the manuscript accordingly, and the detailed corrections are listed below point by point.

Reviewer #1 (Remarks to the Author):

a) we ask you to further clarify how the switch's operating voltage is adjusted in Fig. R3, including the brief info about it in the main manuscript. We also ask you to clarify which IC technology can be used for the anticipated voltage level. b) In confidence, the reviewers also questioned whether it's appropriate to refer to CMEO in Table S2 as an energy extraction strategy itself.

Answer: Thanks for your comments.

a) The maximum operating voltage of the switch is determined by the drain–source voltage tolerance of the N-MOSFET; exceeding this limit can lead to drain–source breakdown and permanent damage to the device. The minimum operating voltage is determined by the minimum charge required by C_{Drive} ($Q_{req,min}$) to properly activate the switch. To ensure correct switching, the driving charge Q_{Drive} = V_{EG} × C_{ser} must exceed $Q_{req,min}$, where C_{ser} is the series equivalent capacitance of C_{in} and C_{Drive}. Increasing C_{in} or removing the capacitive divider can increase Q_{Drive}, thereby reducing the minimum operating voltage ($V_{EG,min}$) of the switch.

As for the IC technology, in this work, we used a discrete N-channel MOSFET (IXTY02N120P, listed in Table S4) from the Littelfuse Polar™ Standard Power MOSFET series, which is a silicon (Si) MOSFET fabricated using a proprietary planar MOSFET process. The IXTY02N120P can handle the generator's output voltage within 1200 V. For applications involving higher generator voltages, silicon carbide (SiC) or other high-voltage MOSFETs with higher drain-source voltage tolerance would be required.

These pieces of information have been added at line 343 of the main text.

Revision:

Line 343: Note that this structure can also accommodate generators with asymmetric outputs (e.g., contact–separation mode TENGs), provided that each switch's operating voltage range corresponds to the generator voltage of its respective polarity. The switch's operating voltage range can be adjusted by using a MOSFET with a higher drain–source voltage tolerance (e.g., a SiC MOSFET) to increase the upper limit, and by increasing the switch's input capacitance (C_{in}) to lower the minimum limit.

b) We appreciate this insightful comment regarding whether CMEO can be regarded as an energy extraction strategy itself. We consider that it can indeed be viewed as such, since the synchronous switching mechanism in CMEO allows the generator to deliver a constant and maximal energy output with low output impedance—an operation that can be regarded as an energy extraction process. In contrast, the subsequent circuits (e.g., an LC buck converter or the load) merely convert or consume this extracted energy. Therefore, we consider it reasonable to classify CMEO as an energy extraction strategy in Table S2.

Reviewer #4 (Remarks to the Author):

1. Several doubts arise from Fig. R8. For example: a) Considering a resistive load is not the common case considered in the literature. Why do you assume that? b) Why there are no negative voltages in Fig. R8F for the performance in red? Note that, in many cases, authors talk about a bipolar output of the generator, but according to that figure, the output is unipolar. In addition, this figure should be in the main text, not in the supplementary file. c) Again for Fig. R8F, the performance without the switch (in orange) is unclear.

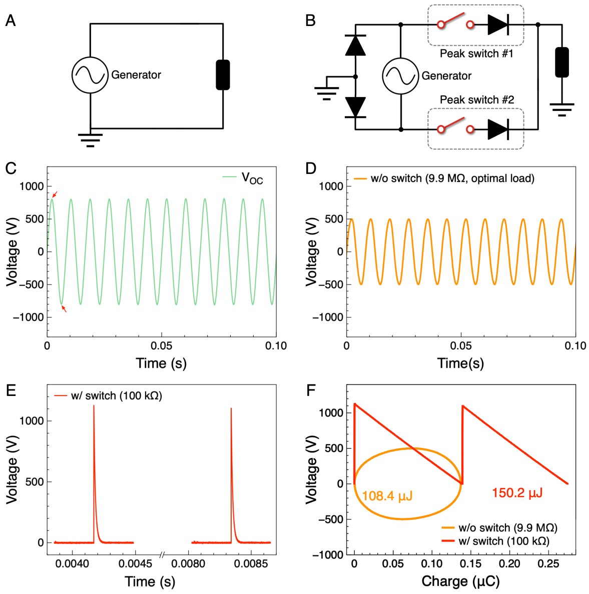

Figure R1 (S1). Demonstration of the energy-enhancement effect enabled by the synchronous switch. (A) Schematic of the generator with a resistive load (direct AC output). (B) Schematic of the generator with the bipolar peak-detection switch topology. (C) Open-circuit voltage of the generator, with red arrows indicating the closure times of the peak switch. (D) Direct AC output of the generator under the optimal load resistance. (E) Output of the generator with the synchronous switches. (F) V–Q plots and the corresponding output energy per cycle, with and without the switch.

Answer: Thank you for your comments. For the convenience, Fig. R8 from the previous revision is re-displayed here as Fig. R1.

a) In fact, using a resistive load to evaluate power/energy is an easy, common, and widely used way in the literature for evaluating the output performance of triboelectric and electrostatic generators, as well as for power management circuits.[1–5] Notably, the original publication introducing the CMEO itself employed resistive loads to calculate and experimentally verify the CMEO value.[6] Thus, it is justified to use a resistive load to demonstrate the output performance of the generator with and without the switches.

b) The output of the generator is inherently bipolar, but it appears unipolar at the load because a full-wave bridge rectifier is included in the circuit, as shown in Fig. R1B. This rectifier structure has also been added in Fig. 1A to clarify how the generator's output changes from AC (without switches) to unipolar (with switches).

Regarding the V–Q plot in Fig. R1(S1)F, it represents the maximum output energy of the generator with and without switches during an operation cycle. This energy value is derived from the same data for the average power values shown in Fig. 1A (13.04 mW without the switch and 17.9 mW with the switch). Because the energy representation conveys essentially the same information as the average power, but in a less intuitive form for most readers, thereby we believe it is more appropriate to be included in the Supplementary Information. This allows interested readers to access the detailed energy analysis without overcomplicating the main text.

Revision:

Displayed in the dashed box above *Energy Boosting* in Fig. 1A (R2).

c) The area of the orange V–Q plot in Fig. R1F represents the maximum energy that the generator can deliver to the load in one cycle without using switches. It serves as a figure of merit for evaluating the intrinsic maximum output capability of triboelectric and electrostatic generators. The orange V-Q plot is derived from the generator's output voltage under the optimal load condition shown in Fig. R1D, which is also used to calculate the maximum average power on the optimal load presented in Fig. 1C.

2. The reviewer understands the explanation provided by the authors, but in that case, the terminology and drawings employed in the manuscript should be modified. Talking about stages 1, 2, … seems that the circuit has different blocks in cascade, and then the numbers of power provided make no sense.

Answer: Thank you for your comment and helpful suggestion.

Our intention in listing the power values in Fig. 1A was to illustrate how the overall output power changes when additional modules are introduced, rather than to represent the power of individual stages connected in cascade. For example, we aimed to clearly show that adding the peak switches increases the output power from 13.04 mW to 17.9 mW. This way, readers can clearly see how the addition of each module affects the output.

We agree that the presentation of Fig. 1A in the previous version did not clearly convey our intention and may have caused misunderstanding. We have therefore revised the Fig. 1A to separately show a generator without a switch (13.04 mW) and one with a synchronous switch (17.9 mW), in order to avoid any misinterpretation suggesting a violation of energy conservation.

The output power of 17.9 mW after switch is then converted into a low-voltage output by the buck converter, whose output voltage varies between 11–16 V due to the UVLO constraint (Fig. S2C), delivering 15.2 mW across a wide load range (Fig. S2H). Then, this output is further converted by the energy regulation module, resulting in a final output power of 14.2 mW. The power values in red font in Fig. 1A are the output power obtained when the module is added, which illustrate how the power changes with the modules.

We consider these red power values to be reasonable even for the entire cascade, although they may not be 100% precise. This is because the inclusion of the LC buck converter does not affect the intrinsic output power of the generator with the switches, and the converter's output power is solely determined by its output voltage (Fig. 3). Since this voltage is constrained by the UVLO, the power delivered to the subsequent energy regulation module is also determined and therefore remains approximately 15.2 mW.

However, to clarify the meaning of the power values and eliminate any potential ambiguity, we have also carefully revised the corresponding text for Fig. 1A, as highlighted in the revision

Revision:

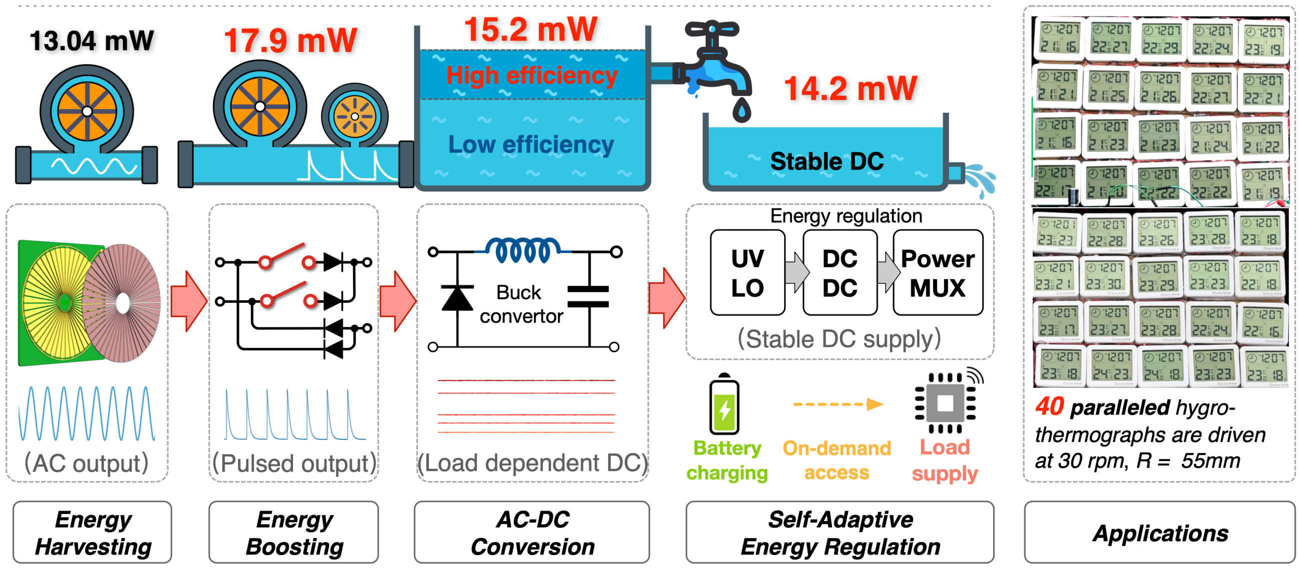

Figure R2. Overview of the Power Management System for Continuous and Stable Power Delivery

Line 106: Effective power management of triboelectric and electrostatic generators relies on three key processes: efficient energy extraction, conversion, and utilization. As shown in Figure 1A, we first propose a self-powered peak-detection switch to enable efficient energy extraction. The switch closes at exactly the generator's output peak, making the generator operate in cycle for maximized energy output (CMEO) and converting the generator's continuous AC output to discrete high-energy pulses (Figure S1), thereby improving its output power from 13.04 mW to 17.9 mW while enabling wide load compatibility. After that, a passive LC buck converter is employed for AC-DC conversion. However, its output is load-dependent, and the conversion efficiency drops sharply at low output voltages when powering conventional electronics. To mitigate this, we analyzed the energy losses in buck circuits and introduced a voltage elevation strategy to enhance AC-DC conversion efficiency. This strategy is subsequently integrated into the energy regulation module by implementing an under-voltage lockout (UVLO) module, achieving a 15.2 mW output from the LC buck converter. Additionally, to enable efficient energy utilization, the energy regulation module further incorporates a step-down DC-DC converter for stable voltage output and a self-adaptive power multiplexer (MUX) that dynamically allocates the harvested energy between the load and the backup battery according to working conditions. Together, these components form a power management system that enables efficient AC-to-DC conversion for triboelectric and electrostatic generators, providing a continuous and stable output of 14.2 mW for driving practical applications. Detailed experimental data collected during the addition of each power management module are shown in Figure S2.

3. Now it is clear that there are two activations per cycle and the goal is to achieve the CMEO. In that case, before the peak, the TENG has to be in open circuit but, according to Fig. 2 A(i) there is a current path (highlighted in red). How do you explain that?

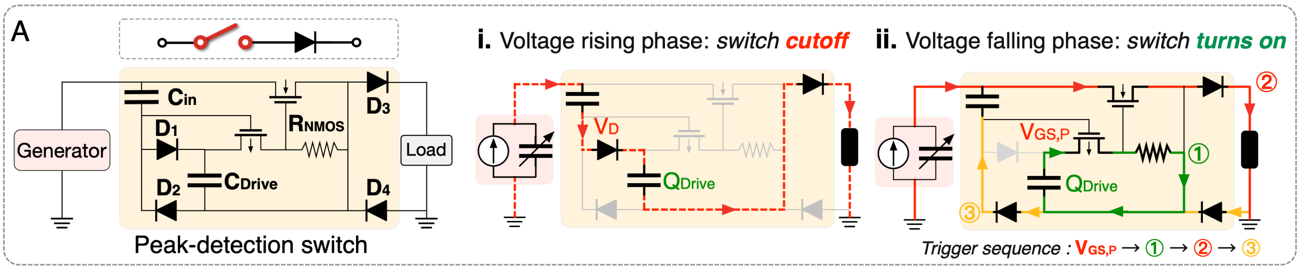

Figure R3. Schematic and working principle of the self-powered peak-detection switch.

Answer: Thank you for your comment. For the convenience, Fig. 2A is re-displayed here as Fig. R3.

We approve that there is indeed a current flow along the CEG → Cin → D1 → CDrive → D3 → load path in Fig. 2A(i), which serves to charge C_{Drive} and subsequently drives the switch. However, it is important to note that this path differs fundamentally from the one established after the switch closes (the red solid line ② in Fig. 2A(ii)). In Fig. 2A(ii), once the switch closes, the charge flows directly through the switch (upper NMOS) and is ultimately neutralized to ground. In contrast, in the current path shown in Fig. 2A(i), no charge from the generator actually flows through C_{in} and C_{Drive}; rather, the observed current arises from charge redistribution along the path. The current path in Fig. 2A(i) therefore does not consume the charge generated by the generator.

For CMEO operation, it is indeed required that the generator remains under open-circuit conditions before its output voltage reaches the peak. However, in this context, the term "open circuit" more likely refers to a condition where no net charge generated by the generator is transferred or eventually neutralized to ground. For example, due to inevitable parasitic capacitance, such as those introduced by wiring and surrounding circuits, there is always a small capacitive current path in parallel with the generator, as show in Fig. R4A. When the generator voltage varies, a "current", namely displacement current, will flow through this parasitic capacitance to the ground. Nevertheless, this process does not reduce the total charge produced by the generator, and the generator is still generally regarded as operating under open-circuit conditions.

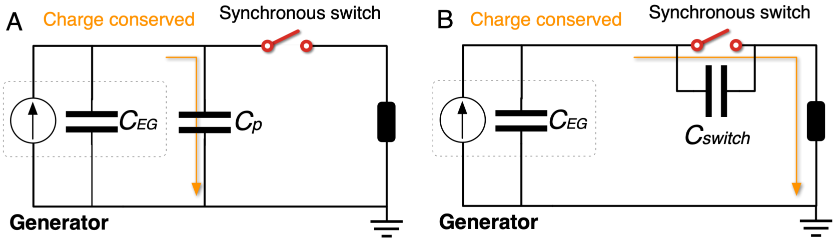

Figure R4. (A) Illustration of parasitic capacitance before the switch. (B) Illustration of the parasitic capacitance associated with the switch.

It is the same for the capacitance of the switch (C_{switch}), which can originate from parasitic capacitance of the switch or from the capacitor introduced by our switch topology, as shown in Fig. R2B. The charge generated by the generator is still not reduced during the switch open. This capacitance, C_{switch}, is equivalent to C_p as seen by the generator, as both are connected in parallel with C_{EG}. C_{switch} and C_p together serve as the loading capacitance of the generator.

However, although no charge consumption occurs through the parasitic capacitance C_p and the capacitors C_{in} and C_{Drive} in our switch, the associated charge redistribution can still cause a voltage drop and hence an energy loss in the generator system—a phenomenon known as the loading effect. According to charge conservation, the output voltage under a total loading capacitance C_L is given by

$$V_{\text{out},CL} = V_{\text{out}} \frac{C_{\text{L}}}{C_{\text{EG}} + C_{\text{L}}}$$

where C_{EG} is the generator capacitance and C_L represents the total loading capacitance, including the parasitic capacitance (C_p) and the switch capacitance (C_{switch}). That's why we recommend a higher generator capacitance.

4. a) The answer provided is not convincing at all. First, it seems that no relationship is required, but then "it is still recommended that the generator capacitance be as large as possible compared to the

capacitance of the switch, so as to reduce the loading effect and ensure efficiency". It is completely unclear. How much is the capacitance of the TENG? How much are the capacitances of the peak detector? How much is the reduction of the TENG voltage after introducing the peak detector?

b) The sentence "the switch can also operate with generators (e.g., CS-mode TENG) that have time-dependent capacitance" needs some extra evaluation depending on the answers provided in the previous point.

Answer: Thank you for your comment.

a) We would like to clarify that the switch triggering is governed by two conditions: (1) Charge accumulation, where C_{Drive} must accumulate sufficient charge to drive the switch during the voltage-rising phase; and (2) Trigger slope, where the post-peak voltage decay of the generator must be steep enough to initiate the positive-feedback mechanism responsible for switch activation.

For the first condition, the charge accumulated on C_{Drive} can be expressed as

$$Q_{\text{Drive}} = V_{\text{out,max}} \frac{C_{\text{in}} \times C_{\text{Drive}}}{C_{\text{in}} + C_{\text{Drive}}}$$

where $V_{out,max}$ is the maximum generator output voltage. It is therefore clear that the accumulated charge depends on the generator's output voltage, and C_{in} and C_{Drive} as in Fig. 2A, not generator's capacitance.

For the second condition, the switch is triggered by establishing $V_{GS,P}$, which drives the P-MOSFET into conduction and initiates the positive-feedback process. The formation of $V_{GS,P}$ depends solely on the post-peak voltage change ΔV_{out}, and again not on the generator's capacitance.

Taken together, these two conditions indicate that switch activation is determined by the generator's voltage and its variation, rather than by its capacitance, demonstrating that our circuit is compatible with generators exhibiting either constant or variable capacitance.

However, as stated in the response to comment #3, introducing the switch increases the total loading capacitance seen by the generator, leading to a decrease in the output voltage and energy of the generator system. Therefore, we recommend a design with a higher generator capacitance. In our setup, C_{switch} is approximately 16 pF, corresponding to the series combination of Cin and CDrive, together with the parasitic drain–source capacitance ($C_{DS,NMOS}$) of the N-MOSFET (Table S3). The generator capacitance (C_{EG}) varies depending on its physical configuration, as summarized in Table S5. By setting the parasitic capacitance to zero and applying the equation given in the response to comment #3, the voltage reduction caused by the switch can be quantitatively estimated.

b) In addition to the theoretical analysis above, we further conducted SPICE simulations using LTspice with a contact–separation-mode generator whose capacitance varies dynamically between 169.9 pF and 16.0 pF over time, to provide direct supporting evidence. The detailed parameter of the generator is shown below.

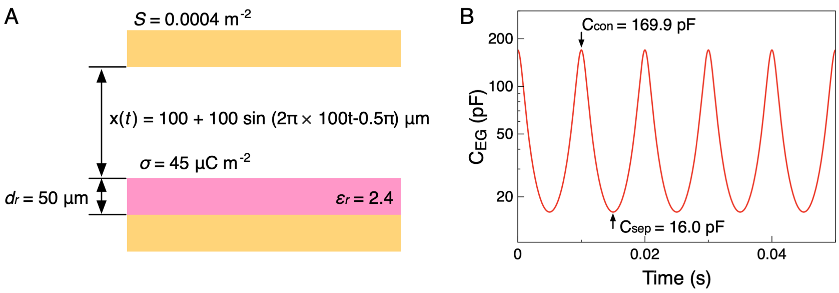

Figure R5. (A) Parameters of the contact–separation-mode generator. (B) Capacitance variation of the generator over time.

By incorporating both the generator model and our switch design into the simulation, we obtained the generator's output after the switch, as shown in Fig. R6. The results show that the switch closes appropriately at the correct moments of contact and separation, producing a rapid voltage rise across the load followed by a typical RC-type discharge curve. At separation point, where the generator's voltage is high and the capacitance is small, the voltage decays by 95% within 5.4 μs—consistent with three-time constants of $3R_LC_{sep}$. At the contact point, where the voltage is lower and the capacitance is larger, the 95% decay time is 53.8 μs—consistent with three-time constants of $3R_LC_{con}$.

The simulation results provide strong evidence that our switch operates appropriately with generators featuring time-dependent capacitance.

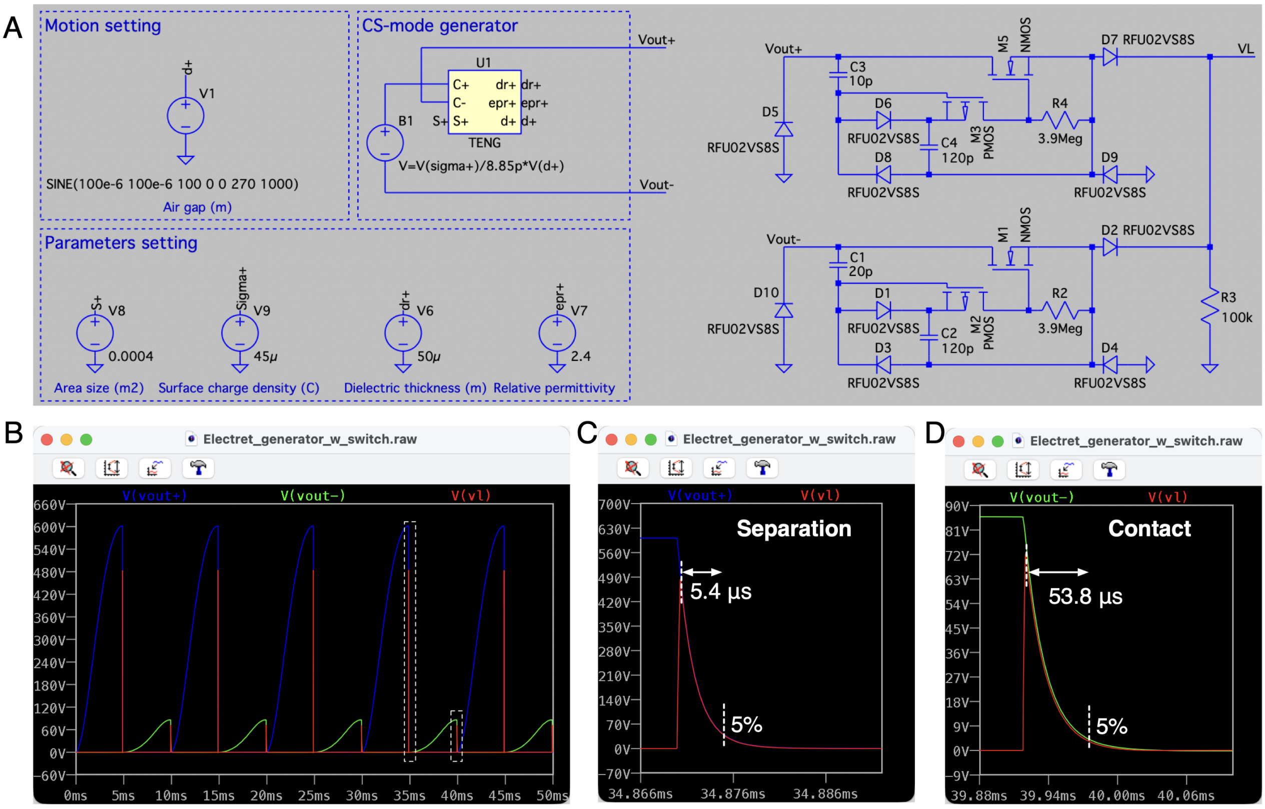

Figure R6. Simulation results of the capacitance-variation generator with peak-detection switches. (A) Schematic of the simulation. (B) Node voltages of the generator and load, with their enlarged views corresponding to the generator's separation and contact states shown in (C) and (D).

References

1. Wang, Z. *et al*. A universal self-triggered passive management strategy for enhancing the output power of triboelectric nanogenerators. *Energy Environ. Sci.* **18**, 3761–3772 (2025).

2. Niu, S. & Wang, Z. L. Theoretical systems of triboelectric nanogenerators. *Nano Energy* **14**, 161–192 (2015).

3. Sabzpoushan, S. & Woias, P. Electret-based energy harvesters: A review. *Nano Energy* **131**, 110167 (2024).

4. Wang, Z. *et al*. Ultrahigh Electricity Generation from Low-Frequency Mechanical Energy by Efficient Energy Management. *Joule* **5**, 441–455 (2021).

5. Wu, H. *et al*. Efficient energy conversion mechanism and energy storage strategy for triboelectric nanogenerators. *Nat. Commun.* **15**, 6558 (2024).

6. Zi, Y. *et al*. Standards and figure-of-merits for quantifying the performance of triboelectric nanogenerators. *Nat. Commun.* **6**, 8376 (2015).

Point-to-Point Response to the Reviewer's Comments
(Comments in black, response in blue, revisions are highlighted)

Reviewer #4 (Remarks to the Author):

1. Figure R1 from the response to reviewer file. Case (E): is this the output of the generator or the signal applied to the load? Why this voltage is higher than the open-circuit voltage represented in Fig. C? Case (F): even with the inclusion of the full-wave rectifier, the V-Q plot generally shows the performance of the TENG for the negative half cycle, but this is not reflected in your figure. And again, why this voltage is higher than the open-circuit voltage in Fig. C?

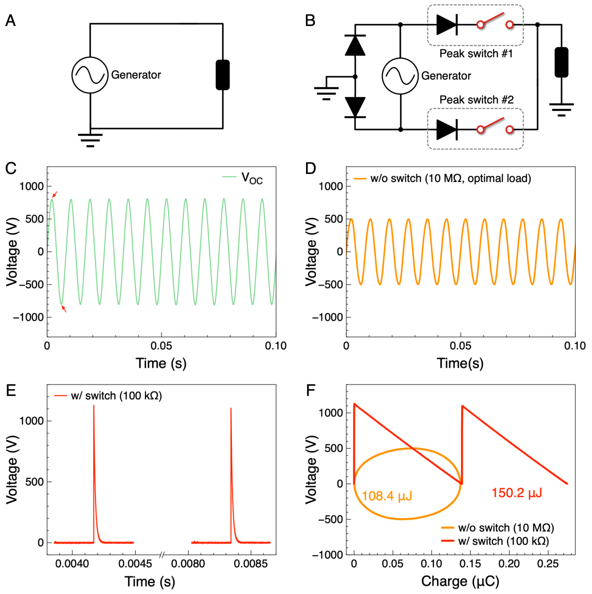

Figure R1 (S1). Demonstration of the energy-enhancement effect enabled by the synchronous switch. (A) Schematic of the generator with a resistive load (direct AC output). (B) Schematic of the generator with the bipolar peak-detection switch topology. (C) Open-circuit voltage of the generator, with red arrows indicating the closure times of the peak switch. (D) Direct AC output of the generator under the optimal load resistance. (E) Output of the generator with the synchronous switches. (F) V–Q plots and the corresponding output energy per cycle, with and without the switch.

Answer: Thank you for your comments.

a) Yes, Fig. R1E shows the voltage of the generator with the switch on a 100 kΩ load, and its peak voltage is indeed higher than the open-circuit voltage shown in Fig. R1C. This is because, for a rotational generator, the open-circuit voltage (V_{oc}) is defined as the peak output voltage, which corresponds to half of the peak-to-peak voltage of the generator (2 V_{oc}). When a synchronous switch is introduced, it enables additional charge to accumulate on the generator's capacitor beyond the conventional open-circuit condition, allowing its output voltage to reach up to the theoretical peak-to-peak limit of 2 V_{oc}.[1,2] In practice, non-ideal factors such as the parasitic capacitance of the load limit the output voltage with the switch, so the actual output is generally lower than 2 V_{oc}. In our case, the peak reaches approximately 1.4 V_{oc}.

b) The V–Q plot represents the output energy per cycle, obtained from the voltage across and the charge transferred through the external load. Since both the output voltage across the load and the charge-transfer direction are rectified to be positive, the resulting V–Q plot for one cycle consists of two continuous, unclosed positive triangular traces. This representation is adopted to maintain consistency and avoid misinterpretation, and it is commonly used for generators with rectified single-polarity outputs.[3–5]

2. There is no significant improvement in Figure 1 to better show what was indicated in the comment of the previous round.

Answer: Thank you for your comment. The comment in the previous round focused on the concern that the power values might not make sense in a cascade structure. However, as we responded earlier, our intention is to illustrate how the overall output power changes when additional modules are introduced, rather than to represent the power of individual stages connected in cascade. To avoid such misunderstanding:

1. We separated the illustration of (i) the generator without the switch and (ii) the generator with the switch in the last revision. This revision removes any potential misunderstanding regarding a perceived violation of energy conservation, given the apparent power increase from 13.04 mW to 17.9 mW.

2. Regarding the power values from 17.9 mW to 14.2 mW, we kept them because they clearly show how the addition of each module affects the output power. To avoid any misunderstanding, we also revised the text below Fig. 1 to explicitly indicate, for every added module in the cascade, the corresponding power value obtained after it is included. This allows readers to clearly understand that these values are obtained when each module is added, without any misunderstanding.

We believe our revision has conveyed the intended meaning unambiguously and resolved the previous source of misunderstanding, with the terminology and drawings presented correctly.

3. According to the equation provided in the response-to-reviewers file, it seems that the higher the value of CL, the better, since then V_out,CL is equal to V_out and we have the open-circuit conditions. But this makes no sense, CL should be as low as possible. Therefore, the derivation of this equation and/or the explanation around it are erroneous?

Answer: Thank you for your comment and we are sorry for the mistakes. The correct equation for loading effect is:

$$V_{\mathrm{out},CL} = V_{\mathrm{out}} \frac{C_{\mathrm{EG}}}{C_{\mathrm{EG}} + C_{\mathrm{L}}}$$

4. It seems that no new information has been included in the main manuscript regarding the effects and relation of the capacitances.

Answer: Thank you for your comment. In the first-round revision, we added an explanation in Line 166 stating that "Notably, the trigger of the positive feedback process is governed solely by the voltage drop occurring after the peak, allowing the switch to operate stably even with varying generator capacitance, as long as the voltage drop meets the trigger condition." To make this point more explicit, we have further revised the text.

Revision:

Line 166: Notably, the trigger of the switch is determined by sufficient charge accumulation before the voltage peak and the subsequent voltage drop and is essentially independent of the generator capacitance. Therefore, as long as these trigger conditions are met, the switch can operate properly even with varying generator capacitance.

References

1. Cao, Z., Wang, S., Bi, M., Wu, Z. & Ye, X. Largely enhancing the output power and charging efficiency of electret generators using position-based auto-switch and passive power management module. *Nano Energy* **66**, 104202 (2019).
2. Zhen, D. *et al.* A Triboelectric Energy Harvesting IC With High-Voltage Synchronous Electric Charge Extraction Strategy and Superior Systematic Efficiency. *IEEE Trans. Circuits Syst. Regul. Pap.* 1–13 (2024) doi:10.1109/TCSI.2024.3405900.
3. Wang, Z. *et al.* Ultrahigh Electricity Generation from Low-Frequency Mechanical Energy by Efficient Energy Management. *Joule* **5**, 441–455 (2021).
4. Xu, S. *et al.* Self-doubled-rectification of triboelectric nanogenerator. *Nano Energy* **66**, 104165 (2019).
5. Liu, W. *et al.* Switched-capacitor-convertors based on fractal design for output power management of triboelectric nanogenerator. *Nat. Commun.* **11**, 1883 (2020).

Point-to-Point Response to the Reviewer's Comments
(Comments in black, response in blue, revisions are highlighted)

Reviewer #5 (Remarks to the Author):

This manuscript reports an energy management strategy for triboelectric or electrostatic generators based on a self-powered peak-tracking switch, demonstrating excellent energy management performance for devices operating in the independent triboelectric layer mode. The manuscript has undergone multiple rounds of revision and has reached a high-quality level. The authors' responses to the reviewers' comments, particularly those addressing the concerns of Reviewer #4, are accurate and appropriate. Regarding the energy management strategy proposed in this manuscript, I have only one concern: what are the key innovations or differences between the self-powered peak-tracking switch strategy presented here and the one described in this paper (Nano Energy 131 (2024) 110271)?

Answer: We appreciate the reviewer for the positive evaluation of our manuscript and revisions.

Regarding the concern raised, although both works employ a self-powered peak-tracking concept, the present design is not a simple substitution of devices. Its key advance lies in a switching architecture tailored for high-output triboelectric/electrostatic generators. Specifically, compared with the previously reported BJT-based scheme, which was experimentally demonstrated up to 180 V, our design uses a high-voltage MOSFET together with a capacitor-divider driving network, enabling reliable operation from the hundred-volt range up to 1200 V. In addition, because the MOSFET-based design is voltage-driven and does not require sustained drive current in the on-state, it reduces the required input capacitance of the switch circuit from 220 pF in the mentioned work to below 10 pF in the present work, thereby imposing a smaller loading effect on the generator. This is beneficial for preserving the generator output voltage and improving total energy output. Moreover, the proposed switch features a positive-feedback-assisted turn-on process, achieving a measured transition time of less than 32 ns, which enables a rapid off-to-on transition and reduces loss during the switching transient. Therefore, the main distinction of this work is a high-voltage-compatible, low-loading effect, and fast-response self-powered peak switch well suited for high-output triboelectric and electrostatic generators.

We have revised the manuscript accordingly by adding a brief discussion of the previously reported self-powered transistor-based peak-detection switch in the Introduction, and by further clarifying its differences from the present design in Supplementary Table 2.

Revision:

1. Lines 64-67: Recently, a self-powered transistor-based peak-detection switch has been proposed to simplify the control process and enable autonomous operation.[40] However, it still suffers from a limited operating-voltage range, a non-negligible loading effect, and slow switching response.

Supplementary Table 2. Comparison of power management performance with previous works.

References	Energy Source	Energy Optimization Strategy	Switch Type	Switch Architecture	Self-Powered/Cold Start	Static Power Consumption	Tolerated input voltage	Operation Frequency	Convertor Topology	Energy Regulation	Performance metric #1 $P_{reg}^{**}/P_{AC,opt}^{**}$	Performance metric #2 P_{reg}/P_{CMEO}^{**}	Other performance metric
Nano Energy (2017)[5]	Triboelectric (In-plane)	CMEO*	Peak	Discrete controller + low-side N-MOSFET	No	2.69 μW	<450 V	N/A	Flyback	No	N/A	N/A	$P_{unreg}^{**}/P_{AC,opt}^{**}$ 74.45% @ 4.47 V
Nano Energy (2019)[6]	Triboelectric (In-plane)	CMEO	Peak	Discrete controller + low-side N-MOSFET	No	2.69 μW	<305 V	N/A	LC buck	No	N/A	N/A	$P_{unreg}/P_{AC,opt}$ = 69.3% @ 1.7 V
IEEE Transactions on Power Electronics (2023)[7]	Electret (In-plane)	CMEO	Peak	Discrete controller + low-side N-MOSFET	Yes (Delayed start)	1~2 μW	<250 V	~300 Hz	LC buck	No	N/A	N/A	$P_{unreg}/P_{FBR+Buck}^{**}$ = 2.1 @ 4.6 V
Nano Energy (2023)[8]	Triboelectric (Out-of-plane)	CMEO with matching capacitor	Peak	Triple MOSFET Structure	Yes (Immediate start)	0	<300 V	1~3 Hz	LC buck	No	N/A	N/A	P_{unreg}/P_{in}^{*}= 79.3 % @ 6.2 V
Nano Energy (2024)[9]	Triboelectric (Out-of-plane)	CMEO with matching capacitor	Peak	BJT-based peak-tracking	Yes (Immediate start)	0	<200V	N/A	LC buck	No	N/A	N/A	$P_{sw}/P_{AC,opt}$ = 88.2% @ C_{in}=220 pF
IEEE Transactions on Circuits and Systems I[10]	Triboelectric (Out-of-plane)	CMEO	Peak	0.18 μm CMOS IC controller + low-side N-MOSFET	Yes (Delayed start)	1.56 μW	<300 V	1~5 Hz	Flyback	No	N/A	N/A	P_{unreg}/P_{CEMO} = 51.2% @ 7.56 V
ISSCC (2018)[11]	Triboelectric (Out-of-plane)	FBR*+MPPT*	Threshold	Embedded switch in 0.18 μm BCD* IC	Yes (Delayed start)	N/A	70 V	N/A	LC buck	No	N/A	N/A	P_{unreg}/P_{in} = 51.1 % @ 4.7 V
2019 Symposium on VLSI Circuits (2019)[12]	Triboelectric (N/A)	FBR+MPPT	Threshold	Embedded switch in 0.18 μm BCD IC	Yes (Delayed start)	361.6 nW	70 V	N/A	Dual input buck	N/A	N/A	N/A	P_{unreg}/P_{in} = 70.72% @ P_{in} = 20.7 μW
Joule (2019)[13]	Triboelectric (Out-of-plane)	CMEO	Threshold	Air-discharge switch	Yes (Immediate start)	0	7.5 kV	1~3 Hz	Flyback	No		N/A	$P_{unreg}/P_{AC,opt}$ 78.5% @ 15 V
Advanced Science (2024)[3]	Triboelectric (Out-of-plane)	CMEO with matching capacitor	Threshold	SCR-based switch	Yes (Immediate start)	0	1790 V (fixed)	N/A	LC buck	Battery (2.7-4.41 V)	0.56-0.67*(1) @ 2.7-4.4 V	N/A	N/A
Nature Communications (2024)[2]	Triboelectric (In-plane)	CMEO	Threshold	Needle-discharge switch	Yes (Immediate start)	0	~2500 V (fixed)	N/A	LC buck	DC-DC (1.8 V)	0.40*(2) @ 1.8 V	N/A	N/A
Energy Environ. Sci. (2025)[14]	Triboelectric (Out-of-plane)	CMEO with matching capacitor	Threshold	SCR-based switch	Yes (Immediate start)	0	N/A	N/A	LC buck	Hysteresis regulation (1.7-2.8 V)	0.21*(3) @ 1.7-2.8 V	N/A	N/A
Energy Environ. Sci. (2025)[4]	Triboelectric (In-plane)	N/A	N/A	N/A	N/A	N/A	N/A	~ 1364 Hz	Inductive transformer	Battery (2.8-4 V)	0.63-0.91*(4) @ 2.8-4 V	N/A	$P_{unreg}/P_{AC,opt}$ = 83.74% @ 3.21 V

IEEE Transactions on Power Electronics (2024)[15]	Triboelectric (Out-of-plane)	MR-SCE*	Peak	Discrete controller + MOSFETs	No	N/A	142 V	2 Hz	Switch capacitor convertor	No	N/A	N/A	P_{unreg}/P_{CMEO} 22.3 % @ 18 V
IEEE Journal of Solid-State Circuits (2022)[16]	Triboelectric (Out-of-plane)	SSHI*	Zero-Cross Detection	Embedded switch in 0.18 µm BCD IC	Yes (Delayed start)	N/A	<195 V	N/A	Switch Capacitor convertor	Battery (2.7-4.2 V)	N/A	0.38*(5) @ 3.6 V	P_{reg}/P_{in} = 70.7 % @ 3.6 V
IEEE Transactions on Industrial Electronics (2023)[17]	Triboelectric (Out-of-plane)	SSHI	Peak	0.18 µm BCD IC controller + low-side N-MOSFET	Yes (Delayed start)	N/A	<65 V	200~400 Hz	Switch Capacitor convertor	Battery (2.7-4.2 V)	N/A	0.32*(6) @ 3.6 V	P_{reg}/P_{in} = 79.3% @ 3.6 V
Journal of Intelligent Material Systems and Structures (2022)[18]	Electret (In-plane)	SSHI	Peak	Discrete controller with low-side MOSFET	Yes (Delayed start)	6 µW	<240 V	~109 Hz	N/A	No	N/A	N/A	$P_{unreg}/P_{FBR,\ opt}$ = 1.22 @ 3 V
Electronics Letters (2018)[19]	Triboelectric (Out-of-plane)	Bennet doubler	N/A	N/A	N/A	N/A	N/A	N/A	N/A	No	N/A	N/A	$P_{unreg}/P_{FBR,\ opt}$ 152.3 @ 256 V
Nano Energy (2018)[20]	Triboelectric (Out-of-plane)	Bennet doubler	N/A	N/A	N/A	N/A	N/A	N/A	N/A	No	N/A	N/A	$P_{unreg}/P_{FBR,\ opt}$ 2.5 @ 380 V
IEEE Transactions on Industrial Electronics (2023)[21]	Thermoelectric Photovoltaic Piezoelectric	MPPT	N/A	N/A	N/A	N/A	2.2-3.2 V 2.6-3.8 V 3-4.2 V	N/A	Discrete MISIMO* converter 7-19;0-1.8; 2.2-4.2 V		N/A	N/A	P_{reg}/P_{in} = 92.5%
ISSCC (2022)[22]	Thermoelectric Photovoltaic Piezoelectric	MPPT SECE*	N/A	N/A	Yes (Delayed start)	N/A	<5 V	N/A	65nm CMOS MISIMO converter 0.6/1.2/3.3 V		N/A	N/A	P_{reg}/P_{in} = 80%
ISCAS (2022)[23]	Piezoelectric	SSHI	Zero-cross Detection	N/A	N/A	N/A	V_{PEH} = 1.7 V	250 Hz	0.18µm CMOS Switch capacitor DC-DC converter 0.5/1/2 V		N/A	N/A	P_{reg}/P_{in} = 0.894 (Simulation)
This work	Electret (In-plane)	CMEO	Peak	Positive-feedback-based discrete switch	Yes (Immediate start)	0	<1200 V	12 ~ 120 Hz	LC buck	DC–DC + Automatic Power Allocation (1.5/2.7/5 V)	1.08 @ 5 V	0.45	$P_{unreg}/P_{AC,opt}$ = 1.48 @401 V; $P_{unreg}/P_{FBR,opt}$** =2.4; P_{unreg}/P_{CMEO} =0.6; P_{reg}/P_{unreg} = 93.4% @ V_{unreg} = 12-18 V; P_{reg}/P_{sw}** = 79.3%; $P_{reg}/P_{FBR,opt}$ =1.78

*: CMEO (cycle for maximized energy output); FBR (Full-wave bridge rectifier); MPPT (Maximum power point tracking); MR-SCE (multi-release synchronized charge extraction); SSHI (synchronized switch harvesting on inductor); SECE (synchronous electric charge extraction); BCD (bipolar-CMOS-DMOS); MISIMO (multi-input single-inductor multi-output).

**: Power definitions: P_{reg} (average power with regulated DC output); $P_{AC,opt}$ (optimal average power with AC output); P_{unreg} (maximum average power with unregulated DC output); $P_{FBR+buck}$ (average power with full-wave bridge rectifier and buck converter); P_{in} (input power to the power management system); $P_{FBR,\ opt}$ (optimal output power under full bridge rectification); P_{sw} (average power output with peak switch); P_{CMEO} (output power of generator operating in CMEO. For a generator with time-independent capacitance and symmetrical output, P_{CMEO} satisfies the relation $P_{CMEO} = 4\ P_{FBR,opt}$); $P_{chg,bat}$ (average battery-charging power).

*(#) Estimated values: (1) $P_{chg,bat}$ = 1.3 mW @ 2.7 V, $P_{chg,bat}$ = 1.56 mW @ 4.4V, $P_{AC,opt}$ = 2.32 mW; (2) P_{reg} = 6.9 mW @ 1.8 V, $P_{AC,opt}$ = 17.31 mW; (3) P_{reg} = 0.67 mW, $P_{AC,opt}$ = 3.1 mW; (4) $P_{chg,bat}$ = 42 mW @ 2.8 V, $P_{chg,bat}$ = 60 mW @ 2.8 V, $P_{AC,opt}$ = 66.14 mW; (5) P_{in} = 823.3 µW, P_{CMEO} = 4*$P_{FBR,opt}$ = 1540.8 µW, P_{reg} / P_{CMEO} = P_{reg} / P_{in} * P_{in}/P_{CMEO} = 70.7% * 823.3 / 1540.8;(6) V_{OC} = 150V, V_R = 60V, β = 1, P_{reg} / P_{CMEO} = P_{reg} / P_{in} * P_{in} / P_{CMEO} = 79.3% * 60 / 150.
